# A proxy for oxygen storage capacity from high-throughput screening and automated data analysis†

**DOI:** 10.1039/d3sc03558a

**Published:** 2023-10-23

**Authors:** Jack J. Quayle, Alexandros P. Katsoulidis, John B. Claridge, Andrew P. E. York, David Thompsett, Matthew J. Rosseinsky

**Affiliations:** a Department of Chemistry, University of Liverpool Crown Street Liverpool L69 7ZD UK m.j.rosseinsky@liverpool.ac.uk; b Johnson Matthey Technology Centre Blounts Court Road Reading RG4 9NH UK

## Abstract

Oxygen storage and release is a foundational part of many key pathways in heterogeneous catalysis, such as the Mars-van Krevelen mechanism. However, direct measurement of oxygen storage capacity (OSC) is time-consuming and difficult to parallelise. To accelerate the discovery of stable high OSC rare-earth doped ceria-zirconia oxygen storage catalysts, a high-throughput robotic-based co-precipitation synthesis route was coupled with sequentially automated powder X-ray diffraction (PXRD), Raman and thermogravimetric analysis (TGA) characterisation of the resulting materials libraries. Automated extraction of data enabled rapid trend identification and provided a data set for the development of an OSC prediction model, investigating the significance of each extracted quantity towards OSC. The optimal OSC prediction model produced incorporated variables from only fast-to-measure analytical techniques and gave predicted values of OSC that agreed with experimental observations across an independent validation set. Those measured quantities that feature in the model emerge as proxies for OSC performance. The ability to predict the OSC of the materials accelerates the discovery of high-capacity oxygen storage materials and motivates the development of similar high-throughput workflows to identify candidate catalysts for other heterogeneous transformations.

## Abstract 2

A quantitative proxy model for the slow-to-measure oxygen storage capacity was developed using only fast-to-measure metrics taken from a workflow consisting of high-throughput synthesis, high-throughput screening techniques and automated analysis.

## Graphical abstract

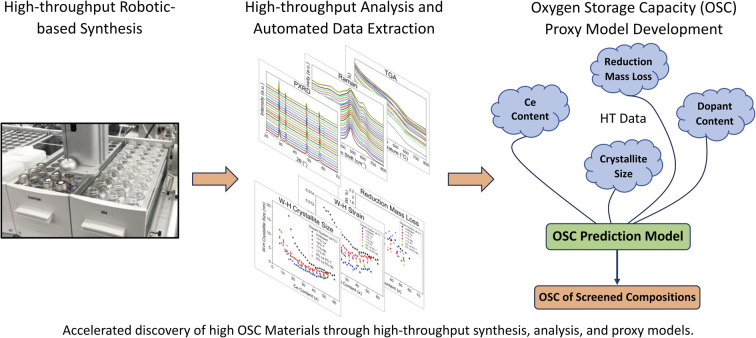

## Introduction

Ceria-based materials are useful in a wide range of applications, including solid oxide fuel cells,^1^ supports, and catalysts,^2^ through the ability of the ceria to store and release oxygen *via* the Ce^3+^/Ce^4+^ redox couple:^3^
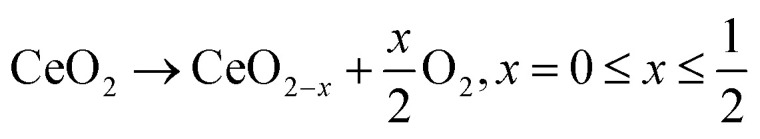


The oxidised ceria is able to provide the oxygen needed for reactions such as carbon monoxide and hydrocarbon oxidation,^3^ with the arising reduced ceria being able to store oxygen, promoting reduction reactions. The total amount of oxygen stored and released by ceria in this way is the oxygen storage capacity (OSC), which gives an insight into the performance of the catalyst. The importance of oxygen storage extends beyond ceria-based materials to other redox oxide catalyst systems which typically adopt the Mars-van Krevelen mechanism.^4–6^ Oxygen storage is useful in allowing both oxidation and reduction reactions when operating under varying conditions, such as the fluctuating air/fuel exhaust gas ratios with three-way catalysts,^7^ where temperatures of 800–900 °C or higher can be experienced during operation.^8^ However, the performance of cerias is hindered by a lack of thermal stability at these high temperatures.^9^ This is addressed by the substitution of smaller zirconium ions into the fluorite structure of ceria, which improves both the thermal stability and the OSC.^10–13^ Studies have shown that the best catalytic performance is obtained at a Ce/Zr ratio of around one,^14,15^ where there is a balance between thermal stability and available oxygen. If the content of Zr is higher, the total capacity of Ce to store and release oxygen is limited, and if the Zr content is lower, the materials will be less thermally stable and have a lower proportion of Ce reduced, due to reduced lattice distortion.^10,11,14–16^ Doping ceria-zirconia materials with rare-earth elements has been shown to further improve key properties, through creation of oxygen vacancies, improving the OSC at dopant amounts below 10 at%.^17,18^ The porosity^18^ and thermal stability of the materials are also improved, in both single and mixed dopant systems.^18–21^ The rare-earth elements lanthanum, yttrium, praseodymium, and neodymium are known to result in enhanced thermal stability through the retention of a single phase after ageing,^19,22^ whereas undoped materials give mixed phase materials possessing Ce-rich and Zr-rich components.^16,19,22^ Since doping with rare-earths gives more thermally stable materials possessing high catalytic activity,^18,19^ these dopant systems will be investigated within this high-throughput study.

Acceleration of catalyst materials discovery through the identification of proxies for the time-consuming evaluation of catalyst performance that can be rapidly measured at scale has been demonstrated for stable and active Fischer–Tropsch catalysts.^23^ From large arrays of samples, catalysts were identified utilising X-ray diffraction to probe particle size, which related to activity and selectivity. The persistence of the catalytic activity and selectivity under reaction conditions indicated stability and strengthened the proxy for active metal surface area when coupled with high-throughput temperature programmed reduction.^23^ The proxies were employed to identify hits (the more promising catalytic materials that were subsequently investigated in detailed serial testing), but not to build a predictive model for catalytic performance. They were based on a previously established correlation between particle size and activity in the Fischer–Tropsch reaction.^24^

OSC measurements are slow and difficult to automate within a high-throughput workflow. The aim of this work was to accelerate the discovery of stable, high oxygen storage ceria-zirconia materials by implementing a high-throughput robotic-based co-precipitation pathway that generates libraries of materials that can afford characterisation data at scale, from rapid automated materials measurement techniques such as PXRD, Raman and TGA. By building a predictive model that connects the measured OSC of a set of test materials to metrics derived from this characterising data, the OSC of the library compositions can be efficiently screened to focus the slower OSC measurement capacity on the highest performing materials, moving beyond proxy-based filters to quantitative performance prediction of candidate catalysts.

## Results and discussion

### Workflow development

**Fig. 1:**
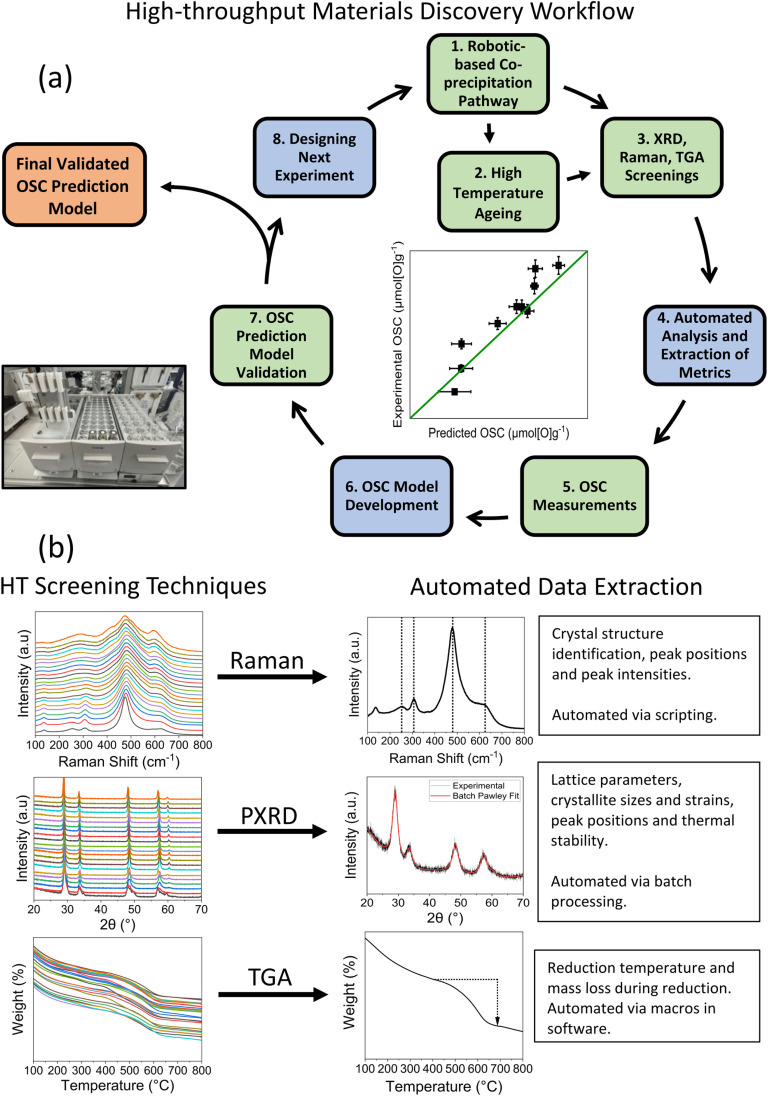
(a) Workflow for the robotic-based co-precipitation pathway and subsequent high-throughput (HT) screening (detailed explanation in ESI Section A†). Green boxes are experimental steps, blue are computational steps and orange is the output of the workflow. (b) The analysis workflow for each of the high-throughput screening techniques is given here, with examples of the extracted quantities from each technique.

The high-throughput materials discovery workflow implemented in this work is presented in Fig. 1. A robotic-based synthesis route was implemented to synthesise ceria-zirconia materials *via* a co-precipitation pathway. This utilised a robotic liquid handling instrument (Gilson GX-281) which automated the combination of stock solutions, the addition of precipitant and the shaking of vials using an orbital shaker. Samples were obtained by centrifugation and drying in the centrifuge tubes, before grinding to a powder and calcining in ceramic well plates (a detailed experimental explanation is in ESI Section A†). High-throughput PXRD was measured by utilising a robotic arm to load and unload samples for sequentially automated measurements. High-throughput Raman spectra was obtained by using a 384-well plate with microplate mapping, automatically sequentially measuring the spectra of each sample. For high-throughput TGA measurements, samples were loaded onto platinum pans and a defined experiment was ran for each sample in an automated sequential system. Materials were also subjected to a high temperature ageing treatment, at 1100 °C for 4 h in air, to investigate the thermal stability of the materials through PXRD and Raman measurements, where thermally stable materials retained a single phase after the high temperature treatment. 333 compositions were synthesised and measured, with 215 (65%) proving stable to the ageing step.

The fast-to-measure data allowed the selection of samples for OSC measurements, spanning a wide range of the measured quantities. These OSC results were used to develop an OSC prediction model, investigating the relationship between the wide range of variables extracted from the screening measurements with the measured OSC of the materials.

**Fig. 2:**
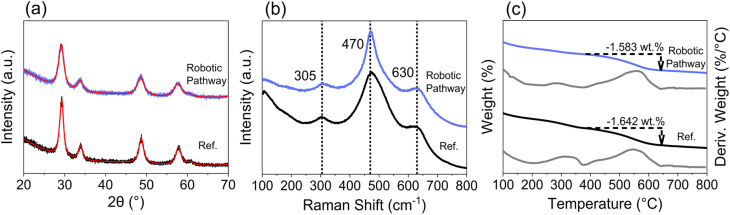
Comparison of a Ce_0.5_Zr_0.5_O_2−*δ*_ material synthesised *via* the robotic-based co-precipitation pathway (blue) implemented in this study with a Ce_0.5_Zr_0.5_O_2−*δ*_ reference material (black). The (a) PXRD patterns, with Pawley fits from automated batch analyses (red lines show the fit for the *Fm*3̄*m* space group), (b) Raman spectra (normalised to the intensity of the peak at 470 cm^−1^), (c) TGA profiles (blue/black profiles) and derivative curves of weight with temperature (grey profiles), during a ramp in temperature under 5% H_2_/N_2_, are given for both materials.

For a robotic-based high-throughput pathway to be useful in the accelerated discovery of high performing catalysts, it must produce materials that are representative of those obtained by conventional synthesis routes. Fig. 2 gives a comparison of the PXRD patterns, Raman spectra and TGA profiles collected for a fresh Ce_0.5_Zr_0.5_O_2−*δ*_ material produced by the robotic route with those obtained from an industrial Ce_0.5_Zr_0.5_O_2−*δ*_ reference, provided by Johnson Matthey PLC. The PXRD pattern (Fig. 2a) of the material produced using the robotic pathway is similar to the reference material, giving broad single peaks, confirming the formation of a single-phase solid solution. Since it is difficult to distinguish between the cubic (c), pseudo-cubic (t′′), and tetragonal (t) phases of ceria-zirconia by PXRD alone, due to the small crystallite sizes giving broad peaks, and the low atomic scattering factor of oxygen, Raman spectroscopy is important for identifying the phases present.^25^ The Raman spectra for the robotic sample and reference (Fig. 2b) both exhibit three peaks at about 305 cm^−1^, 470 cm^−1^ and 630 cm^−1^. The intense peak at around 470 cm^−1^ (F_2g_) is characteristic of the cubic phase of ceria-zirconia materials.^25^ The small peak at around 305 cm^−1^ is assigned to displacement of the lattice oxygens from the ideal fluorite positions and indicates the presence of a pseudo-cubic (t′′) phase when observed along with the peak at about 470 cm^−1^.^14,15^ Tetragonal crystal structures show peaks at about 250, 300, 470 and 600 cm^−1^.^26^ The peak at around 600 cm^−1^ is attributed to defects and oxygen vacancies, and is observed for all cubic, pseudo-cubic and tetragonal crystal structures.^25,27^ Therefore, the Raman spectra in Fig. 2b shows that the material produced by the robotic pathway and the reference material demonstrate a pseudo-cubic (t′′) phase. In Fig. 2c, the TGA profiles collected during a ramp in temperature under 5% H_2_/N_2_ show that the robotic and reference materials have similar reduction temperature, calculated as the temperature of the peak in the derivative TG curve of weight with temperature (plotted as grey lines in Fig. 2c), and both samples give a similar mass loss during reduction, calculated as the mass lost between the onset and offset temperature of the peak in the differential TG curve, shown by the dotted arrow in Fig. 2c.

A comparison of the Williamson–Hall crystallite size, strain, porosity and OSC of the robotically synthesised Ce_0.5_Zr_0.5_O_2−*δ*_ material with the reference is given in Table 1. The materials possess similar surface areas but differ in terms of crystallite size and strain. Crucially, the OSC of the materials are similar, which was also confirmed across the entire range of Ce content in Ce_*x*_Zr_1−*x*_O_2−*δ*_ materials, for 0 < *x* ≤ 1 (Fig. S7†). The similarities observed between the sample synthesised *via* the robotic pathway and the reference in terms of structure, reducibility and oxygen storage inspires confidence in the use of the robotic-based synthesis route to produce representative materials, which can now be utilised for high-throughput screening and OSC prediction model development.

**Table 1:** A comparison of the structural, textural and OSC properties of a Ce_0.5_Zr_0.5_O_2−*δ*_ material synthesised using the robotic-based co-precipitation route with a Ce_0.5_Zr_0.5_O_2−*δ*_ reference material

Sample	Williamson–Hall crystallite size (nm)	Williamson–Hall strain	Surface area (m^2^ g^−1^)	Pore volume (cm^3^ g^−1^)	Average pore size (nm)	OSC (μmol[O]g^−1^)
Reference material	19(2)	0.01108(24)	78(1)	0.140(1)	7.7(1)	831(42)
Robotic-based synthesis	8.1(3)	0.00838(29)	86(1)	0.109(2)	5.5(3)	752(38)

### Screening for stability, structure and reducibility

**Fig. 3:**
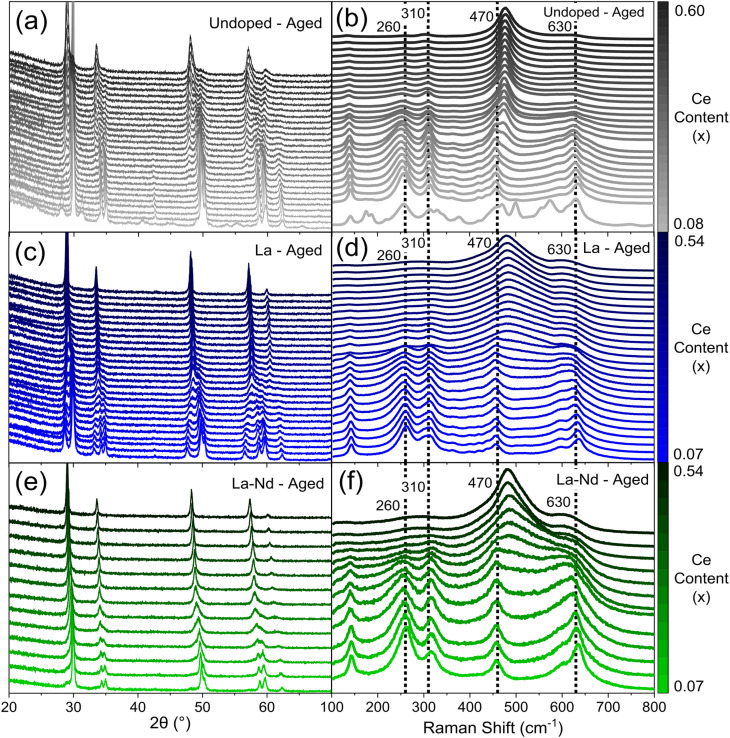
The scale of the raw data extracted from PXRD and Raman screenings is provided for a sub-set of thermally aged Ce_*x*_Zr_1−*x*−*y*_Ln_*y*_O_2−*δ*_ materials, where the stated lanthanide dopant content (*y*) is fixed while the Ce content (*x*) is varied. (a, c and e) PXRD patterns and (b, d and f) Raman spectra (normalised to intensity of the F_2g_ peak at 470 cm^−1^) are given for (a and b) undoped (*y* = 0, 0.08 ≤ *x* ≤ 0.60), (c and d) 11% La doped (*y* = 0.11, 0.07 ≤ *x* ≤ 0.54), and (e and f) 5% La 5% Nd doped (*y* = 0.10, where Ln_*y*_ = La_0.05_Nd_0.05_ as there are two dopants, 0.07 ≤ *x* ≤ 0.54) materials. The full fresh and aged PXRD patterns and Raman spectra of all dopant systems (undoped, La, Y, Pr, Nd, La–Y and La–Nd) are provided in ESI Fig. S8, S9, S11 and S12.†

Since the Ce/Zr ratio has been shown to influence the structure, thermal stability, reducibility, and catalytic activity of ceria-zirconia materials,^28,29^ a library of materials covering a range in Ce/Zr ratio were synthesised, examining both single (La, Y, Pr and Nd) and mixed dopant (La–Y and La–Nd) systems (full compositional ranges are provided in ESI Table S1†), at total dopant concentrations between 9 at% and 15 at%. A subset of the data collected on the compositions produced is given in Fig. 3, giving an insight into the scale of data that is obtained from the high-throughput screening process (the full PXRD patterns and Raman spectra are provided in ESI Fig. S8, S9, S11 and S12†). A library of materials where the dopant content in these single and mixed rare-earth dopant systems is varied was also investigated for both single (La, Y, Pr and Nd) and mixed dopant (La–Y and La–Nd) systems, keeping the Ce/Zr ratio constant at one, since previous studies have shown that the best catalytic performance is obtained at a Ce/Zr ratio of around one^14,15^ (full compositional ranges are provided in ESI Table S2†).

**Fig. 4:**
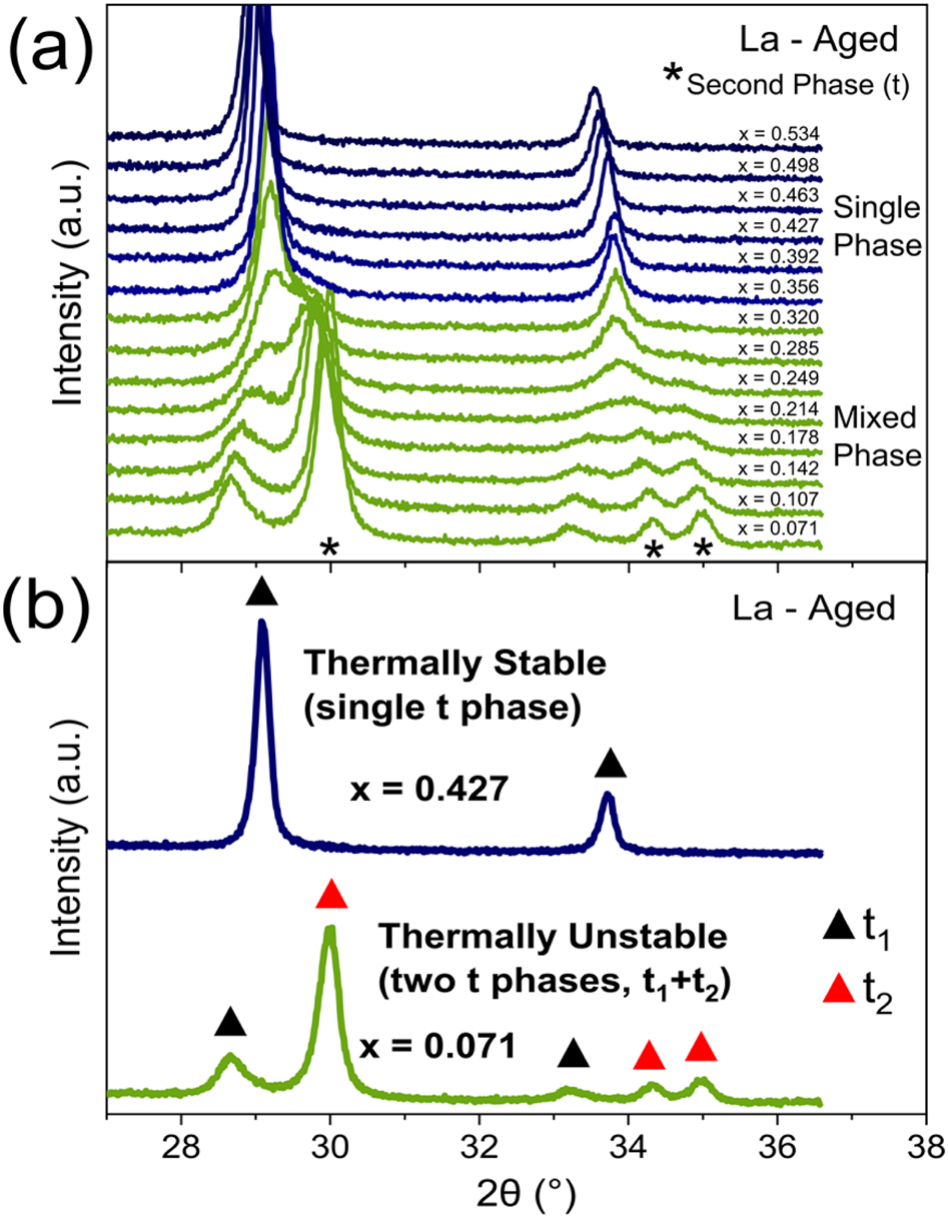
The impact of varying the Ce content within thermally aged 11 at% La doped materials, with the general formula Ce_*x*_Zr_0.89−*x*_La_0.11_O_2−*δ*_, on the phase assemblage and thermal stability is identified through the PXRD patterns. (a) When varying the Ce content from *x* = 0.534 to *x* = 0.071, a single phase to mixed phase transition is observed within the PXRD, with single phase patterns in blue and mixed phase patterns in green. The additional PXRD peaks of the second phase in the mixed phase materials are marked by an asterisk (*). The process of identifying thermally stable materials is demonstrated in (b), with a single phase PXRD pattern in blue (for Ce_0.427_Zr_0.463_La_0.11_O_2−*δ*_), which is thermally stable, and a mixed-phase PXRD pattern in green (for Ce_0.071_Zr_0.819_La_0.11_O_2−*δ*_), which is thermally unstable. The exact phase symmetry is identified by the Raman spectra of these materials, provided in Fig. 3d. Black triangles (▲) denote the PXRD peaks corresponding to one tetragonal phase (t_1_) and red triangles (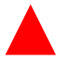) denote the PXRD peaks corresponding to another tetragonal phase (t_2_) where applicable. The full PXRD patterns of the aged materials for all dopant systems (undoped, La, Y, Pr, Nd, La–Y and La–Nd) are provided in ESI Fig. S8 and S9. †

PXRD is used to identify thermally stable materials after a high temperature thermal ageing treatment at 1100 °C for 4 h in air. An example for 11 at% La doped materials (Ce_*x*_Zr_0.89−*x*_La_0.11_O_2−*δ*_) after ageing is given in Fig. 4a, where there is a transition from a single phase to a mixed phase system when decreasing Ce content below *x* = 0.356, identified by new peaks in the PXRD patterns corresponding to a second phase. The materials demonstrating multiple phases after the thermal ageing treatment are thermally unstable and can be eliminated from the stable materials discovery process. The selection process is shown in Fig. 4b, where a representative PXRD pattern for both a thermally stable and a thermally unstable material is given. For the libraries of materials screened in this study, 35% of samples were identified as being thermally unstable, and the exact compositional ranges categorised as being thermally stable are listed in ESI Tables S1 and S2.†

**Fig. 5:**
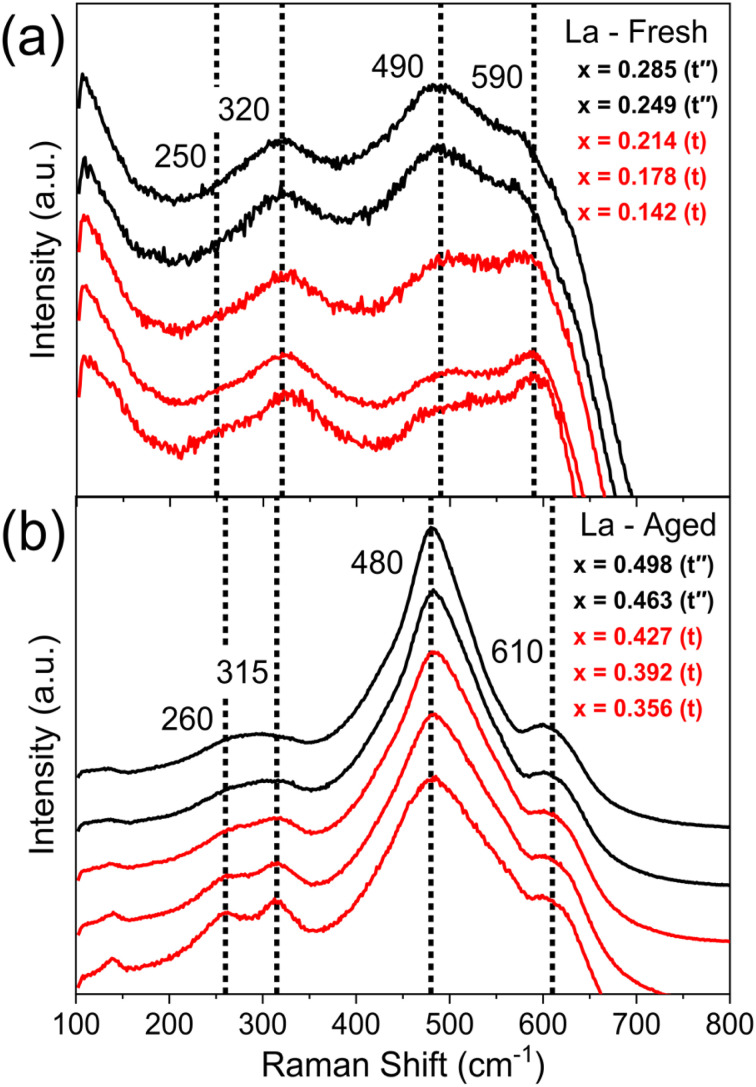
Raman spectra of fresh and thermally aged materials with the general formula Ce_*x*_Zr_0.89−*x*_La_0.11_O_2−*δ*_, across the range in Ce content (*x*) that shows the phase transition from a pseudo-cubic phase (t′′) (black) to a tetragonal phase (t) (red). In fresh samples (a), the transition occurs between 0.214 ≤ *x* ≤ 0.249 and in aged samples (b) the transition occurs between 0.427 ≤ *x* ≤ 0.463. Vertical dotted lines show the Raman shift values associated with each of the peaks that are observed for tetragonal materials, at 250, 320, 490 and 590 cm^−1^. The full Raman spectra for the fresh and aged materials across the whole Ce content range are given in ESI Fig. S11.† The full PXRD patterns measured on these compositions are given in ESI Fig. S8.†

**Fig. 6:**
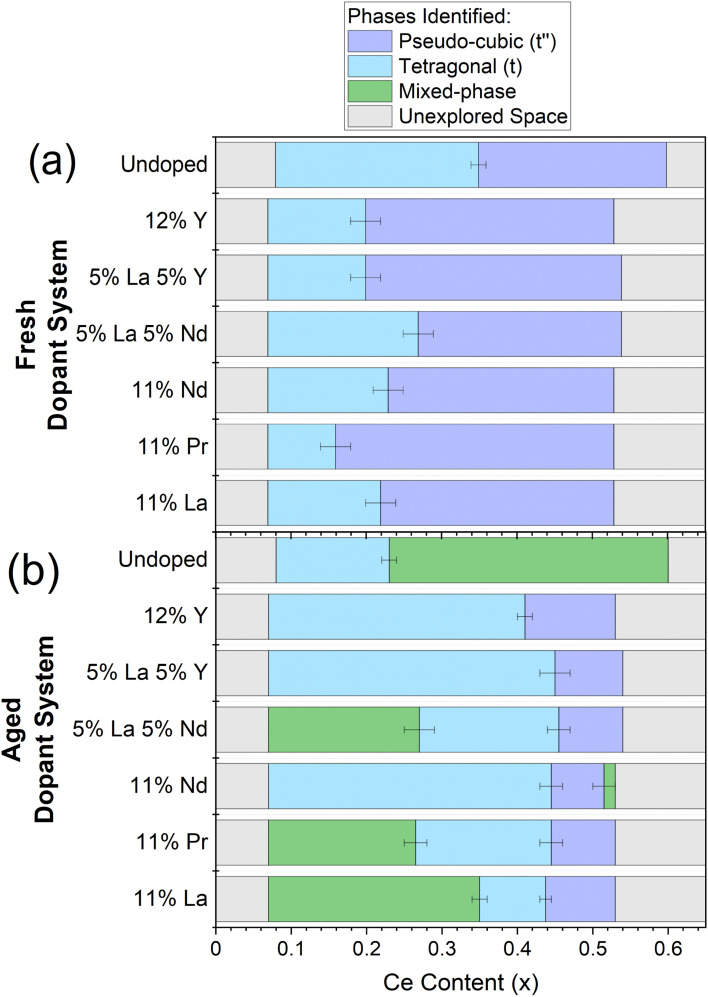
The evaluation of the phases present for the library of (a) fresh and (b) thermally aged Ce_*x*_Zr_1−*x*−*y*_Ln_*y*_O_2−*δ*_ materials, where the stated lanthanide dopant content (*y*) is fixed and the Ce content (*x*) is varied, for the single and mixed dopant systems listed on the *y*-axis across 0.00 < *x* < 0.65. The dopant systems are ordered by the mean ionic radii from smallest (undoped) to largest (11% La). The coloured bars indicate the phase symmetry identified by Raman for materials that demonstrated a single phase PXRD pattern and marks the materials which demonstrated mixed phase PXRD patterns. Single phase pseudo-cubic (t′′) materials are marked with purple, single phase tetragonal (t) materials with blue, mixed phase materials with green and unexplored space with grey. Errors are given as horizonal lines at the phase boundaries. The full PXRD patterns and Raman spectra of the fresh and aged materials for each dopant system in this plot are given in ESI Fig. S8 and S11,† respectively.

Once single and mixed phase materials have been distinguished by PXRD, for both fresh and aged samples, the Raman spectra were used to identify the exact phases in each case, exploiting the greater sensitivity of Raman to small changes in metric symmetry. Fig. 5 shows the Raman spectra for 11 at% La doped materials (Ce_*x*_Zr_0.89−*x*_La_0.11_O_2−*δ*_), where a pseudo-cubic (t′′) to tetragonal (t) phase transition is observed in the Raman spectra, through the appearance of a peak near 250 cm^−1^ coupled with the reduction in intensity of the peak near 490 cm^−1^, characteristic of tetragonal phase materials.^26,27,30^ The pseudo-cubic to tetragonal phase transition occurs between 0.214 ≤ *x* ≤ 0.249 for the fresh materials, and between 0.427 ≤ *x* ≤ 0.463 for the thermally aged materials. This phase transition is observed for all dopant systems screened and Fig. 6 shows the Raman-identified phases across the entire compositional space screened for each dopant system studied, where the stated lanthanide dopant content (*y*) is fixed and the Ce content (*x*) is varied for materials with the general formula Ce_*x*_Zr_1–*x*−*y*_Ln_*y*_O_2−*δ*_. In Fig. 6a, for the fresh materials, undoped ceria-zirconia shows the pseudo-cubic to tetragonal phase transition near *x*= 0.35, in agreement with a previous report,^30^ and all single and mixed dopant systems demonstrate the pseudo-cubic to tetragonal phase transition at a lower Ce content than undoped materials. In Fig. 6b, after thermal ageing, all of the single and mixed dopant systems demonstrate mixed-phase materials across a smaller proportion of the Ce content range than the undoped materials, highlighting the ability of the rare-earth dopants to improve thermal stabilities, contributing to better catalytic materials. The contribution of rare-earth dopants to giving better catalytic materials is further demonstrated by the ability of all the dopant systems to give thermally stable materials around the Ce/Zr ratio of 1 (0.44 ≤ *x* ≤ 0.45), where the highest OSC values are observed.^14,15^

Having identified the crystalline phase of each sample by Raman, the PXRD patterns were fitted with the correct space group to extract lattice parameters. Crystallite sizes and strains of each sample were also obtained from the PXRD patterns using Williamson–Hall analysis. Increasing the rare-earth dopant content (for La, Y, Pr, Nd, La–Y and La–Nd systems) increased lattice parameters, as shown in ESI Fig. S16,† indicating that the dopant cations are entering the lattice of the solid solutions. The lattice parameters extracted for systems varying the Ce content at constant dopant content are provided in ESI Fig. S14 and S15.†

It is important that the input and output metal ratios present in the prepared materials are close to identical. The increasing lattice parameters with dopant content in ESI Fig. S16† indicates this along with the presence of a single-phase in the PXRD patterns (the PXRD patterns are provided in ESI Fig. S10†). To further investigate this, ICP was carried out on the supernatant from the precipitation after centrifugation, showing that 0% of the overall Zr content was lost and 1.03 × 10^−4^% of the overall Ce content was lost (given in ESI Table S3†). SEM images and SEM-EDS compositional information were gathered for two Y-doped materials produced using the robotic method and are provided in ESI Fig. S17.† The images of the particles for each sample demonstrated homogeneity and the SEM-EDS analyses indicated the homogenous expected composition for both of the materials.

**Fig. 7:**
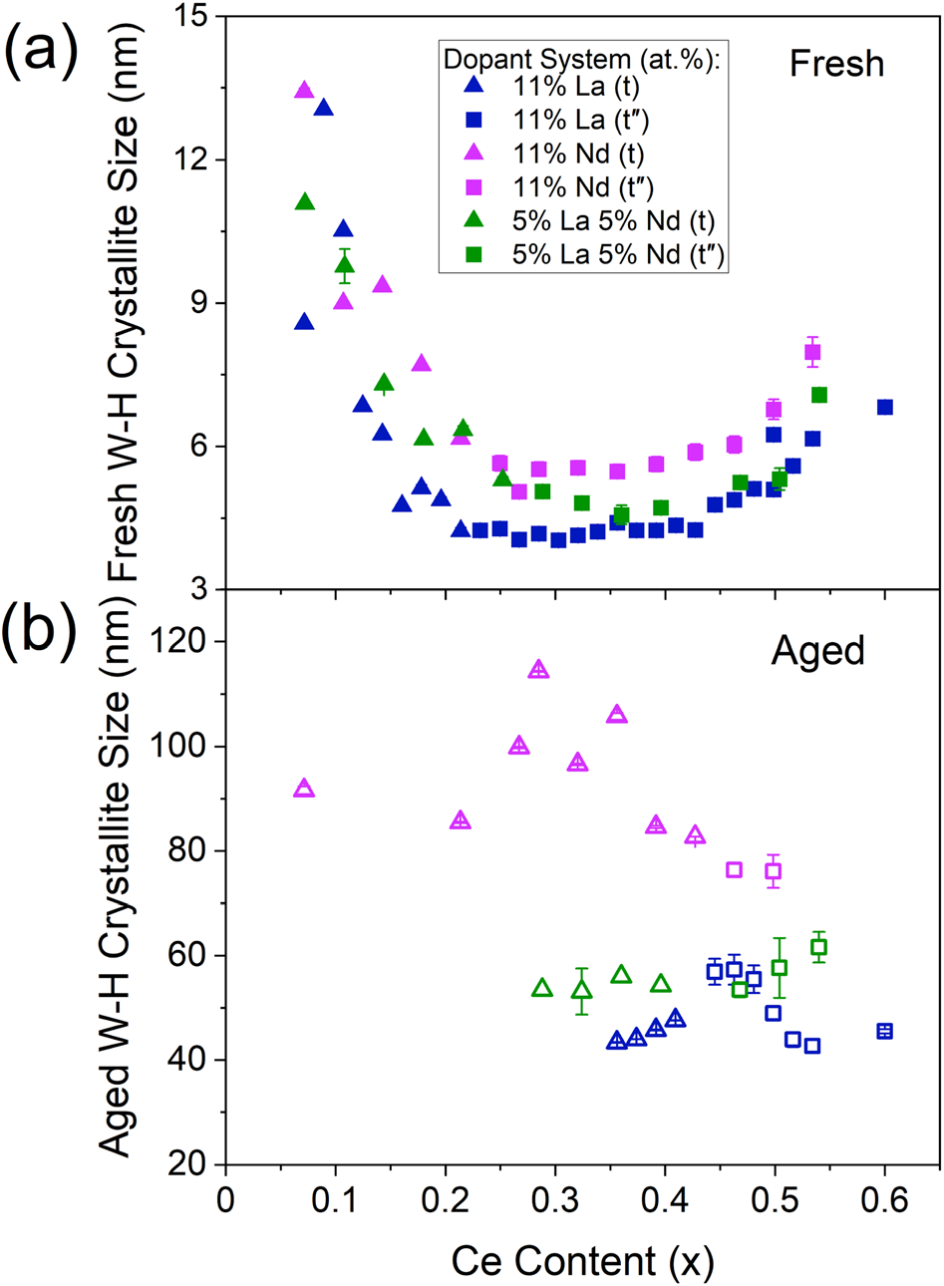
The trend of Williamson–Hall crystallite sizes with Ce content for the (a) fresh and (b) thermally aged 11 at% La and 11 at% Nd single doped materials when compared to mixed dopant La–Nd doped materials (5 at% of each dopant), with the general formula Ce_*x*_Zr_1−*x*−*y*_Ln_*y*_O_2−*δ*_, where the stated lanthanide dopant content (*y*) is fixed and the Ce content (*x*) is varied between 0.00 < *x* ≤ 0.60. The shape of the plotted points indicates the phase of the materials as identified by Raman (triangles are tetragonal (t), squares are pseudo-cubic (t′′), and the full Raman spectra are given in ESI Fig. S11†). Similar plots including Nd, Pr, Y and La–Y doped materials are given in ESI Fig. S18 to S21 (Section C7).†

Evaluation of the derived Williamson–Hall crystallite sizes shows that the mixed dopant systems possess crystallite sizes intermediate to those of the respective single dopant systems across the Ce content range of 0 at% to 60 at% Ce, while keeping dopant content constant. An example is given in Fig. 7 for La, Nd, and La–Nd doped materials, where the Williamson–Hall crystallite sizes of the mixed dopant system (containing an equal amount of each dopant) lies between that of the single dopant systems, for both the fresh and thermally aged materials. This is true for both La–Y and La–Nd dopant systems when compared to those of La, Y and Nd single doped materials, shown in ESI Fig. S18.† La–Y and La–Nd systems containing unequal dopant amounts were also investigated. When increasing the amount of La in the La–Nd system, keeping the Ce/Zr ratio and Nd content constant (Fig. S21†), the materials contained crystallite sizes intermediate to those of the respective single dopant systems. The materials with a higher proportion of La than Nd possessed smaller crystallite sizes across the Ce content range than Nd-rich La–Nd systems, closer to the single La doped materials than the single Nd doped materials. A similar trend is observed for La–Y doped materials, keeping the Ce/Zr ratio and La content constant (Fig. S21†), where the materials with a higher proportion of Y than La possessed crystallite sizes closer to the single Y doped materials than single La doped materials. Since the Williamson–Hall crystallite size relates to the dopant system present in the cases described above with both La–Nd and La–Y dopant systems, with both equal and unequal amounts of each dopant, the crystallite size data contains information on the dopant system present.

**Fig. 8:**
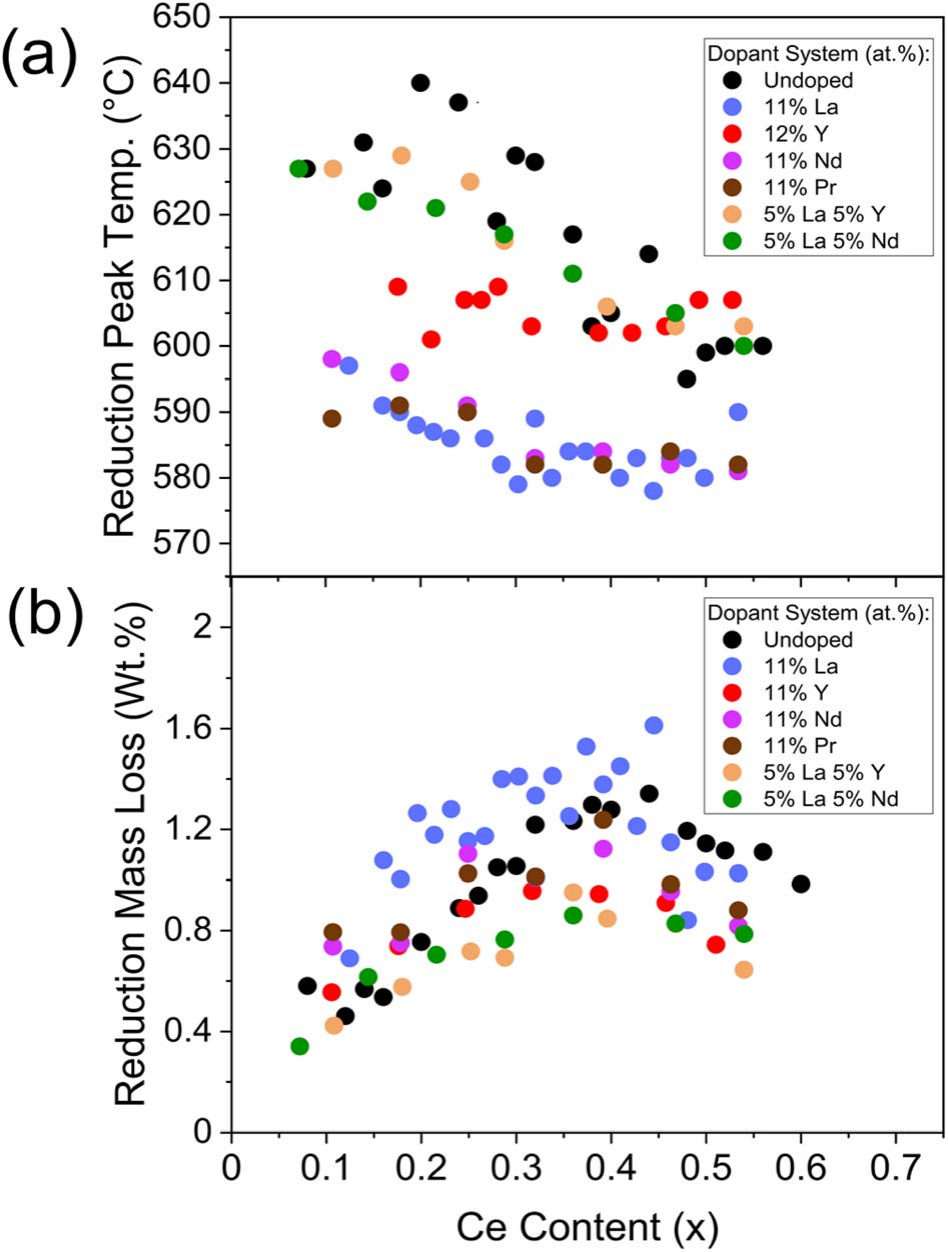
The evaluation of the (a) reduction peak temperature and (b) mass lost during reduction from TGA measurements against Ce content, for materials with the general formula Ce_*x*_Zr_1−*x*−*y*_Ln_*y*_O_2−*δ*_, where the stated lanthanide dopant content (*y*) is fixed and the Ce content (*x*) is varied between 0.00 < *x* < 0.65. Undoped, single doped (La, Y, Pr and Nd) and mixed dopant (La–Y and La–Nd) systems are plotted, the same systems as in Fig. 6 and containing the dopant systems in Fig. 7. The reduction peak temperatures are extracted from the peak in the derivative TG curve of weight with temperature, and the mass loss is calculated as the mass lost between the onset and offset temperature of the peak in the differential TG curve (example in Fig. 2c). Reduction peak temperature and mass lost during reduction plots for materials containing 9 at% and 11 at% La (*y* = 0.09 and *y*= 0.11), 12 at% and 15 at% Y (*y* = 0.12 and *y*= 0.15), and Ce_(1−*y*)/2_Zr_(1−*y*)/2_La_*y*_O_2−*δ*_ materials keeping the Ce/Zr ratio constant at 1 and increasing dopant content (*y*), for single dopant (La, Y, Pr and Nd) and mixed dopant systems (La–Y and La–Nd), are provided in ESI Fig. S23, S24 and S25,† respectively.

During the reduction step, a broad reduction peak in the derivative TG curve of weight with temperature is observed in ceria-zirconia TGA profiles (such as is plotted in Fig. 2c), relating to the bulk and surface Ce^4+^ reduction due to the high mobility of oxygen through the lattice.^31^ The mass lost during reduction is due to the oxygen loss from the reduction of Ce^4+^ to Ce^3+^. The reduction peak temperature is calculated as the temperature of the peak in the derivative TG curve of weight with temperature. The mass lost during reduction is calculated as the mass lost between the onset and offset temperature of the peak in the differential TG curve (example in Fig. 2c). Both quantities were taken from TGA measurements (temperature ramp to 800 °C in 5% H_2_/N_2_) and were used to quantify the reducibility of the materials. The reduction peak temperature and mass loss during reduction are shown in Fig. 8 for undoped, single doped (La, Y, Pr and Nd) and mixed doped (La–Y and La–Nd) materials with the general formula Ce_*x*_Zr_1−*x*−*y*_Ln_*y*_O_2−*δ*_, where the stated lanthanide dopant content (*y*) is fixed and the Ce content (*x*) is varied across Ce contents of 0.00 < *x* ≤ 0.60. The reduction peak temperatures generally decrease with increasing ceria content, and the mass lost during the reduction shows a parabolic trend with ceria content, with a maximum at 0.40 ≤ *x* ≤ 0.50, similar to the relationship observed in OSC with Ce content in literature^14,15^ and with undoped materials in this study (Fig. S7†). The striking similarity between the trend of the mass loss during reduction and OSC with Ce content suggests that the mass loss during reduction could be a proxy for the OSC of the materials. When increasing the dopant content, keeping a constant Ce/Zr ratio of one, the mass loss during reduction was constant at dopant contents below 15 at% but decreased when doping above 15 at% for all single doped (La, Y, Pr and Nd) and mixed dopant (La–Y and La–Nd) systems, which is shown in ESI Fig. S25.† Since the mass loss during reduction and measured OSC show a comparable trend with Ce content, and mass loss during reduction decreases at dopant loadings above 15 at%, there is an optimal dopant content below 15 at% where OSC will be maximised for each dopant system.

Nitrogen adsorption measurements were carried out, spanning the compositional range screened for each dopant system, to give an understanding of the textural properties of the materials. The trends in surface areas, total pore volumes and average pore sizes are provided in ESI Fig. S26–S28,† for materials with the general formula Ce_*x*_Zr_1−*x*−*y*_Ln_*y*_O_2−*δ*_ where the Ce content (*x*) is varied and the stated lanthanide content (*y*) is held constant for single dopant (La, Y, Pr and Nd), mixed dopant (La–Y and La–Nd) and undoped systems. In this compositional space, the surface areas of all dopant systems are higher than undoped and are unchanged across the Ce content range screened of 0.10 < *x* < 0.60. Total pore volumes and pore sizes increase with Ce content for all systems, with doped materials demonstrating comparable total pore volumes and smaller pore sizes than undoped materials. For materials with the general formula Ce_(1−*y*)/2_Zr_(1−*y*)/2_Ln_*y*_O_2−*δ*_, where the Ce/Zr ratio is held constant at 1 and the stated lanthanide content (*y*) is increased (Fig. S29†), for single doped (La, Y, Pr and Nd) and mixed dopant (La–Y and La–Nd) systems, the surface areas increase for each dopant system up to *y*= 0.15 and then decrease significantly at dopant contents above *y*= 0.15, being higher than undoped Ce_0.5_Zr_0.5_O_2−*δ*_ at loadings up to *y*= 0.15. Total pore volumes and pore sizes decrease with dopant content, below that of undoped Ce_0.5_Zr_0.5_O_2−*δ*_, for all dopant contents (0.00 < *y* < 0.25) for all dopant systems.

### Oxygen storage prediction modelling

A set of 30 compositions were chosen for OSC measurements, forming a training set for model development, encompassing undoped, single, and mixed dopant systems (La, Y, Pr, Nd, La–Y and La–Nd). A full list of the chosen compositions is provided in ESI Table S4.† The compositions of the samples were chosen such that one of Ce/Zr ratio, Ce content, dopant system or dopant content was varied while the others were held constant for pairs within the set. An example pair is Ce_0.267_Zr_0.623_Pr_0.110_O_2−*δ*_ and Ce_0.267_Zr_0.623_Nd_0.110_O_2−*δ*_, selected to represent the difference between Pr and Nd at a low Ce/Zr ratio. The 30 compositions were selected to also contain a range in the extracted quantities from the characterisation measurements such as Williamson–Hall crystallite size, strain and mass lost during reduction, which is important in allowing the extracted quantities to be fully evaluated and compared for significance towards OSC prediction, identifying which of the variables alone, or in combination, are the most influential. The 30 compositions that were chosen for OSC measurements contained a Ce content range of 10 at% to 90 at%, a total dopant content range of 0 at% to 22 at%, Williamson–Hall crystallite sizes of the fresh samples varying from 4 nm to 40 nm, a range in reduction peak temperatures from 560 °C to 630 °C and a range in the mass lost during reduction from 0.26 wt% to 1.77 wt%. The final selected compositions for OSC measurements were synthesised at a larger scale using the robotic-based co-precipitation pathway in a scaled-out parallelised approach.

**Fig. 9:**
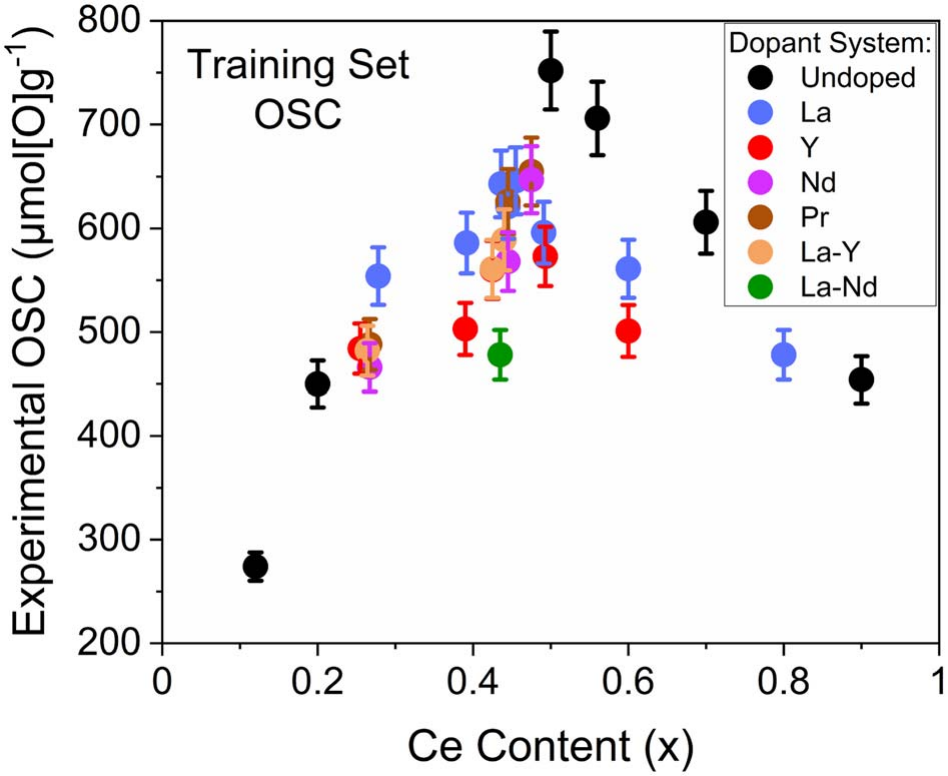
The measured OSC is given against Ce content (*x*) for the 30 samples selected for training the OSC prediction models. The samples contain both undoped and rare-earth dopant systems with the general formula Ce_*x*_Zr_1−*x*−*y*_Ln_*y*_O_2−*δ*_ (where Ln = La, Y, Pr, Nd for single dopant systems or Ln = La–Y and La–Nd for 2 dopant systems), and the full compositions are provided in ESI Table S4.†

The measured OSC of the 30 selected compositions for the development of an OSC prediction model are given in Fig. 9 against Ce content, where a parabolic trend is observed. To connect the measured OSC to the fast-to-measure extracted quantities from the screening techniques, multiple linear regression models were produced through a stepwise approach (described in ESI Section A†), that investigated the use of these quantities as parameters in these models. The variables extracted from the PXRD measurements that were subsequently investigated for incorporation within OSC prediction models were the Scherrer and Williamson–Hall crystallite sizes, Williamson–Hall strains, *Fm*3̄*m* equivalent lattice parameters and the PXRD peak position of the peak at 29° (the (111) peak for pseudo-cubic materials and the (011) peak for tetragonal materials). The variables extracted from the Raman measurements that were investigated for incorporation within OSC prediction models were the F_2g_ peak position and the ratio of the peak intensities for the peaks at around 600 cm^−1^ and 460 cm^−1^ (*I*_D_/*I*_F2g_), pertaining to the stretching vibration of the M^3+^−O−Ce^4+^ ions near an oxygen defect and to the breathing vibration of the Ce^4+^−O−Ce^4+^ bond, respectively, with the ratio relating to the oxygen vacancy concentration in the material with respect to the amount of Ce.^25,32^ The mass loss during reduction and the reduction peak temperatures were also extracted for the OSC prediction model development process, measured from TGA, as well as compositional information (Ce content, Ce/Zr ratio and total dopant content). To not restrict predictions, categorical variables such as the dopant system present were not taken into consideration as this would limit the predictive ability of a model to only dopant systems within the training set. Surface areas, pore volumes and pore sizes from nitrogen adsorption measurements were measured for every sample that was measured for OSC, and the incorporation of these variables within OSC prediction models was investigated along with the aforementioned variables from PXRD, Raman, TGA measurements and composition, in the stepwise prediction model development process (described in ESI Section A†). The investigation of variables from nitrogen adsorption measurements with those variables from high-throughput compatible techniques leads to a more informed and insightful OSC prediction model development process by investigating the correlation of quantities to the porosity as well as investigating the incorporation of the porosity data itself within the models. If information from the nitrogen adsorption measurements are important to OSC, as has been suggested in literature,^33^ and can be captured through fast-to-measure quantities, a more robust OSC prediction model would be obtained.

**Fig. 10:**
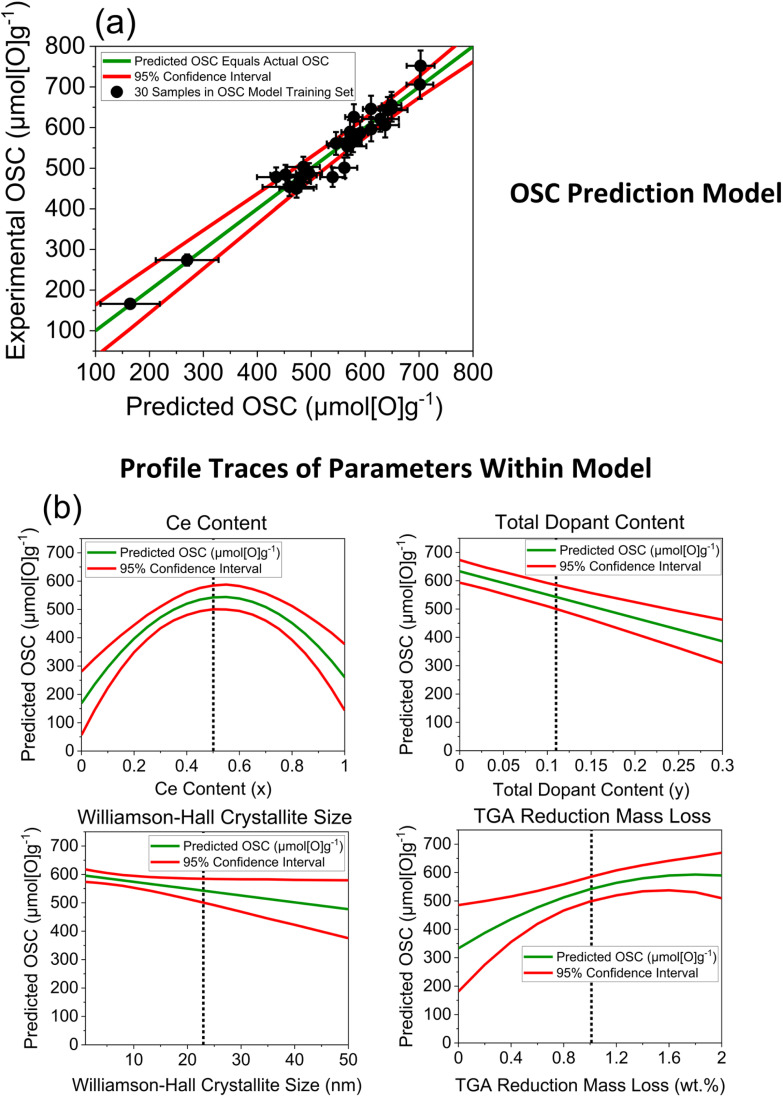
(a) Experimentally measured OSC for the 30 ceria-zirconia materials within the OSC training set (listed in Table S4†) against predicted OSC from a multiple linear regression model that contains the following parameters: Ce content, total dopant content, Williamson–Hall crystallite size and mass loss during reduction. The general formula of the compositions within the training set is Ce_*x*_Zr_1−*x*−*y*_Ln_*y*_O_2−*δ*_ (where Ln = La, Y, Pr, Nd for 1 dopant systems and Ln = La–Y and La–Nd for 2 dopant systems). The red lines give the 95% confidence interval of the predictions. (b) Profile traces of the parameters within the model (green line), giving the predicted OSC value as the parameter is changed over the full range of the *x*-axis, while the other parameters are held constant at values indicated by a dotted line in the respective plots. The 95% confidence intervals are also given as red lines. The full statistical output for this model is provided in ESI Section D2.†

After investigating the incorporation of each of the variables within an OSC prediction model, in a stepwise approach, the optimal multiple linear regression OSC prediction model incorporated a combination of 4 variables and is shown in Fig. 10, with the predicted against actual OSC plot and the profile traces of each of the parameters within the model. Cross-validation was performed by splitting the original 30 sample training set into a new randomly selected training set and test set in an 80/20 split, training the model on the smaller training set using the same combination of parameters as in the original model and testing on the smaller test set. The cross-validation process was repeated for 10 iterations, each time randomly selecting a new training set and test set in an 80/20 ratio. The predicted against experimental OSC plots for each iteration is given in ESI Section D2,† along with the other output statistics from the prediction model, where the predicted values show a strong agreement with the measured OSC, with small residuals for each iteration.

The 4 variables incorporated within the model, selected in combination as being the most significant towards OSC prediction, are the Ce and total dopant content from the composition, the mass loss during reduction from TGA measurements and the Williamson–Hall crystallite size from the PXRD measurements. Therefore, a confident value for the oxygen storage capacity of ceria-zirconia materials can be predicted by taking only compositional information and data from fast-to-measure techniques into consideration, which can complement slower but more precise conventional OSC measurements, for example in screening large libraries of samples to prioritise OSC measurements. The model incorporates the expected parabolic trend of OSC with Ce content, as seen in Fig. 9 and S7,† through a combination of the compositional ceria content and the mass lost during the reduction step variables, as quadratic parameters. The total dopant content and Williamson–Hall crystallite size are also required to capture variance in the OSC predictions, as linear parameters, effectively shifting the OSC predictions higher or lower across the parabolic function. The use of only continuous variables within the model allows maximum flexibility in predictions where each variable is independent of the nature of the dopant system.

The incorporation of the Ce content variable within the OSC prediction model can be explained through the parabolic relationship between OSC and Ce content, which was observed in Fig. 9 and S7.† Ce content was incorporated within the OSC prediction model whereas PXRD peak position and lattice parameter variables were not, due to the three variables showing a similar relationship to OSC and correlating strongly with each other, with all correlation coefficients being greater than 0.9 (the plotted correlations and the correlation coefficients are given in ESI Section D5†). These strong correlations mean that only one of the three variables can be selected without adding noise into the model, since all the variables account for the same variance in measured OSC. A detailed comparison of the models containing Ce content, PXRD peak position and lattice parameter variables is given in the ESI Section D3.† When the incorporation of the Ce content, peak position and lattice parameter variables are compared to each other within the OSC prediction models, keeping the other dopant content, reduction mass loss and Williamson–Hall crystallite size variables included (if they demonstrate statistical significance), inclusion of Ce content is the optimal statistical choice. The model incorporating Ce content has a *p*-value of <0.0001 (Table S7†) for the quadratic Ce content parameter, giving the probability that the parameter estimate is zero for the quadratic Ce content parameter, showing an extremely high significance. When the peak position variable is incorporated within the model in place of the Ce content variable, a *p*-value of 0.098 (Table S10†) is observed for the quadratic peak position parameter, indicating a weak significance. When the lattice parameter variable is incorporated within the model in place of the Ce content variable, a *p*-value of 0.6635 (Table S13†) is observed for the quadratic parameter of the lattice parameter variable, indicating insignificance. Since the Ce content parameter has a *p*-value of <0.0001, indicating the highest significance of the three similar parameters, it is selected in the final model.

It was identified that crystallite size incorporates information on the dopant system after evaluating the extracted crystallite sizes from the screenings (Fig. 7). The inclusion of the crystallite size variable within the model therefore removes the need for the addition of a categorical dopant system variable, which would restrict predictions to only the dopant systems of the samples the model was trained on. Crystallite size also shows a negative correlation with surface area, possessing a moderately strong correlation coefficient of −0.48 (Fig. S34†), which is an important observation for its inclusion within the OSC prediction model, since surface area has previously been shown to be important to OSC in ceria-zirconia material systems.^33^ Crystallite size measurements can thus act as a proxy for surface area in OSC prediction. It was further observed that the data directly collected from nitrogen adsorption measurements were the least significant when incorporated alongside the other variables from PXRD, Raman, TGA and composition (ESI Section D4†), always being omitted when included during the initiation of the stepwise model development process. This either means that the porosity of the materials is not significant for oxygen storage or that, most likely, the impact of porosity on the OSC is already captured by the Williamson–Hall crystallite size. The omission of porosity-based variables within the model allows for a more efficient screening and discovery of high OSC materials since nitrogen adsorption measurements are slower than PXRD and TGA.

As part of the model building process, the inclusion of the Williamson–Hall crystallite size variable was compared with the Scherrer crystallite size variable within OSC prediction models, to examine which of the variables result in a statistically better model after incorporation, similar to the comparison of the inclusion of Ce content, PXRD peak position and lattice parameter variables, with strong correlations between each pair. Table 2 compares the statistical significance of the parameters within the OSC model expression when containing either the Williamson–Hall crystallite size or Scherrer crystallite variable, along with dopant content, Ce content and TGA mass loss variables. The *p*-values in Table 2 associated with each parameter in the models are provided, giving the probability that the inclusion of the parameter in the model expression has no effect on the response, where any parameters with *p*-values above 0.10 are identified as showing insignificance when in combination with the other parameters. The model containing the Williamson–Hall crystallite size shows significance for each of the parameters in the model, whereas the model containing the Scherrer crystallite size does not. The parameter associated with the Scherrer crystallite size variable within the model expression has an associated *p*-value of 0.1779, meaning that its inclusion does not improve the model and would be removed as part of the stepwise model development process (described in ESI Section A4†).

**Table 2:** A comparison of the statistical significance of each parameter within the prediction expression of OSC prediction models, where either the Williamson–Hall or the Scherrer crystallite size variables are incorporated. Both models also contain dopant content, Ce content and TGA mass loss variables. The prediction expressions of each model contain a combination of linear and quadratic parameters. The *p*-values associated with each parameter in the models are provided, giving the probability that the inclusion of the parameter in the model expression has no effect on the response, in this case being the OSC of the materials. A *p*-value greater than 0.10 is identified as the parameter showing insignificance when in combination with the other parameters. The full statistical output of the model containing Williamson–Hall crystallite size (Fig. 10) is given in ESI Section D2,† and the model containing the Scherrer crystallite size is given in ESI Section D7†

Model parameter	*p*-Values with W–H crystallite size	*p*-Values with Scherrer crystallite size
Total dopant content (linear term)	<0.0001	<0.0001
Ce content (linear term)	<0.0001	<0.0001
TGA reduction mass loss (linear term)	0.0007	0.0020
Ce content (quadratic term)	<0.0001	<0.0001
TGA reduction mass loss (quadratic term)	0.0799	0.0979
Williamson–Hall crystallite size (linear term)	0.0394	
Scherrer crystallite size (linear term)		0.1779

Since the incorporation of the Williamson–Hall crystallite size variable, alongside dopant content, Ce content and TGA reduction mass loss variables, gave a statistically better prediction model than that incorporating the Scherrer crystallite size, it suggests that the assumptions within the Williamson–Hall method are valid. This indicates that by removing the contribution of lattice strain to the peak broadening in the PXRD, a more physically meaningful crystallite size can be calculated than is possible from the Scherrer method, leading to a superior predictive model. The incorporation of the Williamson–Hall within the OSC prediction model requires the Raman measurements for the crystal structure identification, ensuring that the correct number and position of peaks are fit in the PXRD patterns to extract crystallite sizes.

The final variable utilised in the statistically optimal OSC prediction model (Fig. 10) is the total dopant content, which demonstrates an overall negative correlation with OSC for the doped samples within the training set when extrapolated up to 22 at%, giving a correlation coefficient of −0.49, showing a moderate correlation (ESI Fig. S37†). However, below a dopant content of 15 at%, the reduction mass loss was constant or increased depending on the dopant system present, relating to an improved or optimal OSC that was in many cases larger than for undoped materials (Fig. S23b and S25b†). Since the mass loss during reduction is constant or increases at lower dopant contents below 15 at%, correlating to constant or increasing OSC, but decreases above dopant amounts of 15 at%, it can be fit with a linear function possessing a negative gradient when extrapolated to dopant contents up to 22 at%, that is contained within the OSC model training set (ESI Table S4†).

Simpler models incorporating only PXRD or TGA information were also investigated, which if successful would contribute to a more efficient OSC prediction workflow. The statistical outputs from both models are provided in the ESI Sections D10 to D13,† however, both simple models were statistically inferior to the OSC prediction model combining variables from different techniques and compositional information. Experimental OSC against predicted OSC plots for the optimal OSC prediction model (Fig. 10) and the PXRD-only and TGA-only prediction models are provided in ESI Fig. S41,† where the model incorporating variables from multiple techniques gives much improved predictions.

**Fig. 11:**
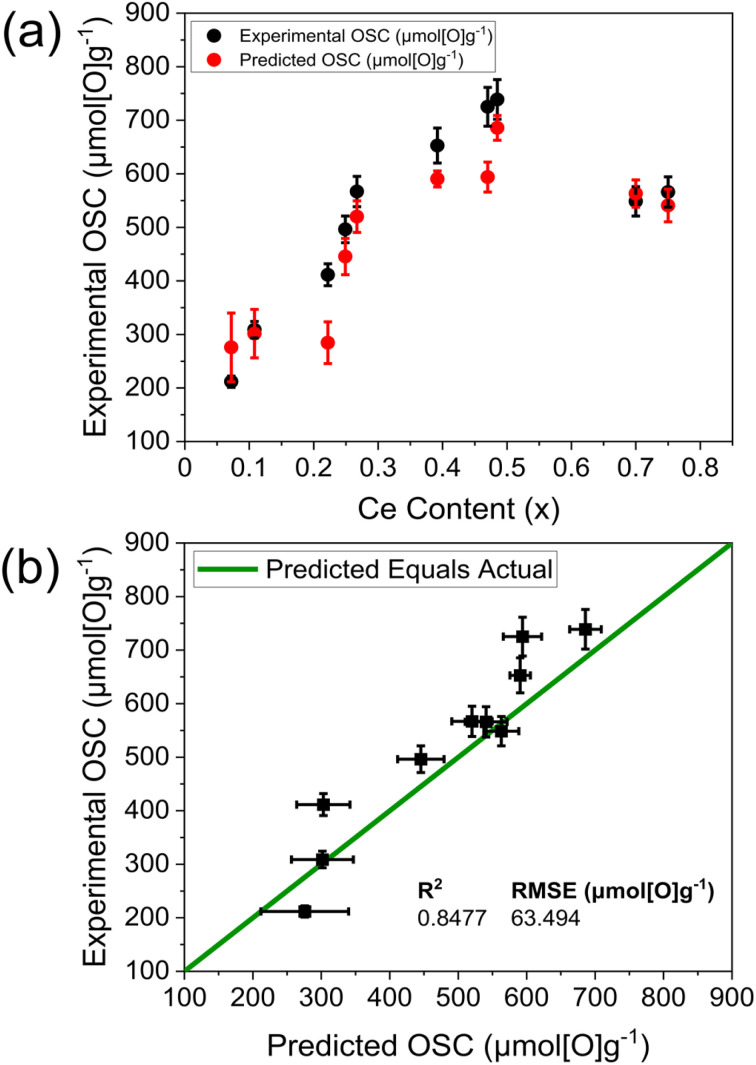
(a) Variation of experimentally measured OSC (black) and predicted OSC (red) against Ce content for OSC predictions from the optimal OSC prediction model given in Fig. 10, containing dopant content, Ce content, TGA reduction mass loss and Williamson–Hall crystallite size variables, for the compositions within the 10-sample validation set. The compositions are provided in ESI Table S26† and have the general formula Ce_*x*_Zr_1−*x*−*y*_Ln_*y*_O_2−*δ*_, where Ln = La, Y, Pr, Nd for single dopant systems or Ln = La–Y and La–Nd for 2 dopant systems. (b) Experimental OSC values are plotted against the predicted OSC values from the OSC prediction model given in Fig. 10 (same as in (a)), along with the *R*^2^ and root mean square error (RMSE) values.

To validate the optimal OSC prediction model in Fig. 10, containing dopant content, Ce content, TGA mass loss and Williamson–Hall crystallite size variables, an independent set of 10 samples was selected for OSC measurements (listed in ESI Table S26†), to probe the extremes of where the model was trained, covering a range of predicted OSC from 200 μmol g^−1^ to 700 μmol g^−1^, with a larger proportion of mixed dopant compositions in the validation set (60%, ESI Table S26†) than in the training set (13%, ESI Table S4†). Fig. 11 shows the experimental OSC obtained from the samples within the validation set and the corresponding predicted OSC from the OSC prediction model (Fig. 10). The predicted OSC values show a strong agreement with the experimentally measured values, correctly predicting the overall trend in OSC across the materials as well as the absolute values of OSC, with a high *R*^2^ and a low root mean square error (RMSE). The plot in Fig. 11b shows that the majority of the variance in OSC is covered by the predictions; where the *R*^2^ value of 0.8477 indicates 84.77% of the variance is covered and the RMSE is the standard deviation of the residuals, at 63.494 μmol[O]g^−1^.^34^ The model correctly identified the materials possessing the largest and smallest OSC from the library screenings within error, being Ce_0.485_Zr_0.485_Nd_0.030_O_2−*δ*_ with an experimental OSC of 739(37) μmol g^−1^ and a predicted OSC of 686(23) μmol g^−1^, and Ce_0.072_Zr_0.828_La_0.05_Nd_0.05_O_2−*δ*_ with an experimental OSC of 212(11) μmol g^−1^ and a predicted OSC of 276(64) μmol g^−1^. The Ce_0.485_Zr_0.485_Nd_0.030_O_2−*δ*_ material was predicted to have an OSC of 686(23) μmol g^−1^ which is larger than the largest OSC of any doped material in the training set, being Ce_0.475_Zr_0.475_Nd_0.05_O_2−*δ*_ with an OSC of 655(33) μmol g^−1^. The measured OSC for the Ce_0.485_Zr_0.485_Nd_0.030_O_2−*δ*_ material was 739(37) μmol g^−1^ which demonstrates the ability of the model to correctly predict materials that outperform the training set. The compositions with the largest predicted to experimental OSC differences are Ce_0.470_Zr_0.470_La_0.05_Y_0.01_O_2−*δ*_ and Ce_0.222_Zr_0.518_La_0.260_O_2−*δ*_, with 18% and 31% differences, respectively. A reason for this is that Ce_0.470_Zr_0.470_La_0.05_Y_0.01_O_2−*δ*_ is a low dopant content mixed dopant material, with a lower dopant content than any mixed dopant material in the training set, the lowest being Ce_0.440_Zr_0.440_La_0.090_Y_0.030_O_2−*δ*_ with 12 dopant at%. Similarly, Ce_0.222_Zr_0.518_La_0.260_O_2−*δ*_ possesses a larger dopant content than any doped material in the training set, with the highest being Ce_0.390_Zr_0.390_Y_0.220_O_2−*δ*_, possessing 22 dopant at% (the full list of the compositions in the training set and validation set are given in ESI Tables S4 and S26,† respectively).

In order to further demonstrate the ability of the model to predict materials which outperform the training set, the two highest OSC materials in the training set were removed and added to the validation set (the training set is shown in ESI Fig. S42b†). Encouragingly, the model gave OSC predictions for the two compositions which were removed from the training set that were larger than the largest OSC in the revised training set, correctly predicting that these two materials outperform all the samples in the revised training set (the predicted against actual OSC plots are given in ESI Fig. S44† and the model and output statistics are given in ESI Fig. S43† and Tables S27 to S29†).

The generalisability of the model was demonstrated by removing all the materials containing La from the training set, leaving only undoped, Y-doped, Pr-doped, and Nd-doped compositions (the training set is shown in ESI Fig. S45b†). The model was then retrained on the reduced training set and validated on the La containing materials that were removed from the training set in combination with the La containing materials in the original validation set (the model is given in ESI Fig. S46† and the output statistics are given in Tables S30 to S32†). The OSC of the compositions containing the unseen La dopant were predicted well, with the model correctly selecting the highest and lowest OSC materials (the predicted against actual OSC plots are given in Fig. S47†). These results highlight the generalisability of the model to predict the OSC of materials containing an unseen lanthanide dopant, in both single and mixed dopant compositions.

Since the OSC prediction model developed in this study gives predictions matching experimental observations, generalising well to composition types that are not present in the training data, and requiring only fast-to-measure variables, the OSC of new materials can be predicted faster than by conventional reactor-based OSC measurements. The model is not only faster than conventional OSC measurements, but also can run continuously sequentially through each sample, *via* automated high-throughput PXRD, Raman and TGA measurements. The overall trend in OSC across the independent validation set being predicted well by the model provides confidence in employing the model across the entire set of screened materials (given in ESI Fig. S48†) and future libraries of materials to accelerate the discovery of stable, high oxygen storage capacity ceria-zirconia materials.

Although the materials in this study are screened with fixed synthetic parameters, any synthetic parameters expected to impact performance of materials could be used in a proxy model, such as pH of the precipitation and the temperature of calcination in the case of ceria-zirconia, or the order of addition of elements, which impacts Fischer–Tropsch catalyst performance.^23^ In practice, the iterative model development process can be utilised for systems possessing a narrow sweet spot for high performance through the careful design of training and validation sets, updating the model after each set of experiments.

Development of an OSC prediction model for a different materials system would require identification of proxies that reflected the chemistry of that system. For example, if an amorphous phase contributed to the catalyst performance of a given system, the characterisation metrics (such as information from Raman spectroscopy or TGA) used in the proxy model would need to be connected to that phase. The models in this study are not a replacement for OSC performance measurements but rather a tool for the optimal selection of materials for the slow OSC measurements using fast-to-measure high-throughput techniques.

## Conclusion

Multiple libraries of rare-earth doped ceria-zirconia materials were synthesised using a robotic-based high-throughput co-precipitation pathway. The materials produced were subsequently screened with automated high-throughput PXRD, Raman and TGA to extract information relating to structure, thermal stability and reducibility. Thermally stable materials were identified by PXRD after a high temperature ageing treatment as those which retained a single phase. Raman allowed exact phase identification and TGA probed the temperature of reduction and the mass lost during reduction. A data set was constructed from the information extracted *via* the automated PXRD, Raman and TGA measurements, allowing the development of a prediction model for the oxygen storage capacity (OSC) of thermally stable rare-earth doped ceria-zirconia materials. The model was built by quantifying the connection between the extracted metrics from the fast-to-measure high-throughput analytical techniques with the OSC measured on a set of samples selected to span the range of compositions and metrics. Such predictive models can be built from data generated *via* approaches that are non-automated or low throughput, albeit over a longer time frame and at a smaller ultimate scale. Robotic instruments were utilised in this study to accelerate the materials discovery process but are not essential for the co-precipitation synthesis.

The statistically optimal OSC prediction model produced incorporates the Williamson–Hall crystallite size from the PXRD measurements, the mass loss during reduction from the TGA, and compositional Ce and total dopant content of the fresh materials. This makes the predictions independent of the chemical identity of the dopant and generalises well to composition types that are not present in the training data. This model successfully predicted the trend in OSC measured for an independent set of materials, distinct from those used to train the model, including identification of those samples with the largest and smallest OSC. The model only uses metrics that can be acquired rapidly, enabling the accelerated assessment of the OSC of a material, for example to prioritise samples for the necessarily slower OSC measurements.

The success of the approach in this study motivates the development of proxy models for other catalyst chemistries beyond lanthanide-substituted ceria-zirconia through the development of an appropriate high-throughput synthetic protocol, automated characterisation data collection, and analysis protocols which reflect the specific chemistry involved.

## Data availability

The data collected for this work is available *via* the University of Liverpool data repository at https://datacat.liverpool.ac.uk/id/eprint/2474.

## Author contributions

Jack J. Quayle: data curation, formal analysis, investigation, methodology, visualization, software, writing – original draft, review & editing; Alexandros P. Katsoulidis: conceptualization, supervision, writing – review & editing; John B. Claridge: conceptualization, supervision, writing – review & editing; Andrew P.E. York: conceptualization, supervision, resources, writing – review & editing; David Thompsett: conceptualization, supervision, resources, writing – review & editing; Matthew J. Rosseinsky: conceptualization, supervision, writing – review & editing.

## Conflicts of interest

There are no conflicts to declare.

## Supplementary Material

SC-014-D3SC03558A-s001
