# Supplementary material for: A proxy for oxygen storage capacity from high-throughput screening and automated data analysis
Source: Chem Sci. 2023 Oct 23;14(44):12621–36. doi: 10.1039/d3sc03558a (PMC10646963; doi:10.1039/d3sc03558a)
Supplement: SC-014-D3SC03558A-s001 [file SC-014-D3SC03558A-s001.pdf]

Electronic Supplementary Material (ESI) for Chemical Science.
This journal is © The Royal Society of Chemistry 2023

Supplementary Information

A Proxy for Oxygen Storage Capacity from High-throughput Screening and Automated Data Analysis

Jack J. Quayle [a], Alexandros P. Katsoulidis [a], John B. Claridge [a], Andrew P.E. York [b], David Thompsett [b], Matthew J. Rosseinsky [a]

[a] Department of Chemistry, University of Liverpool, Crown Street, Liverpool L69 7ZD, UK

[b] Johnson Matthey Technology Centre, Blounts Court Road, Reading RG4 9NH, UK

Table of Contents

A. Experimental 3
A.1. Catalyst Preparation 3
A.2. Characterization Techniques 3
A.3. Automated Analysis 5
A.3.1. PXRD Analysis 5
A.3.2. Raman Analysis 8
A.3.3. TGA Analysis 11
A.4. Prediction Modelling 13
A.4.1. Stepwise Model Development 13
A.4.2. Statistical Output 13
B. OSC Measurements of Undoped Robotic Samples Compared to References 14
C. Materials Screening 15
C.1. Compositions Screened 15
C.1.1. Varying Ce Content with Constant Dopant Content 15
C.1.2. Increasing Dopant Content with a Constant Ce/Zr Ratio of 1 15
C.2. PXRD Patterns 16
C.2.1. Varying Ce/Zr Ratio with Constant Dopant Content 16
C.2.2. Increasing Dopant Content with a Constant Ce/Zr Ratio of 1 18
C.3. Raman Spectra 19
C.3.1. Varying Ce/Zr Ratio with Constant Dopant Content 19
C.3.2. Increasing Dopant Content with a Constant Ce/Zr Ratio of 1 21
C.4. Lattice Parameters 22
C.4.1. Varying Ce/Zr Ratio with Constant Dopant Content 22
C.4.2. Increasing Dopant Content with a Constant Ce/Zr Ratio of 1 24

C.5. ICP Analysis 25
C.6. SEM-EDS Analysis 25
C.7. Williamson-Hall Analyses 26
C.7.1. Varying Ce/Zr Ratio with Constant Dopant Content 26
C.7.2. Increasing Dopant Content with a Constant Ce/Zr Ratio of 1 29
C.8. TGA Reduction 30
C.8.1. Varying Ce/Zr Ratio with Constant Dopant Content 30
C.8.2. Increasing Dopant Content with a Constant Ce/Zr Ratio of 1 32
C.9. Nitrogen Adsorption 33
C.9.1. Varying Ce/Zr Ratio with Constant Dopant Content 33
C.9.2. Increasing Dopant Content with a Constant Ce/Zr Ratio of 1 34
D. Prediction Modelling 35
D.1. 30-sample Training Set 35
D.2. Best OSC Prediction Model Statistical Output and Cross-validation 36
D.3. OSC Prediction Models Containing PXRD Peak Position and Lattice Parameter Variables Over Ce Content 38
D.4. Models Containing Nitrogen Adsorption Variables 42
D.5. Ce Content, PXRD Peak Position and Lattice Parameter Correlations 44
D.6. Williamson-Hall Crystallite Size Correlation to Surface Area 45
D.7. Scherrer Crystallite Size Inclusion Within OSC Prediction Model 45
D.8. Williamson-Hall Crystallite Size Correlation to Scherrer Crystallite Size 48
D.9. Total Dopant Content Correlation to OSC 48
D.10. PXRD-only and TGA-only OSC Prediction Models 49
D.11. PXRD-only Model Statistical Output 50
D.12. TGA-only Model Statistical Output 52
D.13. Validation of OSC Prediction Models 53
D.14. Model after Removing the Two Highest OSC Materials from the Training Set 54
D.15. Model after Removing La containing OSC Materials from the Training Set 57
D.16. Predicted OSC of Screened Compositions 61
E. References 61

A. Experimental

A.1. Catalyst Preparation

CeO_2-ZrO_2-RE_2O_3 (RE = La, Y, Pr, Nd) and CeO_2-ZrO_2-La_2O_3-RE_2O_3 (RE = Y, Nd) materials were prepared by a co-precipitation method, utilising the Gilson GX-281 Liquid Handler. This pathway was adapted from different routes within the literature[1-3], for compatibility with a high-throughput robotic-based system. Initially, the required amounts of $Ce(NO_3)_3{\cdot}6H_2O$, $ZrO(NO_3)_2{\cdot}6H_2O$, and $RE(NO_3)_3{\cdot}6H_2O$ (RE = La, Y, Pr, Nd) were dissolved in ultrapure water and combined in the required amounts by the Gilson GX-281 Liquid Handler. The aqueous solutions were precipitated with 14.7 M NH_4OH to give a pH of 10 after precipitation. The precipitate was then shaken for 24 h by an orbital shaker with the liquid handler, before being obtained via centrifugation with washings (with ultrapure water). The samples were then dried at 100 °C for 16 h in air, within the centrifuge tubes. The dried samples were ground and calcined at 600 °C for 4 h in air, within a ceramic well plate, to obtain the oxide (approximately 150 mg produced per sample). A portion of each sample was subjected to thermal ageing with a second calcination step at 1100 °C for 4 h in air, where thermally stable materials retained a single-phase after ageing.

A scaled-out approach was used to obtain larger amounts of each sample for OSC measurements (600-800 mg, 4-5 times the scale used for screening), synthesising multiples of the same composition in parallel using the above methodology, before combining after calcination.

A.2. Characterization Techniques

The powder X-ray diffraction (PXRD) experiments were recorded on a Bruker diffractometer using monochromatic Cu Kα radiation (λ = 0.15406 nm). The range of the scan was $10 \leq 2\theta$ (°) ≤ 100 with a step size of 0.01 °. The X-ray tube was operated at an accelerating voltage of 40 kV and an emission current of 40 mA. Automated PXRD measurements were carried out using a robotic arm to automatically load, run and unload samples sequentially through a series of sample.

Laser Raman spectra were recorded using an inVia Reflex Qontor Confocal Raman Microscope with a 532 nm excitation wavelength laser. The spectral acquisition consisted of 1 accumulation of 10 s for each sample. The measured frequency range was 100 cm^{-1} to 3000 cm^{-1}. The high-throughput measurements utilised a 384-well plate and microplate mapping to automatically analyse each sample in a sequential route by focussing the microscope just above the bottom of each well, outputting spectra into a single text file.

Thermogravimetric analysis (TGA) measurements were collected on a Q5000IR instrument. Approximately 5-15 mg of sample was loaded onto each platinum pan. The method ran on each sample, in an automated sequential way, was to flow 5% H_2/N_2 (50 $mLmin^{-1}$) at room temperature for 30 minutes before ramping to 800 °C under a 5% H_2/N_2 (50 $mLmin^{-1}$) flow, with a heating rate of 10 $°Cmin^{-1}$.

Surface areas, pore volumes and pore-size distributions of the materials were measured by nitrogen adsorption-desorption at 77 K using a Micromeritics Tristar II. Prior to adsorption measurements, samples were degassed at 300 °C for 3 h under vacuum. The Brunauer-Emmett-Teller (BET) and

Barrett-Joyner-Halenda (BJH) methods were applied to calculate the surface areas and pore size distributions (using the adsorption branch), respectively.

Scanning electron microscopy (SEM) was utilised for imaging and composition determination using a Hitachi S4800 instrument. Prior to measurement, the samples were placed on a carbon tab and sputter-coated with carbon. Acceleration voltages of 10 kV and 20 kV were used to capture the images. Energy Dispersive X-ray Spectroscopy (EDS) was utilized to determine sample composition and homogeneity. SEM-EDS results were obtained at acceleration voltage of 20 kV and an acceleration current of 20 µA with a working distance of 15.0 mm.

The metal composition of the supernatant removed after centrifugation was measured using an Agilent 5110 ICP-OES instrument. The supernatant was measured without further pre-treatment. Ce and Zr elements were analysed with all analyses performed in quadruplicate.

Oxygen storage capacity (OSC) measurements were carried out on 50 mg of pelletised catalyst (250-355 µm). The sample was loaded into a quartz tube, in-between quartz wool, and placed into a reactor. The exhaust gases were monitored via a mass spectrometer. The samples were pre-treated for up to 1 h at room temperature, switching between oxidising (5% O_2/He at 50 mLmin^{-1}) and reducing (100% CO at 50 mLmin^{-1}) conditions every 60 seconds, along with a tracer gas (Ar at 10 mLmin^{-1}) and carrier gas (He at 40 mLmin^{-1}) flow, until the system was stable with respect to the mass spectrometer readings. The OSC was recorded at 500 °C by flowing 5% O_2/He at 50 mLmin^{-1} for 300 seconds before flowing 100% CO at 50 mLmin^{-1} for 300 seconds and recording the CO_2 produced, multiple times, all while flowing a tracer gas (Ar at 10 mLmin^{-1}) and carrier gas (He at 40 mLmin^{-1}) along with the oxidising or reducing gas flows. The OSC was determined by integrating the total area under the CO_2 curve (peak in the mass spectrometer reading) for the length of the reducing cycle (300 seconds).

A.3. Automated Analysis

A.3.1. PXRD Analysis

The analysis of PXRD patterns was carried out using TOPAS-Academic V5 software, by using jEdit 4.3.1 text editor with Topas, creating a command file (.bat file) to run the TOPAS-Academic V5 software from the command line. This ran analyses automatically sequentially for each sample.

The lattice parameters were extracted assuming a $Fm\bar{3}m$ space group for the cubic and pseudo-cubic materials, and a $P4_2/nmc$ space group for the tetragonal materials. When fitting the patterns, a pseudo-voigt peak shape was employed and a Chebyshev background was used for both analyses. The specific input file used in this work for lattice parameter determination is given in Figure S1, for both $Fm\bar{3}m$ (Figure S1a) and $P4_2/nmc$ crystal structures (Figure S1b). In both cases, the analyses resulted in a text file containing the lattice parameters and errors, as well as the fits and raw PXRD patterns for each sample being extracted to text files.

(a)

```
r_wp 0 r_exp 0 r_p 0 r_wp_dash 0 r_p_dash 0 r_exp_dash 0 weighted_Durbin_Watson
0 gof 0

iters 1000000 'Maximum number of iterations of refinement
chi2_convergence_criteria 0.001 'Stop criteria for refinement
do_errors 'Reports errors for each refined value

#define topas_old_version 'include this if using v4 or v5

xdd filename
  start_X 10
  finish_X 100

'=== BACKGROUND FUNCTION ===
bkg @ 0 0 0 0 0 0
      0 0 0 0 0 0
One_on_X(@, 1000) 'Refines a 1/x background function ideal for describing low
angle background

'=== RADIATION SOURCE - Information on New Bruker Cu Ka1 D8 ===
lam
  ymin_on_ymax 0.0001
  la 1 lo 1.540596 lh 0.2
  LP_Factor(!th2_monochromator, 27.3) 'Lorentz-Polarisation factor
  Simple_Axial_Model(@,8)
  Zero_Error(@, 0)

'==============================================================================
'6. PAWLEY PHASE INFORMATION - Crystallographic information for Pawley fitting
of phase
'==============================================================================
  hkl_Is
    a lpa_1 5.284
    b =Get(a);
    c =Get(a);
    al 90.000
    be 90.000
    ga 90.000
    volume 147.5
    space_group "Fm-3m"
    'site Ce1    x 0.0000        y 0.0000        z 0.0000        occ
    Ce-10737418240.500
    'site Zr1    x 0.0000        y 0.0000        z 0.0000        occ Zr+0 0.500
    'site O1     x 0.2500        y 0.2500        z 0.2500        occ O+0  0.992
    PV_Peak_Type(@, 0.02,@, 0.02,@, 0.02,@, 0.02,@, 0.02,@, 0.02)

'{{{ Output lines here
#define write_out
#ifdef write_out
out "lp.txt" append
Out_String(filename)
Out(Get (r_wp), " %11.5f")
Out(lpa_1, " %11.5f", " %11.5f\n")

xdd_out ##filename##.xy load out_record out_fmt out_eqn
   {
       " %11.5f  " = X;
       " %11.5f  " = Ycalc;
       " %11.5f\n  " = Yobs;
   }

#endif
'}}}
```

(b)

```
r_wp 0 r_exp 0 r_p 0 r_wp_dash 0 r_p_dash 0 r_exp_dash 0 weighted_Durbin_Watson
0 gof 0

iters 1000000 'Maximum number of iterations of refinement
chi2_convergence_criteria 0.001 'Stop criteria for refinement
do_errors 'Reports errors for each refined value

#define topas_old_version 'include this if using v4 or v5

xdd filename

'=== FILE PREPARATION ===
x_calculation_step = Yobs_dx_at(Xo); 'Sets the calculation step size for
Rietveld refinement.

'=== BACKGROUND FUNCTION ===
bkg @ 0 0 0 0 0 0
      0 0 0 0 0 0
One_on_X(@, 1000) 'Refines a 1/x background function ideal for describing low
angle background

'=== RADIATION SOURCE - Information on New Bruker Cu Ka1 D8 ===
lam
  ymin_on_ymax 0.0001
  la 1 lo 1.540596 lh 0.2
  LP_Factor(!th2_monochromator, 27.3) 'Lorentz-Polarisation factor
  Simple_Axial_Model(@,8)
  Zero_Error(@, 0)

'==============================================================================
'6. PAWLEY PHASE INFORMATION - Crystallographic information for Pawley fitting
of phase
'==============================================================================
  hkl_Is
    a lpa_1 3.7226
    b =Get(a);
    c lpc_1 5.2894
    al 90.
    be 90.
    ga 90.
    volume 73.3
    space_group "P42/nmcS"
    'site Zr1    x 0            y 0            z 0            occ Zr+4 0.5
    beq 0.54
    'site Ce1    x 0            y 0            z 0            occ Ce+4 0.5
    beq 0.54
    'site O1     x 0            y 0.5          z 0.222        occ O-2  1.
    beq 1.29
    PV_Peak_Type(@, 0.02,@, 0.02,@, 0.02,@, 0.02,@, 0.02,@, 0.02)

'{{{ Output lines here
#define write_out
#ifdef write_out
out "lp_t.txt" append
Out_String(filename)
Out(Get (r_wp), " %11.5f")
Out(lpa 1, " %11.5f", " %11.5f")
Out(lpc_1, " %11.5f", " %11.5f\n")

xdd_out ##filename##.xy load out_record out_fmt out_eqn
   {
       " %11.5f  " = X;
       " %11.5f  " = Ycalc;
       " %11.5f\n  " = Yobs;
   }

#endif
'}}}
```

Fig. S1. Input files within jEdit 4.3.1 text editor to extract (a) cubic/pseudo-cubic or (b) tetragonal lattice parameters with errors and r_wp, as well as outputting the fits and raw PXRD patterns as xy files.

Fitting the individual peaks in the PXRD patterns was also carried out. When fitting the individual peaks, a pseudo-voigt peak shape was employed and a Chebyshev background was used. The specific input files used in this work are given in Figure S2 for peak fitting both $Fm\bar{3}m$ (Figure S2a) and $P4_2/nmc$ (Figure S2b) crystal structures. The peak positions, full width half maximum (FWHM), respective errors and r_wp are output to a text file, as well as the fits and raw PXRD patterns as xy files.

(a)

```
r_wp 0 r_exp 0 r_p 0 r_wp_dash 0 r_p_dash 0 r_exp_dash 0 weighted_Durbin_Watson 0 gof 0

iters 1000000 'Maximum number of iterations of refinement
chi2_convergence_criteria 0.001 'Stop criteria for refinement
do_errors 'Reports errors for each refined value

#define topas_old_version 'include this if using v4 or v5

xdd filename
  start_X 10
  finish_X 100

'=== FILE PREPARATION ===
x_calculation_step = Yobs_dx_at(Xo); 'Sets the calculation step size for Rietveld refinement.

'=== BACKGROUND FUNCTION ===
bkg @ 0 0 0 0 0 0
      0 0 0 0 0 0
One_on_X(@, 1000) 'Refines a 1/x background function ideal for describing low angle background

'=== RADIATION SOURCE - Information on New Bruker Cu Ka1 D8 ===
lam
  ymin_on_ymax 0.0001
  la 1 lo 1.540596 lh 0.2
  LP_Factor(!th2_monochromator, 27.3) 'Lorentz-Polarisation factor
  Simple_Axial_Model(@,8)
  Zero_Error(@, 0)

 prm pklor 0.0001 min 0 max 1

xo_Is xo a1  29.17886 I @  60.56582 peak_type pv pv_lor =pklor; pv_fwhm b1  1 min 0.001 max 5
xo_Is xo a2  33.81872 I @  21.79775 peak_type pv pv_lor =pklor; pv_fwhm b2  1 min 0.001 max 5
xo_Is xo a3  48.57758 I @  95.21760 peak_type pv pv_lor =pklor; pv_fwhm b3  1 min 0.001 max 5
xo_Is xo a4  57.67593 I @  115.1515 peak_type pv pv_lor =pklor; pv_fwhm b4  1 min 0.001 max 5
xo_Is xo a5  60.50102 I @  22.15761 peak_type pv pv_lor =pklor; pv_fwhm b5  1 min 0.001 max 5
xo_Is xo a6  71.14279 I @  28.93702 peak_type pv pv_lor =pklor; pv_fwhm b6  1 min 0.001 max 5
xo_Is xo a7  78.67858 I @  72.97865 peak_type pv pv_lor =pklor; pv_fwhm b7  1 min 0.001 max 5
xo_Is xo a8  81.14042 I @  43.57472 peak_type pv pv_lor =pklor; pv_fwhm b8  1 min 0.001 max 5
xo_Is xo a9  90.87000 I @  57.89604 peak_type pv pv_lor =pklor; pv_fwhm b9  1 min 0.001 max 5


'{{{ Output lines here
#define write_out
#ifdef write_out
out "peakpos_fwhm.txt" append
Out_String(filename)
Out(Get (r_wp), " %11.5f\n")
Out(a1, " %11.5f", " %11.5f")
Out(b1, " %11.5f", " %11.5f\n")
Out(a2, " %11.5f", " %11.5f")
Out(b2, " %11.5f", " %11.5f\n")
Out(a3, " %11.5f", " %11.5f")
Out(b3, " %11.5f", " %11.5f\n")
Out(a4, " %11.5f", " %11.5f")
Out(b4, " %11.5f", " %11.5f\n")
Out(a5, " %11.5f", " %11.5f")
Out(b5, " %11.5f", " %11.5f\n")
Out(a6, " %11.5f", " %11.5f")
Out(b6, " %11.5f", " %11.5f\n")
Out(a7, " %11.5f", " %11.5f")
Out(b7, " %11.5f", " %11.5f\n")
Out(a8, " %11.5f", " %11.5f")
Out(b8, " %11.5f", " %11.5f\n")
Out(a9, " %11.5f", " %11.5f")
Out(b9, " %11.5f", " %11.5f\n")

xdd_out ##filename##.xy load out_record out_fmt out_eqn
   {
        " %11.5f  " = X;
        " %11.5f  " = Ycalc;
        " %11.5f\n  " = Yobs;
   }
#endif
'}}}
```

(b)

```
r_wp 0 r_exp 0 r_p 0 r_wp_dash 0 r_p_dash 0 r_exp_dash 0 weighted_Durbin_Watson 0 gof 0

iters 1000000 'Maximum number of iterations of refinement
chi2_convergence_criteria 0.001 'Stop criteria for refinement
do_errors 'Reports errors for each refined value

#define topas_old_version 'include this if using v4 or v5

xdd filename
  start_X 20
  finish_X 70

'=== FILE PREPARATION ===
x_calculation_step = Yobs_dx_at(Xo); 'Sets the calculation step size for Rietveld refinement.

'=== BACKGROUND FUNCTION ===
bkg @ 0 0 0 0 0 0
      0 0 0 0 0 0
One_on_X(@, 1000) 'Refines a 1/x background function ideal for describing low angle background

'=== RADIATION SOURCE - Information on New Bruker Cu Ka1 D8 ===
lam
  ymin_on_ymax 0.0001
  la 1 lo 1.540596 lh 0.2
  LP_Factor(!th2_monochromator, 27.3) 'Lorentz-Polarisation factor
  Simple_Axial_Model(@,8)
  Zero_Error(@, 0)

prm pklor 0.0001 min 0 max 1

xo_Is xo a1  29.5373 I @  1171.87 peak_type pv pv_lor =pklor; pv_fwhm b1  1 min 0.001 max 5
xo_Is xo a2  34.0006 I @  185.6   peak_type pv pv_lor =pklor; pv_fwhm b2  1 min 0.001 max 5
xo_Is xo a3  34.3558 I @  186.43  peak_type pv pv_lor =pklor; pv_fwhm b3  1 min 0.001 max 5
xo_Is xo a4  42.1151 I @  2       peak_type pv pv_lor =pklor; pv_fwhm b4  1 min 0.001 max 5
xo_Is xo a5  49.1132 I @  272.71  peak_type pv pv_lor =pklor; pv_fwhm b5  1 min 0.001 max 5
xo_Is xo a6  49.3766 I @  259.06  peak_type pv pv_lor =pklor; pv_fwhm b6  1 min 0.001 max 5
xo_Is xo a7  52.5306 I @  4       peak_type pv pv_lor =pklor; pv_fwhm b7  1 min 0.001 max 5
xo_Is xo a8  58.125  I @  157.92  peak_type pv pv_lor =pklor; pv_fwhm b8  1 min 0.001 max 5
xo_Is xo a9  58.5937 I @  163.02  peak_type pv pv_lor =pklor; pv_fwhm b9  1 min 0.001 max 5
xo_Is xo a10  61.3082 I @  33      peak_type pv pv_lor =pklor; pv_fwhm b10  1 min 0.001 max 5
xo_Is xo a11  66.8672 I @  1       peak_type pv pv_lor =pklor; pv_fwhm b11  1 min 0.001 max 5

'{{{ Output lines here
#define write_out
#ifdef write_out
out "peakpos_fwhm_t.txt" append
Out_String(filename)
Out(Get (r_wp), " %11.5f\n")
Out(a1, " %11.5f", " %11.5f")
Out(b1, " %11.5f", " %11.5f\n")
Out(a2, " %11.5f", " %11.5f")
Out(b2, " %11.5f", " %11.5f\n")
Out(a3, " %11.5f", " %11.5f")
Out(b3, " %11.5f", " %11.5f\n")
Out(a4, " %11.5f", " %11.5f")
Out(b4, " %11.5f", " %11.5f\n")
Out(a5, " %11.5f", " %11.5f")
Out(b5, " %11.5f", " %11.5f\n")
Out(a6, " %11.5f", " %11.5f")
Out(b6, " %11.5f", " %11.5f\n")
Out(a7, " %11.5f", " %11.5f")
Out(b7, " %11.5f", " %11.5f\n")
Out(a8, " %11.5f", " %11.5f")
Out(b8, " %11.5f", " %11.5f\n")
Out(a9, " %11.5f", " %11.5f")
Out(b9, " %11.5f", " %11.5f\n")
Out(a10, " %11.5f", " %11.5f")
Out(b10, " %11.5f", " %11.5f\n")
Out(a11, " %11.5f", " %11.5f")
Out(b11, " %11.5f", " %11.5f\n")

xdd_out ##filename##.xy load out_record out_fmt out_eqn
   {
        " %11.5f  " = X;
        " %11.5f  " = Ycalc;
        " %11.5f\n  " = Yobs;
   }
#endif
'}}}
```

Fig. S2. Input files in jEdit 4.3.1 text editor to extract the peak positions and FWHM of the peaks within the (a) cubic/pseudo-cubic or (b) tetragonal patterns, with errors and r_wp, as well as outputting the fits and raw patterns as xy files.

Both the Scherrer and Williamson-Hall methods for deriving the crystallite size from the PXRD patterns were utilised and later compared within the OSC prediction models. The Scherrer equation is:

$$D = \frac{k\lambda}{\beta_{hkl}}$$

Where the crystallite size (D) is calculated from the Shape factor *(k)* , generally taken as 0.9 [4], the wavelength *(λ)* , and the peak broadening β_{hkl}, calculated as the FWHM of the peak.

The Williamson-Hall method assumes separate size and strain contributions to the peak broadening (β_{hkl}) in the PXRD as:

$$\beta_{hkl} = \frac{k\lambda}{D\cos\theta} + 4\varepsilon\tan\theta$$

Where the $\frac{k\lambda}{D\cos\theta}$ term covers the contribution to the peak broadening from the size, and the $4\varepsilon\tan\theta$ term covers the contribution from the strain[4, 5]. This equation can be rearranged to:

$$\beta_{hkl}\cos\theta = \frac{k\lambda}{D} + 4\varepsilon\sin\theta$$

Which allows a plot of $\beta_{hkl}\cos\theta$ (y axis) against $4\sin\theta$ (x axis), giving a linear best fit with the gradient corresponding to the lattice strain ε, and the Williamson-Hall crystallite size (D) can be calculated from the intercept $\frac{k\lambda}{D}$ [6].

A.3.2. Raman Analysis

The automation of the Raman analysis was carried out using python scripts for plotting spectra for quick phase identification (Figure S3) as well as removing a linear background and extracting peak positions and intensities. The intensity ratio of the peaks at around 600 cm^{-1} and 460 cm^{-1} were also extracted (I_D/I_{F2g}, given in Figure S4).

```
import glob
import matplotlib.pyplot as plt

filenames = (glob.glob('*.txt')) #List of files.

Spectra_per_plot = int(input("Enter desired number of spectra per plot:"))
#Defines number of samples per plot.

a = 0
b = Spectra_per_plot
c = 0 #For offset.
d = 0 #For stopping loop.

while True:
    for file in filenames[a:b]: #Plot the raman spectra for each file and plot
in multiples of that specified above.
        if b > (len(filenames)+1): #Checks if there are still enough samples to
plot as defined above, if not, the residual samples are plot and the loop is
broken.
            a = a-Spectra_per_plot+(b%len(filenames))
            b = b-Spectra_per_plot+(b%len(filenames))
            d = 1 #Tells script the last set of samples have been selected.
        x = []
        y = []
        for line in open(file):
            values = [float(s) for s in line.split()]
            if values != []:
                x.append(values[0]) #Grabs Raman shifts for sample.
                y.append(values[1]+c) #Grabs Intesities for sample.
        plt.plot(x, y, label = file) #Adds each sample to the plot
        plt.legend()
        c += 0.5 #Adds an offset.
    a += Spectra_per_plot #Moves to select the next set of samples to plot.
    b += Spectra_per_plot #Moves to select the next set of samples to plot.
    plt.xlim(100, 800)
    plt.show()
    if d==1: #If all samples have been plot.
        break

print('Done') #To know when all samples have been plot.
```

Fig. S3. Python script for plotting the Raman spectra with an offset for a given number of spectra per plot, defined by user input.

```
import glob
import matplotlib.pyplot as plt

filenames = sorted(glob.glob('*.txt'))

###### Make Dictionaries

Raman_raw_x = {}
Raman_raw_y = {}

Raman_x_100to800 = {}
Raman_y_100to800 = {}

Raman_lower_bg_point_x = {}
Raman_lower_bg_point_y = {}
Raman_upper_bg_point_x = {}
Raman_upper_bg_point_y = {}

for f in filenames: #xy files empty dictionary.
    Raman_raw_x["{0}".format(f)] = []
    Raman_raw_y["{0}".format(f)] = []
    Raman_x_100to800["{0}".format(f)] = []
    Raman_y_100to800["{0}".format(f)] = []
    Raman_lower_bg_point_x["{0}".format(f)] = []
    Raman_lower_bg_point_y["{0}".format(f)] = []
    Raman_upper_bg_point_x["{0}".format(f)] = []
    Raman_upper_bg_point_y["{0}".format(f)] = []

###### Make x,y Dictionaries


for f in filenames:
    for line in open(f):
        values = [float(s) for s in line.split()]
        Raman_raw_x[f].append(values[0])      #x values in file.
        Raman_raw_y[f].append(values[1])   #y values in file.

for f in filenames: # Getting x 100 to 800 values in dictionaries.
    m=0
    for x in range(1,534):
        Raman_x_100to800[f].append(Raman_raw_x[f][2369+m])
        Raman_y_100to800[f].append(Raman_raw_y[f][2369+m])
        m=m+1

for f in filenames: #Getting points for BG gradient calc (2 points on raman
spectra for linear BG fit and removal) subraction.
    Raman_lower_bg_point_x[f].append(Raman_x_100to800[f][493])
    Raman_lower_bg_point_y[f].append(Raman_y_100to800[f][493])
    Raman_upper_bg_point_x[f].append(Raman_x_100to800[f][2])
    Raman_upper_bg_point_y[f].append(Raman_y_100to800[f][2])

###### Background Linear Gradients

dist_up = []
dist_across = []
gradient = {}
gradient_temp = []

###### Calclating Background Gradient

for f in filenames:
    gradient["{0}".format(f)] = []
    dist_up.append(Raman_lower_bg_point_y[f][0]-Raman_upper_bg_point_y[f][0])

dist_across.append(Raman_lower_bg_point_x[f][0]-Raman_upper_bg_point_x[f][0])
    gradient_temp.append(dist_up[0]/dist_across[0])
    gradient[f].append(gradient_temp[0])
    dist_up = []
    dist_across = []

###### Background Calculation

background_x = Raman_x_100to800[filenames[0]]
background_y = {}

for f in filenames:
    background_y["{0}".format(f)] = []
    z = 0
    for x in range(0,533):

background_y[f].append((Raman_x_100to800[f][z]*gradient[f][0])+((gradient[f][0]*
-Raman_lower_bg_point_x[f][0])+(Raman_lower_bg_point_y[f][0])))
        z = z+1
```

```
###### Subtracting Background

Subtracted_Raman_y_100to800 = {}
m = 0

for f in filenames:
    Subtracted_Raman_y_100to800["{0}".format(f)] = []
    m = 0
    for x in range(0,533):

Subtracted_Raman_y_100to800[f].append(Raman_y_100to800[f][m]-background_y[f][m])
        m=m+1
###### Extracting Intensities

Raman_x_400to550 = {}
Raman_y_400to550 = {}
Raman_x_580to650 = {}
Raman_y_580to650 = {}

If2g = {}
If2g_pos = {}
Id = {}
Id_pos = {}
Id_If2g_ratio = {}

for f in filenames:
    Raman_x_400to550["{0}".format(f)] = []
    Raman_y_400to550["{0}".format(f)] = []
    Raman_x_580to650["{0}".format(f)] = []
    Raman_y_580to650["{0}".format(f)] = []
    If2g["{0}".format(f)] = []
    If2g_pos["{0}".format(f)] = []
    Id["{0}".format(f)] = []
    Id_pos["{0}".format(f)] = []
    Id_If2g_ratio["{0}".format(f)] = []

for f in filenames: # Getting x 400 to 550 values in dictionaries.
    m=0
    for x in range(1,117): # 533 points in group.
        Raman_x_400to550[f].append(Raman_x_100to800[f][202+m])
        Raman_y_400to550[f].append(Subtracted_Raman_y_100to800[f][202+m])
        m=m+1

for f in filenames: # Getting x 580 to 650 values in dictionaries.
    m=0
    for x in range(1,55): # 533 points in group.
        Raman_x_580to650[f].append(Raman_x_100to800[f][123+m])
        Raman_y_580to650[f].append(Subtracted_Raman_y_100to800[f][123+m])
        m=m+1

for f in filenames:
    If2g[f].append(max(Raman_y_400to550[f]))
    Id[f].append(max(Raman_y_580to650[f]))
    Id_If2g_ratio[f].append(max(Raman_y_580to650[f])/max(Raman_y_400to550[f]))

If2g_pos[f].append(Raman_x_400to550[f][(Raman_y_400to550[f].index(max(Raman_y_40
0to550[f])))])

Id_pos[f].append(Raman_x_580to650[f][(Raman_y_580to650[f].index(max(Raman_y_580t
o650[f])))])
    print(If2g_pos[f][0])
    with open("Raman_Ratios.txt", "a") as file_object:
            file_object.write(f)
            file_object.write(","+str(If2g_pos[f][0]))
            file_object.write(","+str(If2g[f][0]))
            file_object.write(","+str(Id_pos[f][0]))
            file_object.write(","+str(Id[f][0]))
            file_object.write(","+str(Id_If2g_ratio[f][0]))
            file_object.write("\n")

###### Plotting

for f in filenames: #Plot raw and background.
    plt.plot(Raman_x_100to800[f], background_y[f], label = f )
    plt.plot(Raman_x_100to800[f], Raman_y_100to800[f], label = f )
plt.legend (loc=1)
plt.show()

for f in filenames: #Plot background subtracted spectra.
    plt.plot(Raman_x_100to800[f], Subtracted_Raman_y_100to800[f], label = f )

plt.legend (loc=1)
plt.show()
```

Fig. S4. Python script to remove a linear background from the Raman spectra, in the 100-800 cm^{-1} region, before extracting positions of the peaks at around 460 cm^{-1} and 600 cm^{-1}, and a ratio of peak intensities between the peaks at around 600 cm^{-1} and 460 cm^{-1}.

A.3.3. TGA Analysis

The analysis of TGA reduction measurements was automated by utilising macros that ran through each sample file, one after another in a sequentially automated way, within TA Instruments Universal Analysis 2000 software (v4.5A), outputting to an excel document. The macro used in this work is given in the Figure S5, obtaining the reduction peak temperature (calculated as the temperature of the peak in the derivative TG curve of weight with temperature) and the mass lost during reduction (calculated as the mass lost between the onset and offset temperature of the peak in the differential TG curve), exporting the profiles as text files. The output was then appended to a single text file via a python script (v3.7.4), given in Figure S6.

```
Program: Universal Analysis V4.5A
Macro: TGA
Steps: 29
Created: 15-Nov-2022 13:46:34
Revised: 15-Nov-2022 14:13:35

1: Function Open Data File
2: Button Signals... Data File Information
3: Button Default Signal Selection
4: Select Item 4 Y2 Signal  Weight (%)
5: Select Item 6 Y2 Type  Derivative (temp)
6: Button OK Signal Selection
7: Button OK Data File Information
8: Function Signal Change
9: Cursor Point 1 (400.0, 95.8866)
10: Cursor Point 2 (665.0, 94.3497)
11: Function Accept Cursor Limits
12: Function Export Plot Signals Only
13: Button Options ASCII data file
14: Check No parameter block
15: Check No data breaks or flags
16: Check No invalid data points
17: Button Finish >> Export Data File
18: Function Results Spreadsheet
19: Function Open Data File
20: Button Signals... Data File Information
21: Button Default Signal Selection
22: Select Item 6 Y1 Type  Derivative (temp)
23: Button OK Signal Selection
24: Button OK Data File Information
25: Function Peak Maximum
26: Cursor Point 1 (400.0, .00182709)
27: Cursor Point 2 (665.0, .00272479)
28: Function Accept Cursor Limits
29: Function Results Spreadsheet
```

Fig. S5. Within TA Instruments Universal Analysis 2000 software, the macro used to analyse each TGA profile for reduction peak temperature and mass loss during reduction, as well as exporting the plots to an excel spreadsheet document.

```
from openpyxl import load_workbook
import glob

filenames = sorted(glob.glob('*.xlsx'))
m=0
for f in filenames:
    print(str(m)+": "+f)
    m=m+1

data_file = filenames[int(input("Type number of file for analysis:"))]
print("Selected "+data_file+" for analysis")
wb = load_workbook(data_file, data_only=True)
number_of_samples = int((len(wb.sheetnames)/2))
TGA_ML = {}
TGA_RP = {}
a = -1

### Mass Loss Extraction
for sheet in list(reversed(wb.sheetnames)):
    a += 1
    if float(sheet[5:])%2 != 0: #If it is odd (e.g. Sheet1, Sheet3, ...)
        TGA_ML[a] = wb[sheet].cell(4,3).value
    else:
        a -= 1
        continue

### Redution Peak Temperature Extraction
a = -1
for sheet in list(reversed(wb.sheetnames)):
    a += 1
    if float(sheet[5:])%2 == 0: #If it is even (e.g. Sheet2, Sheet4, ...)
        TGA_RP[a] = wb[sheet].cell(4,3).value
    else:
        a -= 1
        continue

### Output

with open("TGA_Output.txt", "a") as file_object:
    file_object.write("Sample,Reduction Mass Loss (Wt.%),Max Reduction Peak 
Temperature (oC)")
    file_object.write("\n")

for i in range(0, number_of_samples):
    with open("TGA_Output.txt", "a") as file_object:
        file_object.write(str(int(i+1))+","+str(TGA_ML[i])+","+str(TGA_RP[i]))
        file_object.write("\n")
```

Fig. S6. Python script for extracting the reduction peak temperature and mass loss during reduction values output into an excel spreadsheet from the macro in Figure S5 into a single text file.

A.4. Prediction Modelling

A.4.1. Stepwise Model Development

OSC prediction models were developed using the model screening functionality within JMP Pro 16 software.

Several multiple linear regression models were trialled through a stepwise approach, considering both forward selection and backward elimination methods, starting from either a null or full model, subsequently adding or removing variables, respectively. The stepwise approach is such that after a variable has been entered or removed (forward selection or backwards elimination methods, respectively), all already entered or remaining variables are tested to see if any should be added or removed according to the removal criteria[7]. The best models that were selected shown a minimisation of the Akaike information criterion and the Bayesian information criterion (AICc and BIC), as well as a minimisation of the root mean square error (RMSE), which all select the best model out of those tested[8]. It was also important for the final model to demonstrate a high level of significance for each parameter, with low p-values (<0.10), significant absolute t-values (an absolute value greater than 2), indicating significance in non-zero parameter estimates[9], and a high F-ratio (larger is better when comparing models); which indicates that the full model gives a better fit than a simple mean[9].

A.4.2. Statistical Output

After constructing a multiple linear regression model in JMP through the stepwise approach described in the previous section, tables are output containing statistics pertaining to the quality of the model produced. The summary of fit, analysis of variance, and parameter estimates tables are output for models in this study.

The summary of fit table contains several statistics relating to the overall model. The R^2 and R^2 adjusted for the number of parameters is given, indicating how much of the variance around the mean is accounted for by the model[10]. The root mean square error (RMSE) is also provided, which is the standard deviation of the residual errors in model predictions[10], so minimising this is optimal. The mean of response shows the average experimental OSC value, and the observations are the number of samples making up the training set of the model.

The analysis of variance table provides calculations comparing the fitted model to a model where all predicted values equal the mean of the response (experimental OSC), provided in the summary of fit table. The sum of squared residuals for the full fitted model is given (as the Error Sum of Squares in the analysis of variance table), along with the sum of squared residuals when only fitting the mean of the response (given as C. Total Sum of Squares in the analysis of variance table)[10]. The difference between these values is the Model sum of squares which is a measure for how much of the variation is accounted for by the variables in the model (given as Model Sum of Squares in the analysis of variance table). The mean squares are the sum of squares values divided by the degrees of freedom, given as “DF” in the table[10]. The F ratio is then calculated as the Model mean square divided by the Error mean square, where a large value, confirmed by a small p-value (Given under “Prob > F” in table, ideally less than 0.05), indicates that the fitted model is better than a simple mean fit[10]:

$$F\ ratio = \frac{Mean\ Square\ of\ the\ Model}{Mean\ Square\ of\ the\ Error}$$

The parameter estimates table gives the estimates of the model coefficients relating to each variable in the model and the respective standard error, along with a calculated t ratio and p-value against a hypothesis that the true value of each parameter is zero. The t ratio is calculated as[10]:

$$t\,ratio = \frac{estimate - hypothesised\ value}{standard\ error\ of\ the\ estimate}$$

For the hypothesis that the estimates of zero, this leaves the t ratio as the value of the estimate divided by the standard error of the estimate, with both values provided in the parameter estimates table for every variable within the model. Generally a t ratio greater than an absolute value of 2 is desired to show significance at the 0.05 level[10]. The p-value provided in the parameter estimates table is the probability that the true parameter value is zero.

B. OSC Measurements of Undoped Robotic Samples Compared to References

The materials produced by the robotic synthesis route described in section A were compared to reference materials provided by Johnson Matthey PLC.

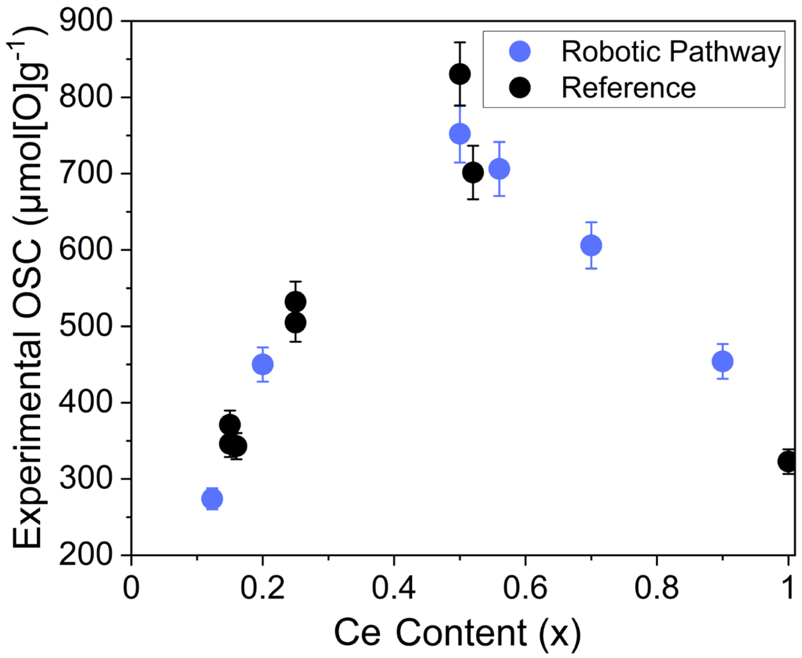

Fig. S7. Experimental OSC of $Ce_xZr_{1-x}O_{2-\delta}$ materials synthesised via the robotic-based co-precipitation pathway (blue) and reference materials (black), across $0 < x \leq 1$.

Before screening libraries of materials and building OSC prediction models, confidence that the OSC of the materials produced from the robotic-based pathway match industrial references, in terms of the oxygen storage capacity, is vital. This was investigated by measuring some undoped materials ($Ce_xZr_{1-x}O_{2-\delta}$) and Figure S7 shows the experimentally measured oxygen storage capacity of the reference materials with those produced by the robotic-based pathway. Crucially, the materials produced in this study gave OSC values comparable to the reference materials, giving the same parabolic trend with Ce content. This solidifies confidence in the robotic-based synthesis route for producing materials competent for the development of a useful OSC prediction model.

C. Materials Screening

C.1. Compositions Screened

C.1.1. Varying Ce Content with Constant Dopant Content

Below is a list of the compositions screened with the general formula $Ce_xZr_{1-x-y}Ln_yO_{2-\delta}$, where the stated lanthanide dopant content (y) is fixed and the Ce content (x) is varied, for undoped, single dopant (La, Y, Pr and Nd), and mixed dopant (La-Y and La-Nd) systems.

Table S1. The compositions screened with the general formula $Ce_xZr_{1-x-y}Ln_yO_{2-\delta}$, where the stated lanthanide dopant content (y) is fixed and the Ce content (x) is varied, for undoped, single dopant (La, Y, Pr and Nd), and mixed dopant (La-Y and La-Nd) systems. The general formulas of each dopant system are tabulated, along with the overall Ce content range screened and the Ce content range where thermally stable materials are observed, retaining a single phase after ageing via both PXRD and Raman measurements (full PXRD patterns and Raman spectra are provided in supplementary sections C2.1 and C3.1).

Composition	Ce Content Range	Thermally Stable Ce Content Range
$Ce_xZr_{1-x}O_2$	$0.08 \leq x \leq 0.60$	$0.08 \leq x \leq 0.22$
$Ce_xZr_{0.91-x}La_{0.09}O_{2-\delta}$	$0.06 \leq x \leq 0.50$	$0.36 \leq x \leq 0.50$
$Ce_xZr_{0.89-x}La_{0.11}O_{2-\delta}$	$0.07 \leq x \leq 0.54$	$0.36 \leq x \leq 0.53$
$Ce_xZr_{0.88-x}Y_{0.12}O_{2-\delta}$	$0.07 \leq x \leq 0.53$	$0.07 \leq x \leq 0.53$
$Ce_xZr_{0.85-x}Y_{0.15}O_{2-\delta}$	$0.07 \leq x \leq 0.51$	$0.07 \leq x \leq 0.51$
$Ce_xZr_{0.89-x}Nd_{0.11}O_{2-\delta}$	$0.07 \leq x \leq 0.53$	$0.07 \leq x \leq 0.50$
$Ce_xZr_{0.89-x}Pr_{0.11}O_{2-\delta}$,	$0.07 \leq x \leq 0.53$	$0.29 \leq x \leq 0.53$
$Ce_xZr_{0.9-x}La_{0.05}Y_{0.05}O_{2-\delta}$	$0.07 \leq x \leq 0.54$	$0.07 \leq x \leq 0.54$
$Ce_xZr_{0.9-x}La_{0.05}Nd_{0.05}O_{2-\delta}$	$0.07 \leq x \leq 0.54$	$0.29 \leq x \leq 0.54$

C.1.2. Increasing Dopant Content with a Constant Ce/Zr Ratio of 1

Below is a list of the compositions screened with the general formula $Ce_{(1-y)/2}Zr_{(1-y)/2}Ln_yO_{2-\delta}$, keeping the Ce/Zr ratio constant at 1 while increasing the stated lanthanide (y), for single dopant (La, Y, Pr and Nd) and mixed dopant (La-Y and La-Nd) systems.

Table S2. The compositions screened with the general formula $Ce_{(1-y)/2}Zr_{(1-y)/2}Ln_yO_{2-\delta}$, keeping the Ce/Zr ratio constant at 1 while increasing the stated lanthanide dopant content (y), for single dopant (La, Y, Pr and Nd) and mixed dopant (La-Y and La-Nd) systems. The general formulas of each dopant system are tabulated, along with the overall dopant content range screened and the dopant content range where thermally stable materials are observed, retaining a single phase after ageing via both PXRD and Raman measurements (full PXRD patterns and Raman spectra are provided in supplementary sections C2.2 and C3.2).

Composition	Dopant Content Range	Thermally Stable Dopant Content Range
$Ce_{(1-y)/2}Zr_{(1-y)/2}La_yO_{2-\delta}$	$0 \leq y \leq 0.21$	$0.11 \leq y \leq 0.21$
$Ce_{(1-y)/2}Zr_{(1-y)/2}Y_yO_{2-\delta}$	$0 \leq y \leq 0.25$	$0.09 \leq y \leq 0.25$
$Ce_{(1-y)/2}Zr_{(1-y)/2}Nd_yO_{2-\delta}$	$0 \leq y \leq 0.27$	$0.10 \leq y \leq 0.27$
$Ce_{(1-y)/2}Zr_{(1-y)/2}Pr_yO_{2-\delta}$	$0 \leq y \leq 0.26$	$0.08 \leq y \leq 0.26$
$Ce_{(0.95-y)/2}Zr_{(0.95-y)/2}La_{0.05}Y_yO_{2-\delta}$	$0 \leq y \leq 0.12$	$0.08 \leq y \leq 0.12$
$Ce_{(0.95-y)/2}Zr_{(0.95-y)/2}La_{0.05}Nd_yO_{2-\delta}$	$0 \leq y \leq 0.13$	$0.04 \leq y \leq 0.13$
$Ce_{(0.95-y)/2}Zr_{(0.95-y)/2}La_yNd_{0.05}O_{2-\delta}$	$0 \leq y \leq 0.16$	$0.05 \leq y \leq 0.16$

C.2. PXRD Patterns

C.2.1. Varying Ce/Zr Ratio with Constant Dopant Content

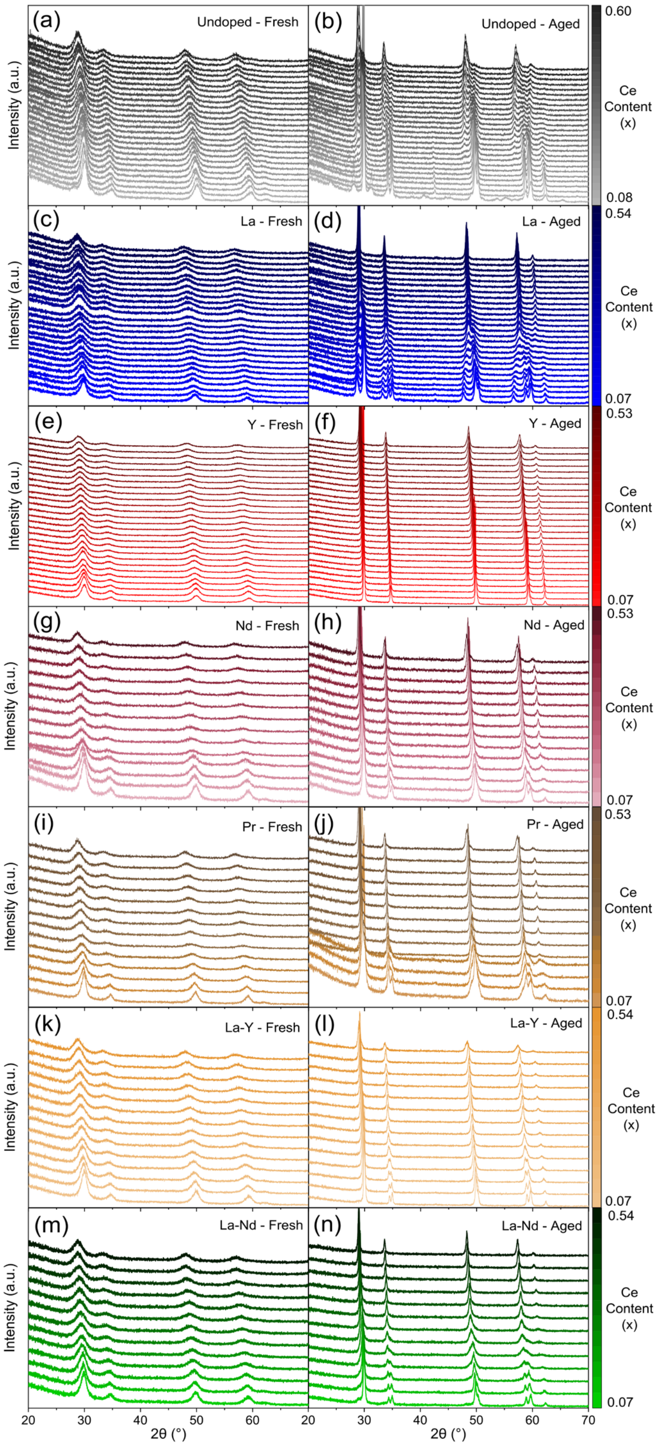

Fig. S8. PXRD patterns for the (left column) fresh and (right column) thermally aged materials with the general formula $Ce_xZr_{1-x-y}Ln_yO_{2-\delta}$, keeping the stated lanthanide dopant content (y) constant while varying Ce content (x), for (a, b) Undoped (y = 0, 0.08 ≤ x ≤ 0.60), (c, d) 11% La doped (y = 0.11, 0.07 ≤ x ≤ 0.54), (e, f) 12% Y doped (y = 0.12, 0.07 ≤ x ≤ 0.53), (g, h) 11% Nd doped (y = 0.11, 0.07 ≤ x ≤ 0.53), (i, j) 11% Pr doped (y = 0.11, 0.07 ≤ x ≤ 0.53), (k, l) 5% La 5% Y doped (y = 0.10, where $Ln_y = La_{0.05}Y_{0.05}$ as there are two dopants, 0.07 ≤ x ≤ 0.54) and (m, n) 5% La 5% Nd doped materials (y = 0.10, where $Ln_y = La_{0.05}Nd_{0.05}$ as there are two dopants, 0.07 ≤ x ≤ 0.54).

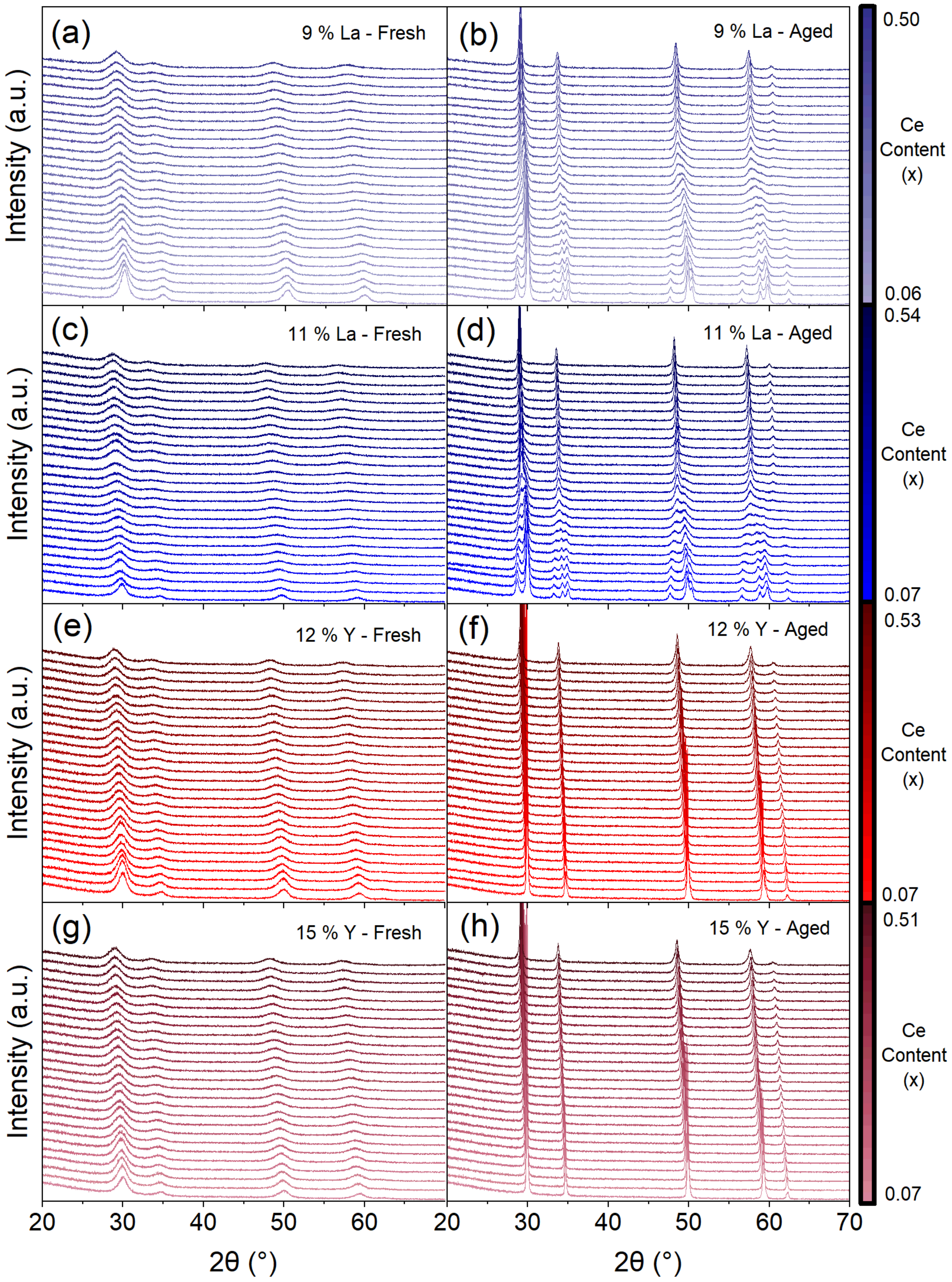

Fig. S9. PXRD patterns for the (left column) fresh and (right column) thermally aged materials with the general formula $Ce_xZr_{1-x-y}Ln_yO_{2-\delta}$, keeping the stated lanthanide dopant content (y) while varying Ce content (x), for (a, b) 9% La doped (y = 0.09, 0.06 ≤ x ≤ 0.50), (c, d) 11% La doped (y = 0.11, 0.07 ≤ x ≤ 0.54), (e, f) 12% Y doped (y = 0.12, 0.07 ≤ x ≤ 0.53) and (g, h) 15% Y doped materials (y = 0.15, 0.07 ≤ x ≤ 0.51).

C.2.2. Increasing Dopant Content with a Constant Ce/Zr Ratio of 1

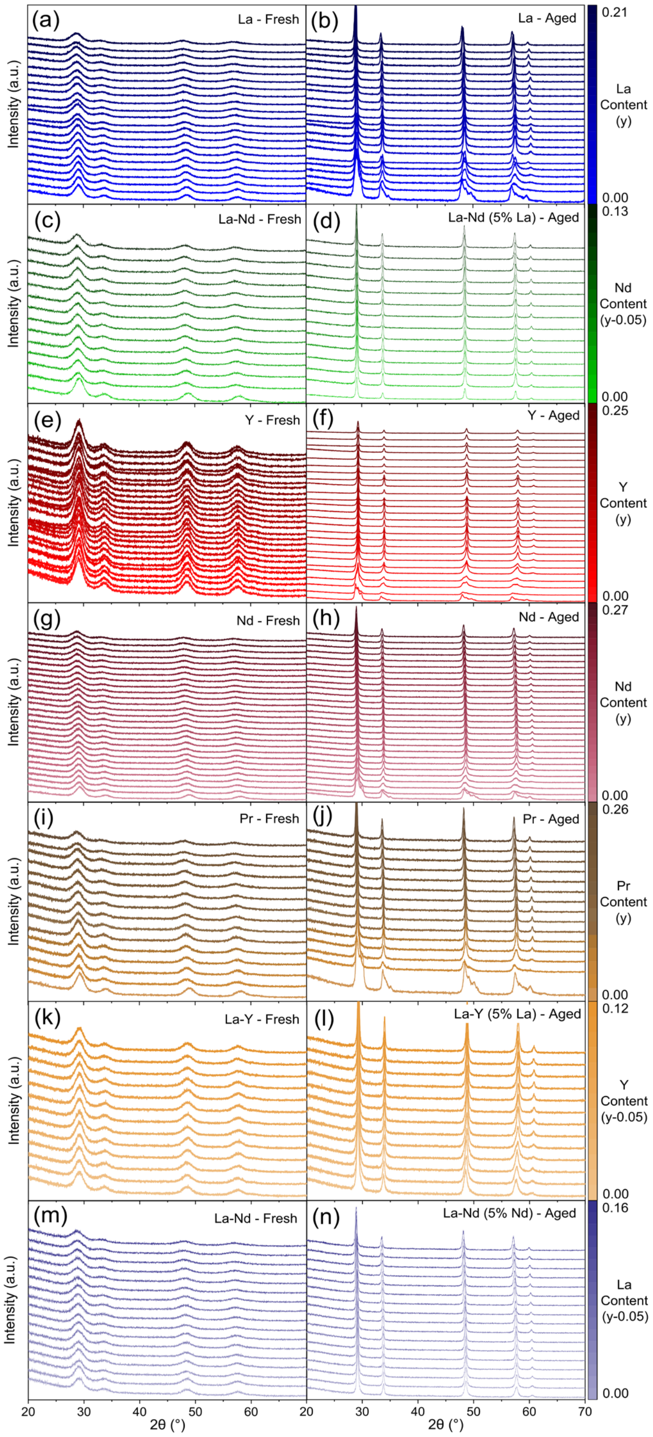

Fig. S10. PXRD patterns of doped ceria-zirconia materials for (left column) fresh and (right column) thermally aged materials with the general formula $Ce_{(1-y)/2}Zr_{(1-y)/2}Ln_yO_{2-\delta}$, keeping Ce/Zr constant at 1 while increasing the stated lanthanide dopant content (y). PXRD patterns are given for (a, b) La doped ($0.00 \leq y \leq 0.21$), (c, d) La-Nd doped with a constant 5% La (where $Ln_y = La_{0.05}Nd_n$ as there are two dopants, $0.00 \leq n \leq 0.13$, $y = 0.05 + n$), (e, f) Y doped ($0.00 \leq y \leq 0.25$), (g, h) Nd doped ($0.00 \leq y \leq 0.27$), (i, j) Pr doped ($0.00 \leq y \leq 0.26$), (k, l) La-Y doped with a constant 5% La (where $Ln_y = La_{0.05}Y_n$ as there are two dopants, $0.00 \leq n \leq 0.12$, $y = 0.05 + n$), and (m, n) La-Nd doped materials with a constant 5% Nd (where $Ln_y = La_nNd_{0.05}$ as there are two dopants, $0.00 \leq n \leq 0.16$, $y = 0.05 + n$).

C.3. Raman Spectra

C.3.1. Varying Ce/Zr Ratio with Constant Dopant Content

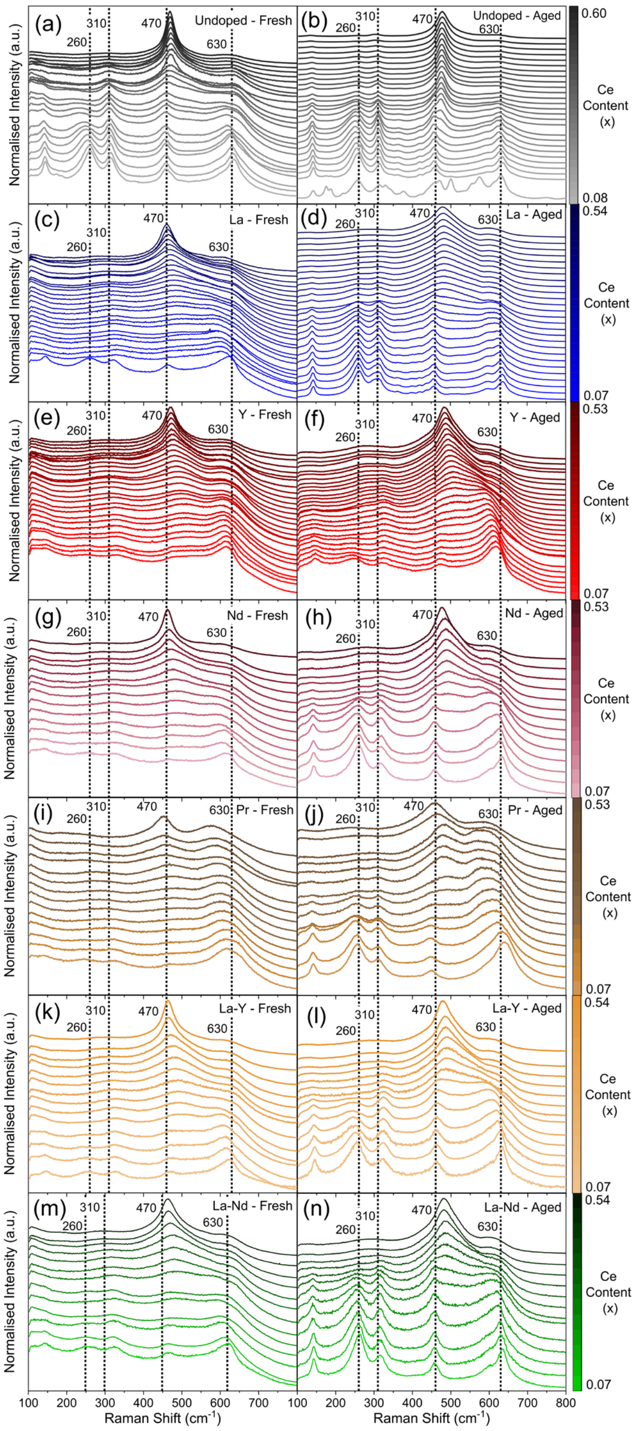

Fig. S11. Raman spectra for the (left column) fresh and (right column) thermally aged materials with the general formula $Ce_xZr_{1-x-y}Ln_yO_{2-\delta}$, keeping the stated lanthanide dopant content (y) constant while varying Ce content (x), for (a, b) Undoped (y = 0, 0.08 ≤ x ≤ 0.60), (c, d) 11% La doped (y = 0.11, 0.07 ≤ x ≤ 0.54), (e, f) 12% Y doped (y = 0.12, 0.07 ≤ x ≤ 0.53), (g, h) 11% Nd doped (y = 0.11, 0.07 ≤ x ≤ 0.53), (i, j) 11% Pr doped (y = 0.11, 0.07 ≤ x ≤ 0.53), (k, l) 5% La 5% Y doped (y = 0.10, where Ln_y = $La_{0.05}Y_{0.05}$ as there are two dopants, 0.07 ≤ x ≤ 0.54) and (m, n) 5% La 5% Nd doped materials (y = 0.10, where Ln_y = $La_{0.05}Nd_{0.05}$ as there are two dopants, 0.07 ≤ x ≤ 0.54).

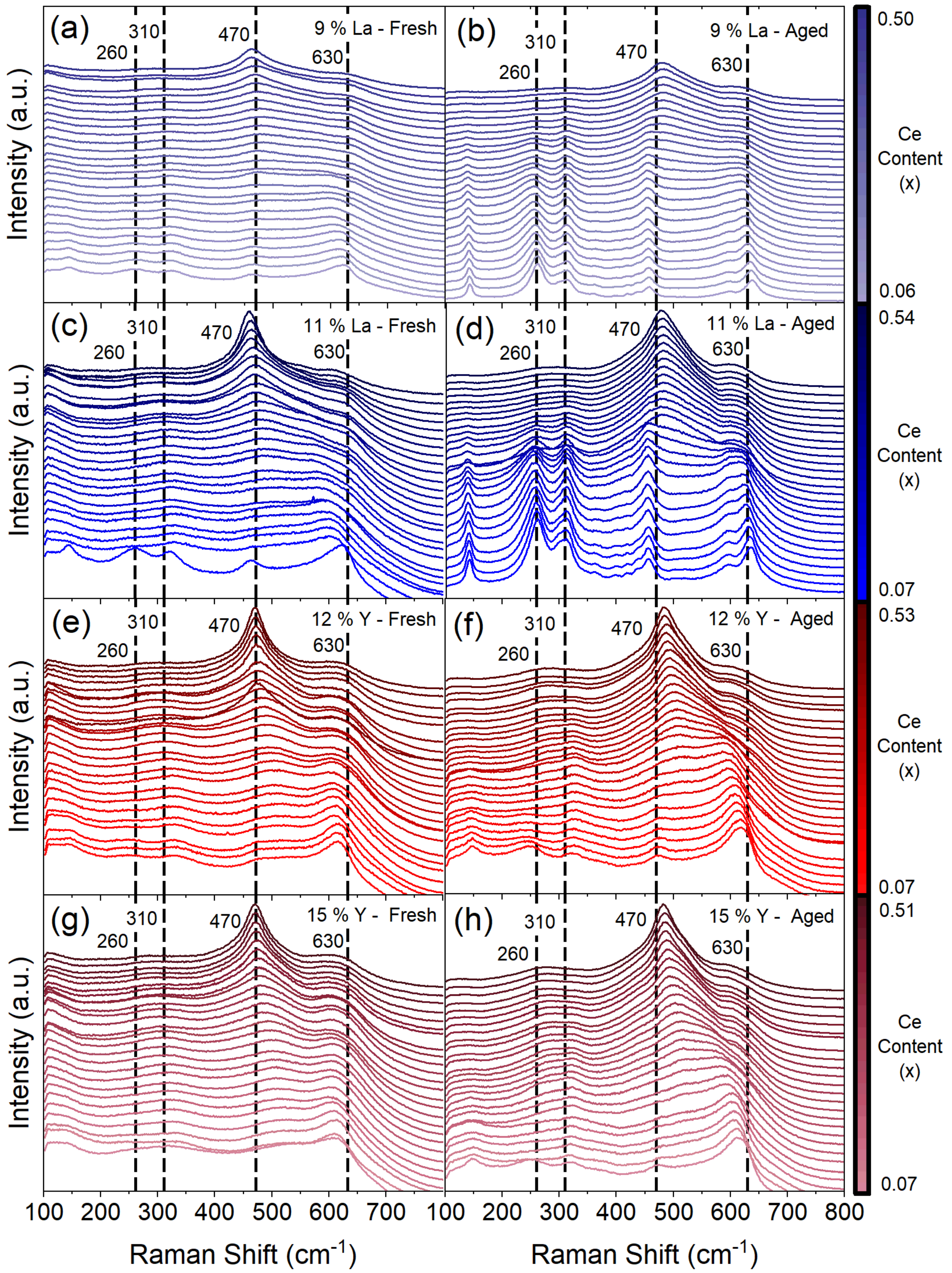

Fig. S12. Raman spectra for the (left column) fresh and (right column) thermally aged materials with the general formula $Ce_xZr_{1-x-y}Ln_yO_{2-\delta}$, keeping dopant content (y) constant while varying Ce content (x), for (a, b) 9% La doped (y = 0.09, 0.06 ≤ x ≤ 0.50), (c, d) 11% La doped (y = 0.11, 0.07 ≤ x ≤ 0.54), (e, f) 12% Y doped (y = 0.12, 0.07 ≤ x ≤ 0.53) and (g, h) 15% Y doped materials (y = 0.15, 0.07 ≤ x ≤ 0.51).

C.3.2. Increasing Dopant Content with a Constant Ce/Zr Ratio of 1

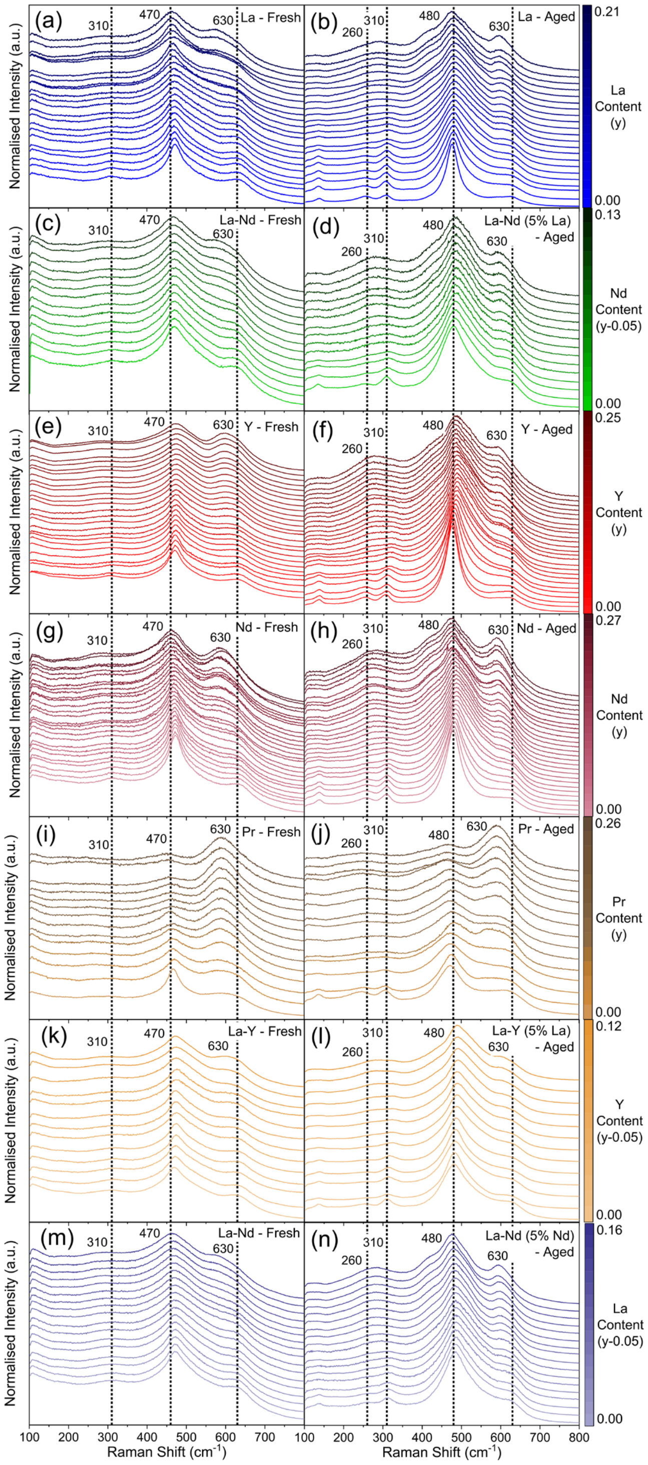

Fig. S13. Raman spectra of doped ceria-zirconia materials for (left column) fresh and (right column) thermally aged materials with the general formula $Ce_{(1-y)/2}Zr_{(1-y)/2}Ln_yO_{2-\delta}$, keeping Ce/Zr constant at 1 while increasing the stated lanthanide dopant content (y). Raman spectra are given for (a, b) La doped ($0.00 \leq y \leq 0.21$), (c, d) La-Nd doped with a constant 5% La (where $Ln_y = La_{0.05}Nd_n$ as there are two dopants, $0.00 \leq n \leq 0.13$, $y = 0.05 + n$), (e, f) Y doped ($0.00 \leq y \leq 0.25$), (g, h) Nd doped ($0.00 \leq y \leq 0.27$), (i, j) Pr doped ($0.00 \leq y \leq 0.26$), (k, l) La-Y doped with a constant 5% La (where $Ln_y = La_{0.05}Y_n$ as there are two dopants, $0.00 \leq n \leq 0.12$, $y = 0.05 + n$), and (m, n) La-Nd doped materials with a constant 5% Nd (where $Ln_y = La_nNd_{0.05}$ as there are two dopants, $0.00 \leq n \leq 0.16$, $y = 0.05 + n$).

C.4. Lattice Parameters

C.4.1. Varying Ce/Zr Ratio with Constant Dopant Content

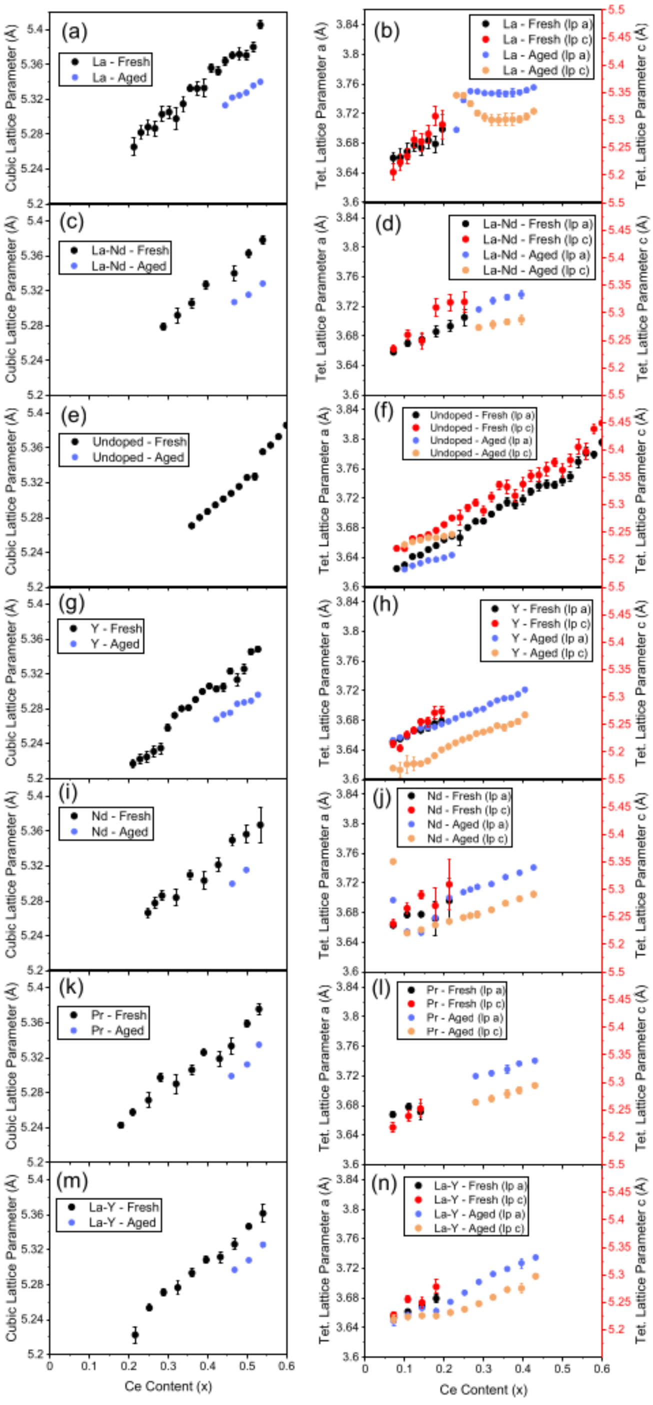

Fig. S14. (left column) pseudo-cubic and (right column) tetragonal lattice parameters for the (black/red) fresh and (blue/yellow) thermally aged materials with the general formula $Ce_xZr_{1-x-y}Ln_yO_{2-\delta}$, keeping the stated lanthanide dopant content (y) constant while varying Ce content (x). Given for (a, b) 11% La doped (y = 0.11, 0.07 ≤ x ≤ 0.54), (c, d) 5% La 5% Nd doped (y = 0.10, where Ln_y = $La_{0.05}Nd_{0.05}$ as there are two dopants, 0.07 ≤ x ≤ 0.54), (e, f) undoped (y = 0, 0.08 ≤ x ≤ 0.60), (g, h) 12% Y doped (y = 0.12, 0.07 ≤ x ≤ 0.53), (i, j) 11% Nd doped (y = 0.11, 0.07 ≤ x ≤ 0.53), (k, l) 11% Pr doped (y = 0.11, 0.07 ≤ x ≤ 0.53) and (m, n) 5% La 5% Y doped materials (y = 0.10, where Ln_y = $La_{0.05}Y_{0.05}$ as there are two dopants, 0.07 ≤ x ≤ 0.54). Lattice parameters are displayed for materials demonstrating a single phase in the PXRD patterns (Figure S8).

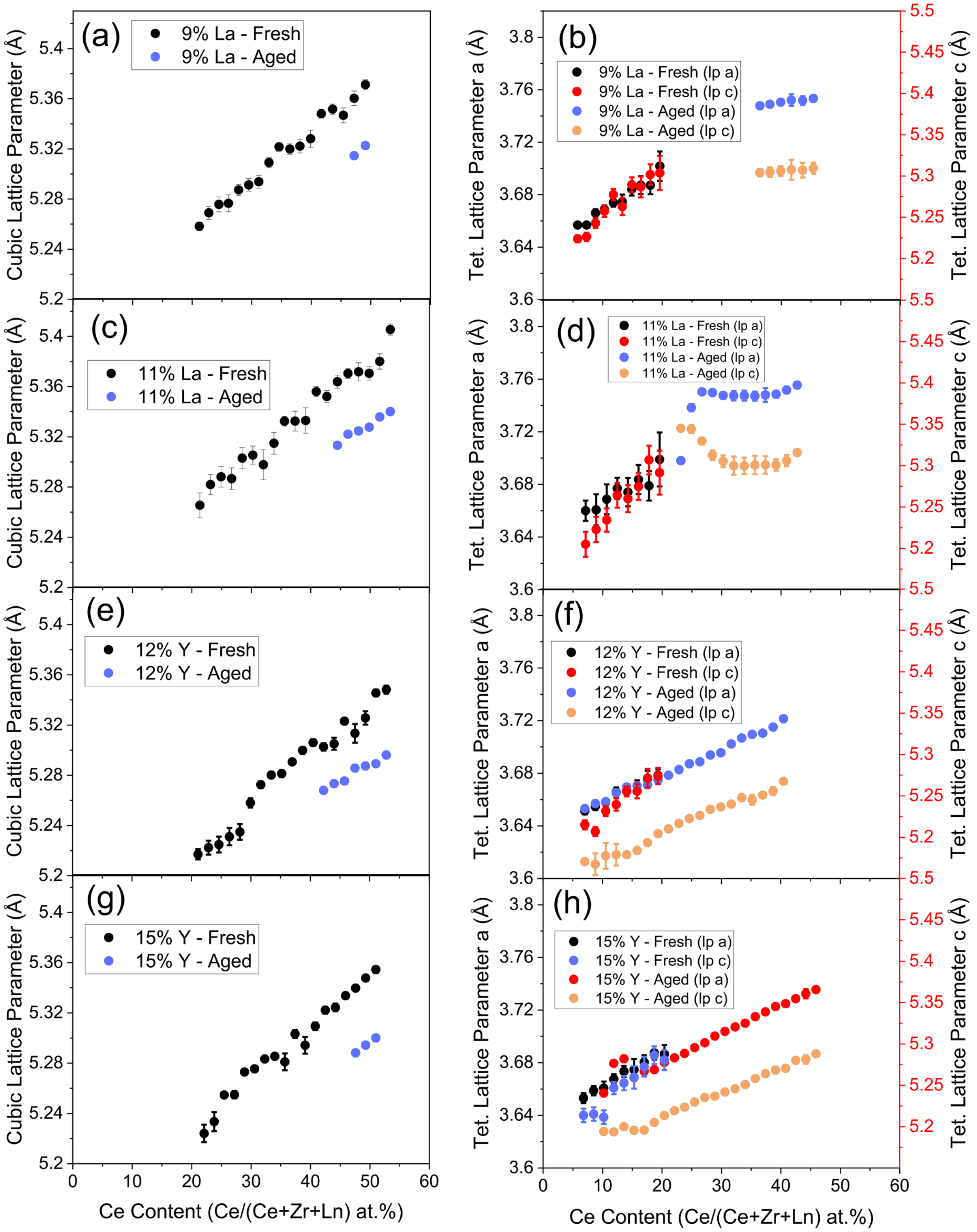

Fig. S15. (left column) pseudo-cubic and (right column) tetragonal lattice parameters for the (black/red) fresh and (blue/yellow) thermally aged materials with the general formula $Ce_xZr_{1-x-y}Ln_yO_{2-\delta}$, keeping the stated lanthanide dopant content (y) constant while varying Ce content (x), for (a, b) 9% La doped (y = 0.09, 0.06 ≤ x ≤ 0.50), (c, d) 11% La doped (y = 0.11, 0.07 ≤ x ≤ 0.54), (e, f) 12% Y doped (y = 0.12, 0.07 ≤ x ≤ 0.53) and (g, h) 15% Y doped materials (y = 0.15, 0.07 ≤ x ≤ 0.51). Lattice parameters are displayed for materials demonstrating a single phase in the PXRD patterns (Figure S9).

C.4.2. Increasing Dopant Content with a Constant Ce/Zr Ratio of 1

Lattice parameters were extracted for both fresh and thermally aged materials with the general formula $Ce_{(1-y)/2}Zr_{(1-y)/2}Ln_yO_{2-\delta}$, keeping the Ce/Zr ratio constant at 1 while increasing dopant content (y) for single dopant (La, Y, Pr and Nd) and mixed dopant (La-Y and La-Nd) systems. Here we observe that lattice parameters increase with total dopant content.

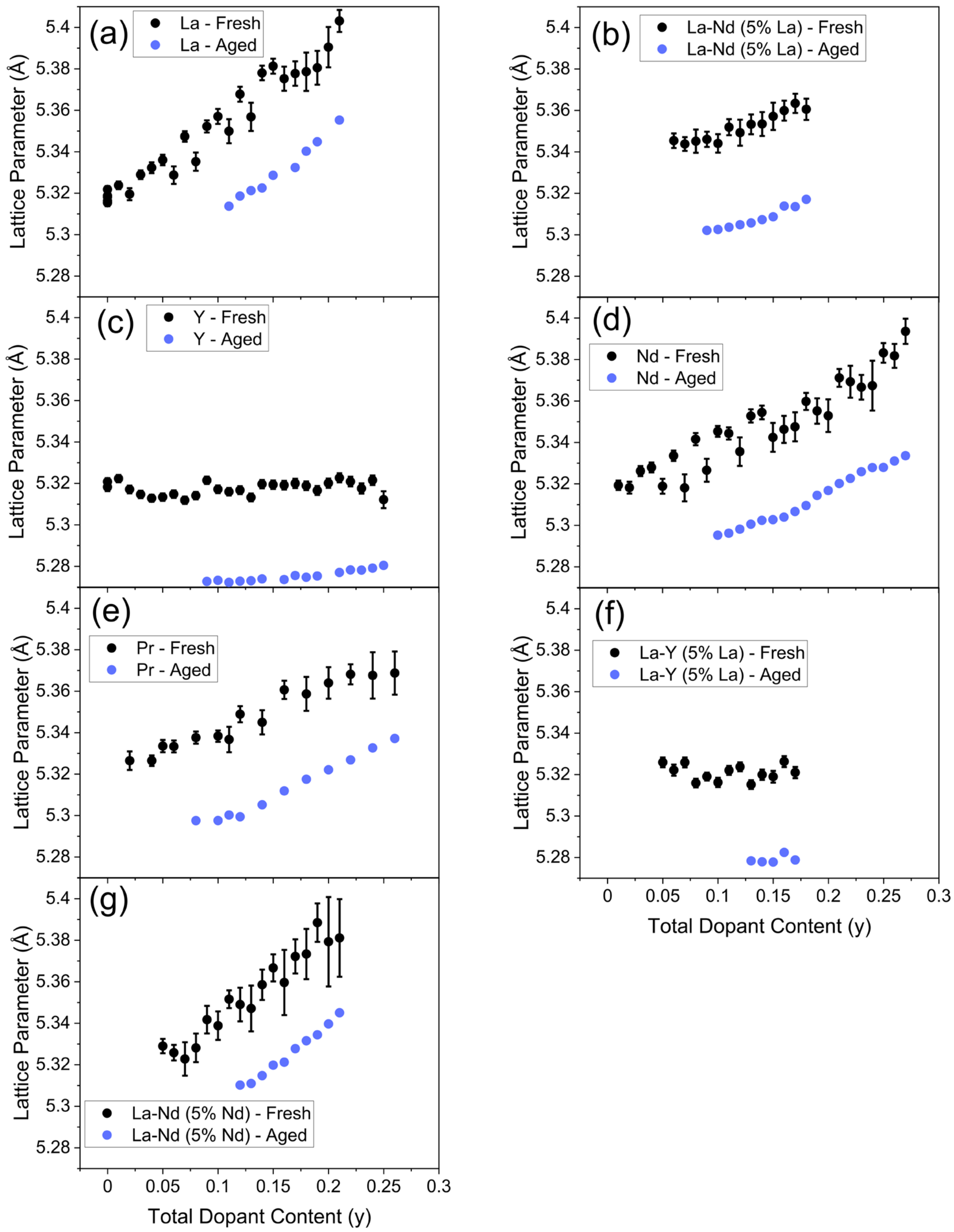

Fig. S16. Pseudo-cubic lattice parameters for (black) fresh and (blue) thermally aged materials with the general formula $Ce_{(1-y)/2}Zr_{(1-y)/2}Ln_yO_{2-\delta}$, keeping Ce/Zr constant at 1 while increasing the stated lanthanide dopant content (y). Lattice parameters are given for (a) La doped ($0.00 \leq y \leq 0.21$), (b) La-Nd doped with a constant 5% La (where $Ln_y = La_{0.05}Nd_n$ as there are two dopants, $0.00 \leq n \leq 0.13$, $y = 0.05 + n$), (c) Y doped ($0.00 \leq y \leq 0.25$), (d) Nd doped ($0.00 \leq y \leq 0.27$), (e) Pr doped ($0.00 \leq y \leq 0.26$), (f) La-Y doped with a constant 5% La (where $Ln_y = La_{0.05}Y_n$ as there are two dopants, $0.00 \leq n \leq 0.12$, $y = 0.05 + n$), and (g) La-Nd doped materials with a constant 5% Nd (where $Ln_y = La_nNd_{0.05}$ as there are two dopants, $0.00 \leq n \leq 0.16$, $y = 0.05 + n$).

C.5. ICP Analysis

Table S3. Ce and Zr elemental analysis obtained by ICP on the supernatant removed after centrifugation from a $Ce_{0.5}Zr_{0.5}O_{2-\delta}$ precipitate formed during the robotic synthesis route.

Element	Input Conc. (mg/L)	Conc. Removed after Centrifugation (mg/L)	Proportion of Overall Metal Content (%)
Ce	7735	0.008	1.03E-04
Zr	5036	0.000	0

ICP was taken on the supernatant from the precipitate after centrifugation for a $Ce_{0.5}Zr_{0.5}O_{2-\delta}$ material synthesised using the robotic coprecipitation pathway. From Table S3, the loss of the input Ce and Zr during the synthesis is negligible, supporting the conclusion that the input and output metal contents are close to identical.

C.6. SEM-EDS Analysis

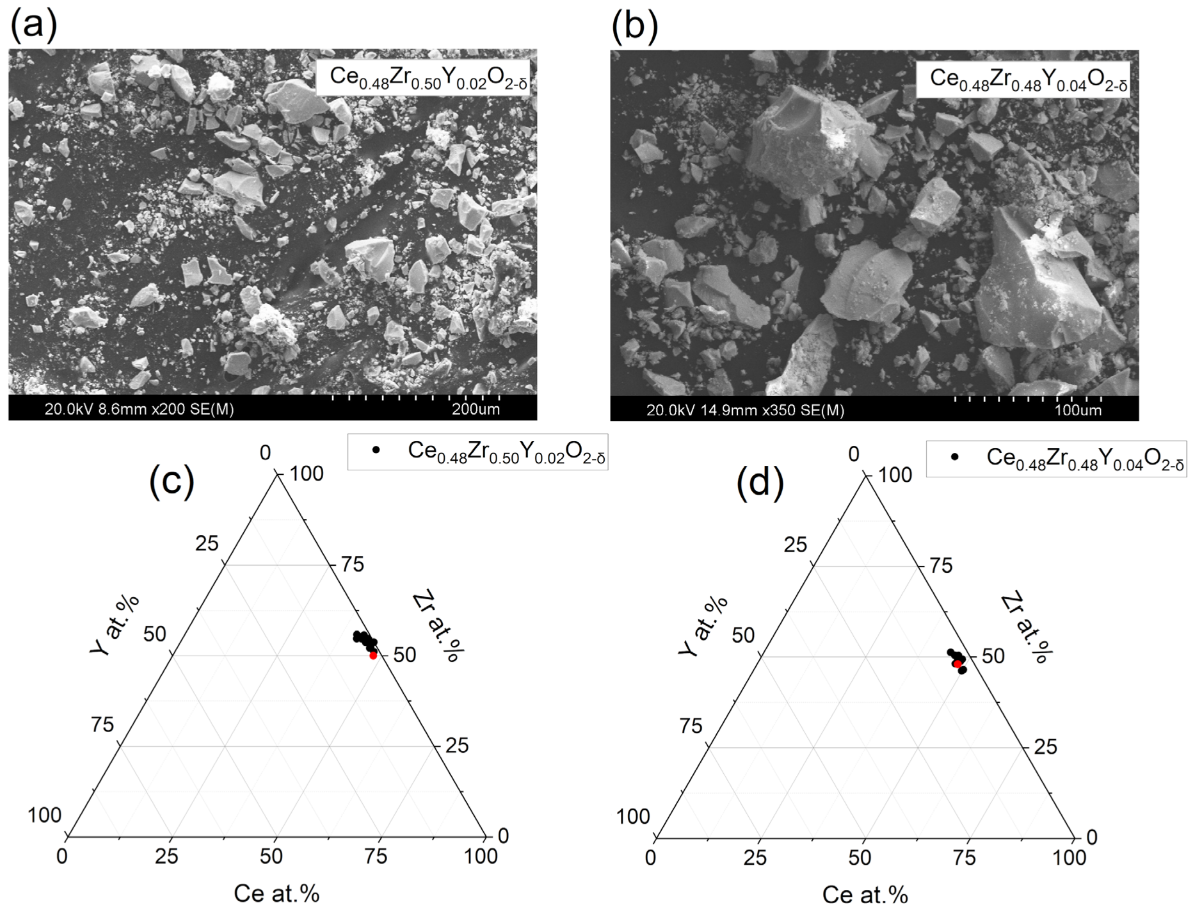

Fig. S17. SEM images and SEM-EDS measurements were taken on two Y-doped ceria-zirconia synthesised by the robotic pathway. SEM images were taken for a (a) $Ce_{0.48}Zr_{0.50}Y_{0.02}O_{2-\delta}$ material and a (b) $Ce_{0.48}Zr_{0.48}Y_{0.04}O_{2-\delta}$ material produced using the robotic method. SEM-EDS results are plot as a ternary diagram for the (c) $Ce_{0.48}Zr_{0.50}Y_{0.02}O_{2-\delta}$ material and the (d) $Ce_{0.48}Zr_{0.48}Y_{0.04}O_{2-\delta}$ material produced using the robotic method. The red points indicate the stoichiometric formula.

SEM images and SEM-EDS compositional information were gathered for two Y-doped materials produced using the robotic method. The particles in the images of both materials are homogenous

and the compositions of each sample as measured by SEM-EDS demonstrates homogeneity through a narrow compositional distribution about the expected composition.

C.7. Williamson-Hall Analyses

C.7.1. Varying Ce/Zr Ratio with Constant Dopant Content

Extracted Williamson-Hall crystallite sizes and strains from PXRD for fresh and thermally aged materials with the general formula $Ce_xZr_{1-x-y}Ln_yO_{2-\delta}$, where the stated lanthanide dopant content (y) is fixed and the Ce content (x) is varied, for undoped, single dopant (La, Y, Pr and Nd), and mixed dopant (La-Y and La-Nd) systems.

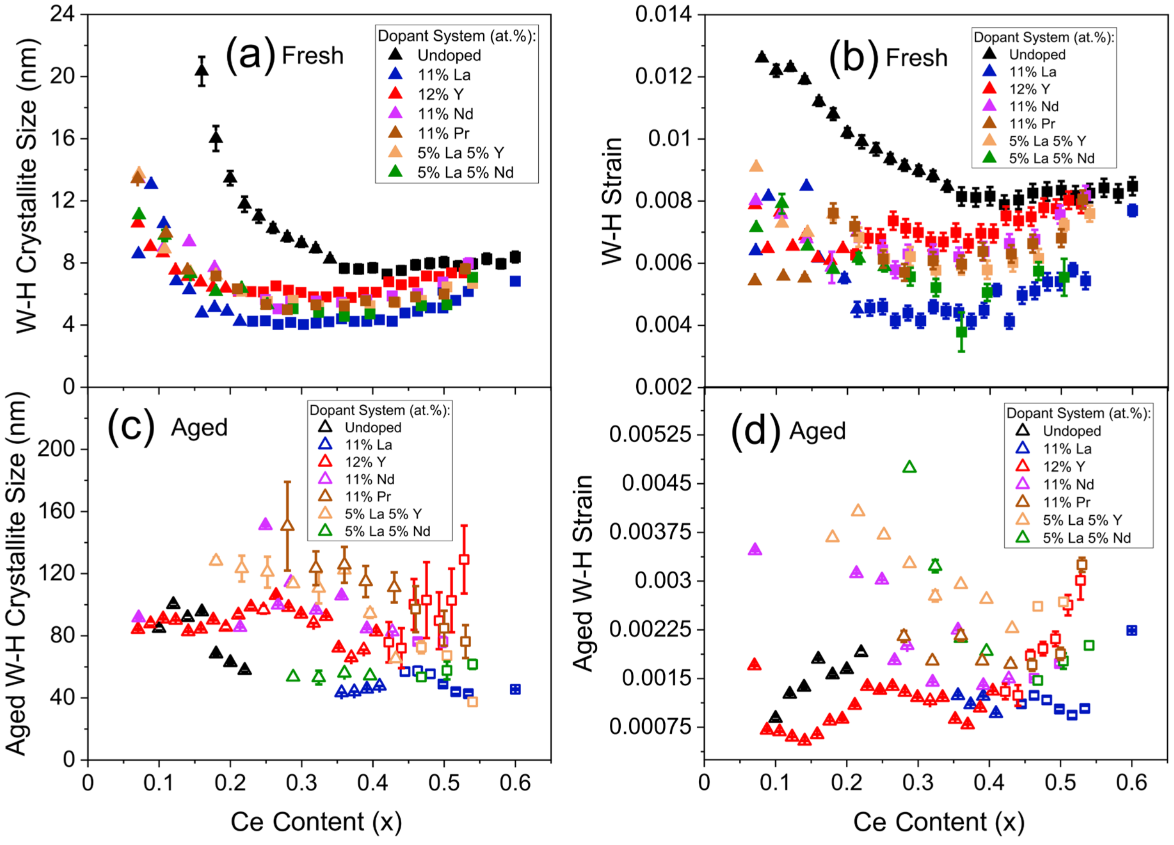

Fig. S18. Williamson-Hall crystallite sizes and strains extracted from PXRD screenings for materials with the general formula $Ce_xZr_{1-x-y}Ln_yO_{2-\delta}$, keeping the stated lanthanide dopant content (y) constant while varying Ce content (x). Williamson-Hall crystallite sizes for (a) fresh and (c) thermally aged materials are given, alongside the Williamson-Hall strain values, for the (b) fresh and (d) thermally aged materials against Ce content, for undoped, single doped (La, Y, Pr and Nd) and mixed dopant systems (La-Y and La-Nd), across $0.00 \leq y \leq 0.12$. The shape of the plotted points indicates the phase of the materials (triangles are tetragonal (t) crystal structures and squares are pseudo-cubic (t'') crystal structures). Values are displayed for materials demonstrating a single phase in the PXRD patterns (Figure S8).

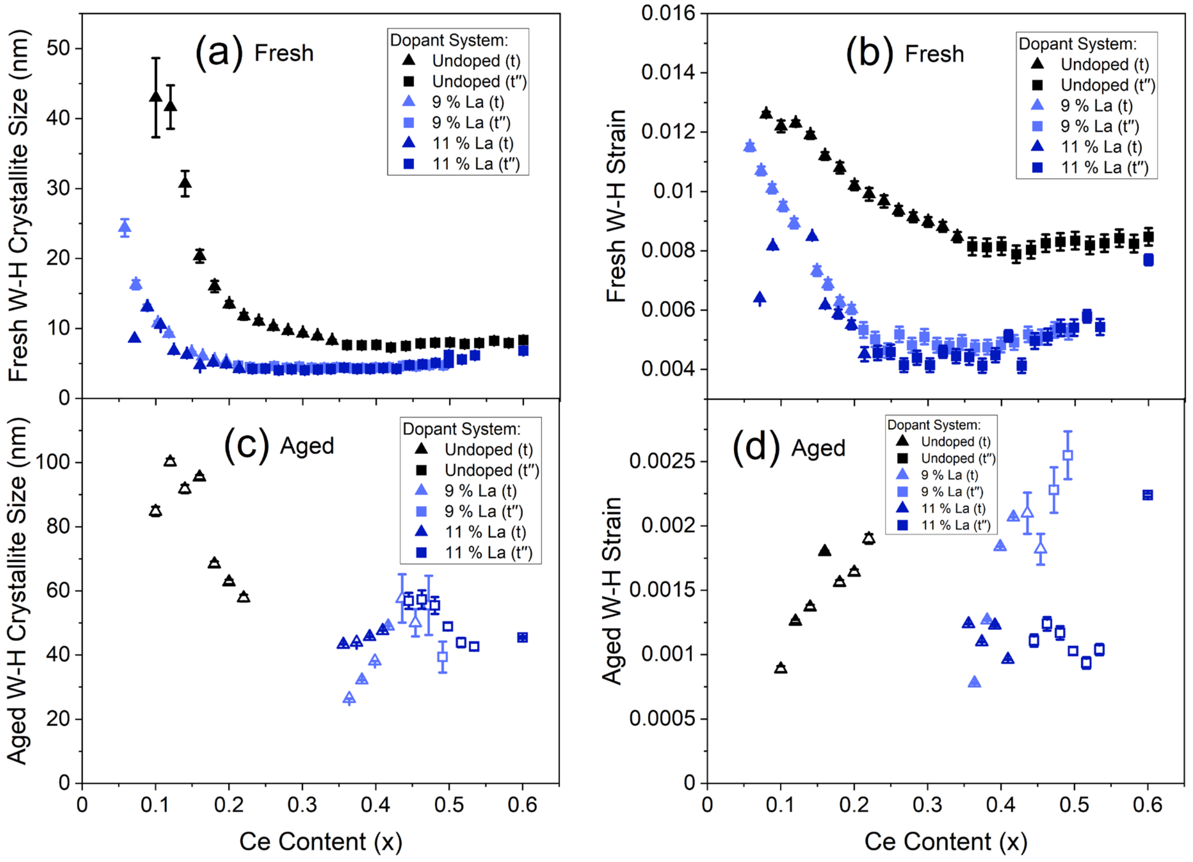

Fig. S19. Williamson-Hall crystallite sizes and strains extracted from PXRD screenings for materials with the general formula $Ce_xZr_{1-x-y}La_yO_{2-\delta}$, keeping the stated lanthanide dopant content (y) constant while varying Ce content (x). Williamson-Hall crystallite sizes for (a) fresh and (c) thermally aged materials are given, alongside the Williamson-Hall strain values, for undoped (y = 0), 9% La (y = 0.09) and 11% La doped (y = 0.11) systems. The shape of the plotted points indicates the phase of the materials (triangles are tetragonal (t) crystal structures and squares are pseudo-cubic (t'') crystal structures). Values are displayed for materials demonstrating a single phase in the PXRD patterns (Figure S9).

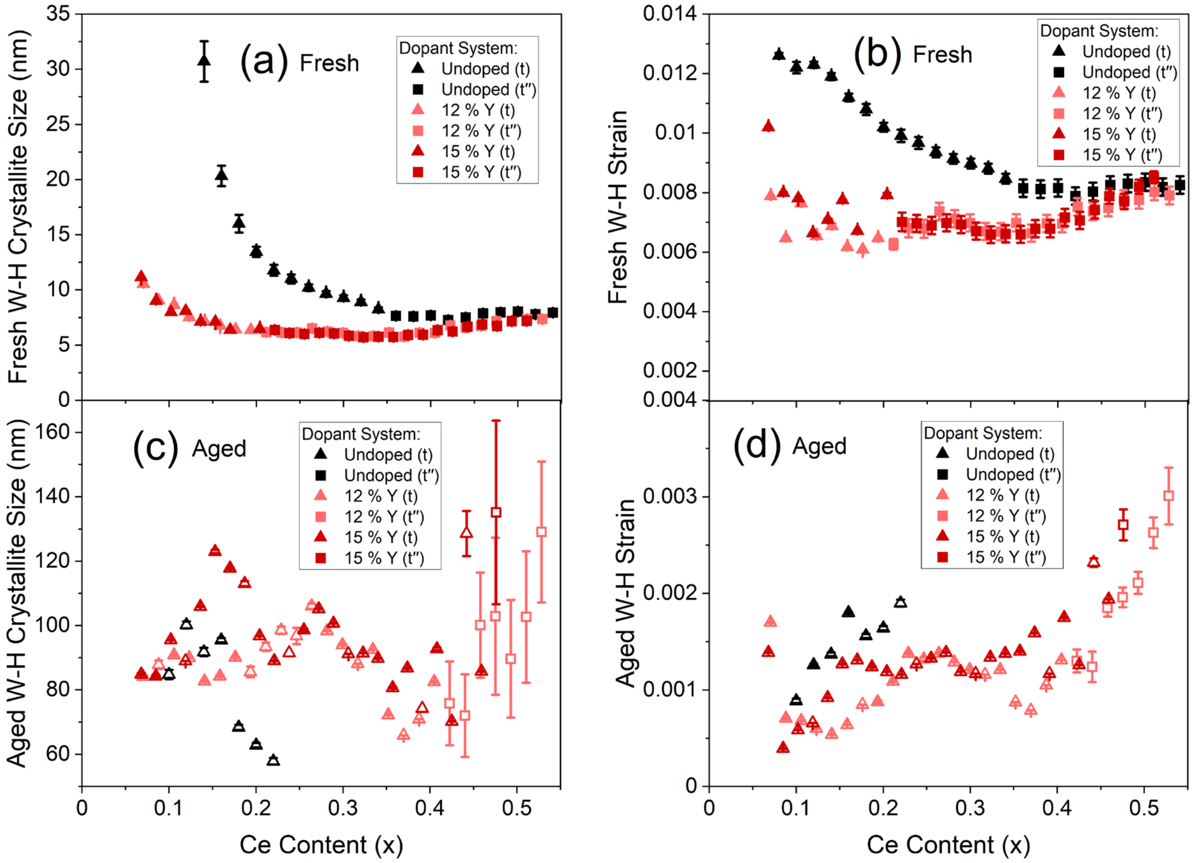

Fig. S20. Williamson-Hall crystallite sizes and strains extracted from PXRD screenings for materials with the general formula $Ce_xZr_{1-x-y}Y_yO_{2-\delta}$, keeping the stated lanthanide dopant content (y) constant while varying Ce content (x). Williamson-Hall crystallite sizes are given for undoped (y = 0), 12% Y (y = 0.12) and 15% Y (y = 0.15) doped systems. The shape of the plotted points indicates the phase of the materials (triangles are tetragonal (t) crystal structures and squares are pseudo-cubic (t'') crystal structures). Values are displayed for materials demonstrating a single phase in the PXRD patterns (Figure S9).

C.7.2. Increasing Dopant Content with a Constant Ce/Zr Ratio of 1

Extracted Williamson-Hall crystallite sizes and strains from PXRD patterns for both fresh and thermally aged materials with the general formula $Ce_{(1-y)/2}Zr_{(1-y)/2}Ln_yO_{2-\delta}$, keeping the Ce/Zr ratio constant at 1 while increasing the stated lanthanide dopant content (y), for single dopant (La, Y, Pr and Nd) and mixed dopant (La-Y and La-Nd) systems.

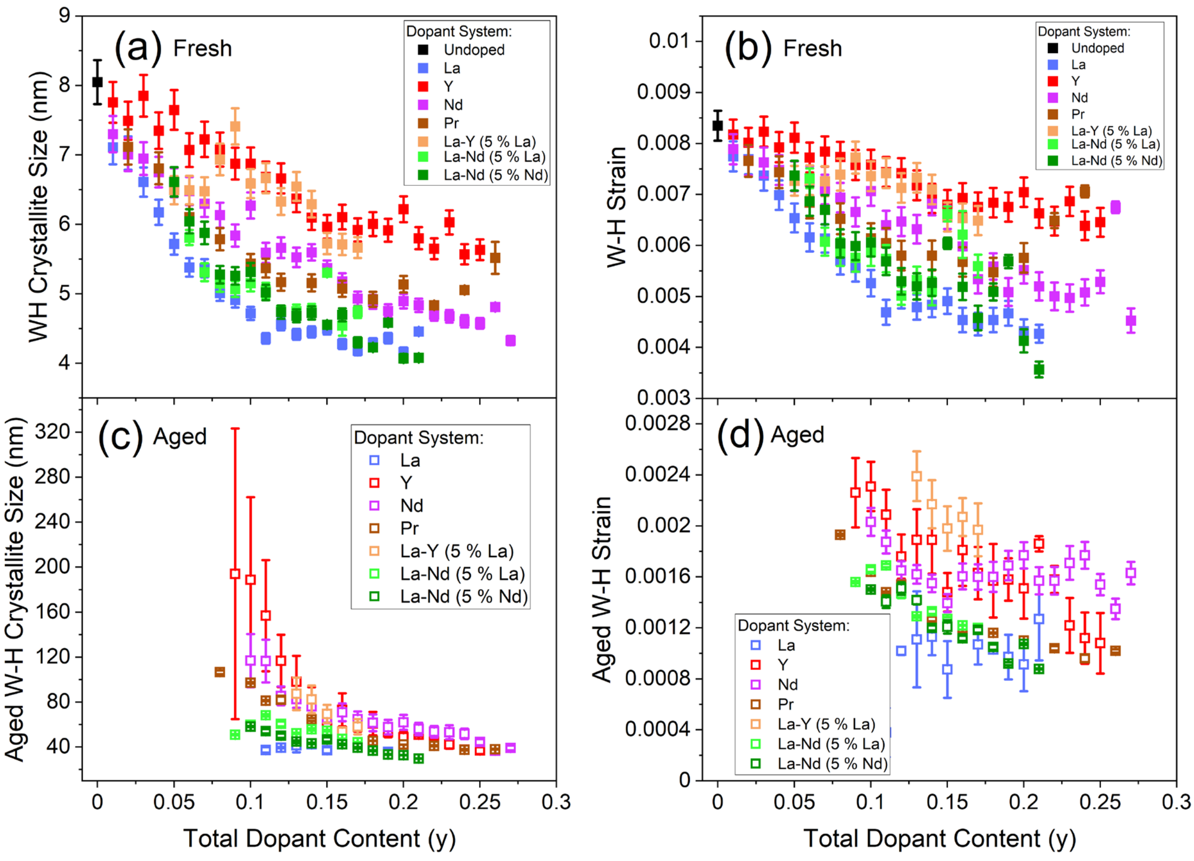

Fig. S21. Williamson-Hall crystallite sizes and strains extracted from PXRD screenings for materials with the general formula $Ce_{(1-y)/2}Zr_{(1-y)/2}Ln_yO_{2-\delta}$, keeping Ce/Zr constant at 1 while increasing the stated lanthanide dopant content (y). Williamson-Hall crystallite sizes for the (a) fresh and (c) thermally aged materials are given, along with the corresponding Williamson-Hall strains for the (b) fresh (d) thermally aged materials, against total dopant content, for undoped, single doped (La, Y, Pr and Nd) and mixed dopant systems (La-Y and La-Nd). Values are displayed for materials demonstrating a single phase in the PXRD patterns (Figure S10).

C.8. TGA Reduction

C.8.1. Varying Ce/Zr Ratio with Constant Dopant Content

The reduction peak temperature (calculated as the temperature of the peak in the derivative TG curve of weight with temperature) and the mass lost during reduction (calculated as the mass lost between the onset and offset temperature of the peak in the differential TG curve) are given for fresh and thermally aged materials, with the general formula $Ce_xZr_{1-x-y}Ln_yO_{2-\delta}$, where the stated lanthanide dopant content (y) is fixed and the Ce content (x) is varied, for undoped, single dopant (La, Y, Pr and Nd), and mixed dopant (La-Y and La-Nd) systems.

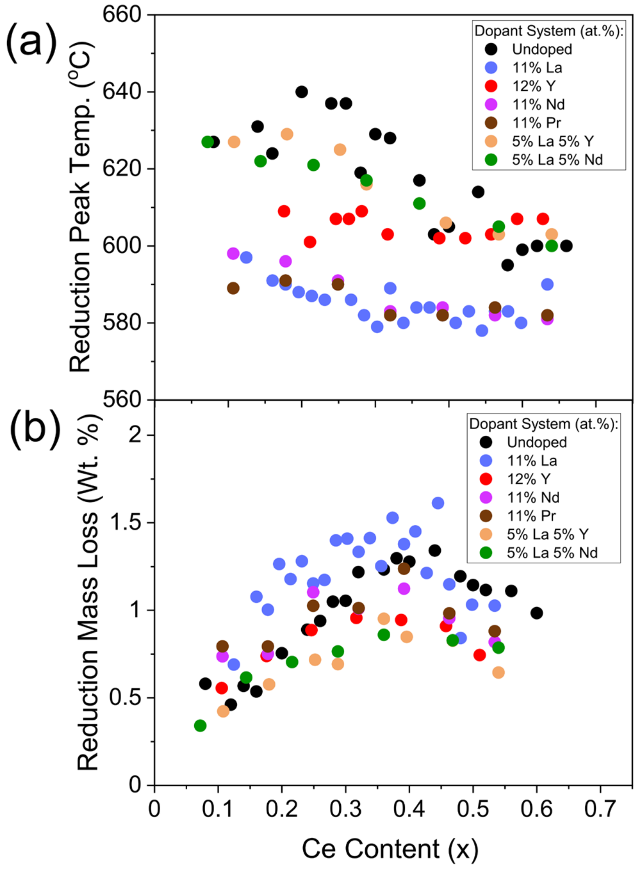

Fig. S22. The evaluation of the (a) reduction peak temperature and (b) mass lost during reduction from TGA measurements against Ce content, for materials with the general formula $Ce_xZr_{1-x-y}Ln_yO_{2-\delta}$, where the stated lanthanide dopant content (y) is fixed and the Ce content (x) is varied between $0.00 < x < 0.65$. Undoped, single doped (La, Y, Pr and Nd) and mixed dopant systems (La-Y and La-Nd) are plot for $0.00 \leq y \leq 0.12$.

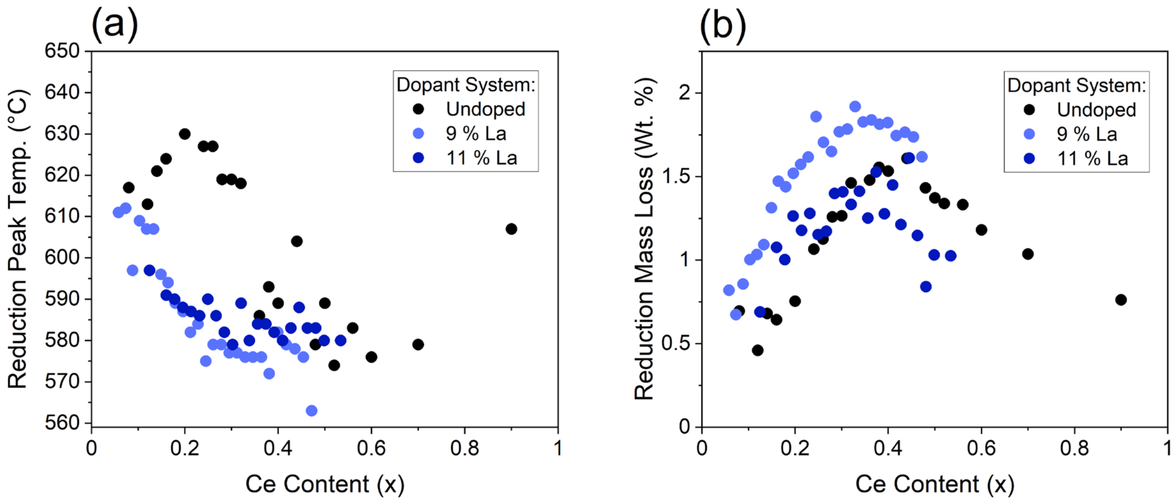

Fig. S23. The evaluation of the (a) reduction peak temperature and (b) mass lost during reduction from TGA measurements against Ce content, for materials with the general formula $Ce_xZr_{1-x-y}La_yO_{2-\delta}$, where the stated lanthanide dopant content (y) is fixed and the Ce content (x) is varied between 0.00 < x < 1. Undoped (y = 0), 9% La (y = 0.09) and 11% La (y = 0.11) systems are plot.

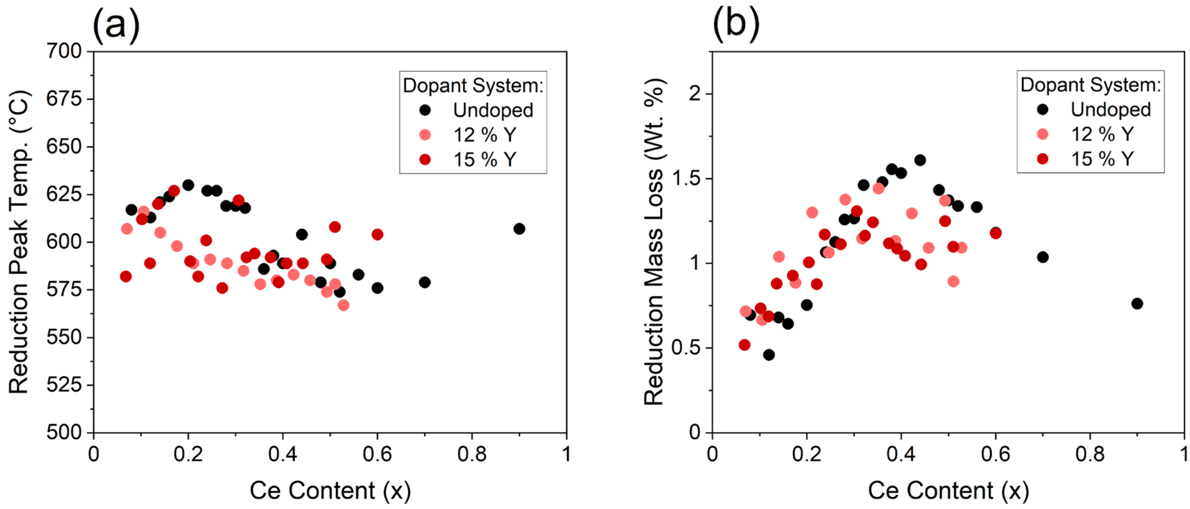

Fig. S24. The evaluation of the (a) reduction peak temperature and (b) mass lost during reduction from TGA measurements against Ce content, for materials with the general formula $Ce_xZr_{1-x-y}Y_yO_{2-\delta}$, where the stated lanthanide dopant content (y) is fixed and the Ce content (x) is varied between 0.00 < x < 1. Undoped (y = 0), 12% Y (y = 0.12) and 15% Y (y = 0.15) systems are plot.

C.8.2. Increasing Dopant Content with a Constant Ce/Zr Ratio of 1

The reduction peak temperature (calculated as the temperature of the peak in the derivative TG curve of weight with temperature) and the mass lost during reduction (calculated as the mass lost between the onset and offset temperature of the peak in the differential TG curve) are given for fresh and thermally aged materials, with the general formula $Ce_{(1-y)/2}Zr_{(1-y)/2}Ln_yO_{2-\delta}$, keeping the Ce/Zr ratio constant at 1 while increasing the stated lanthanide dopant content (y) for single dopant (La, Y, Pr and Nd) and mixed dopant (La-Y and La-Nd) systems.

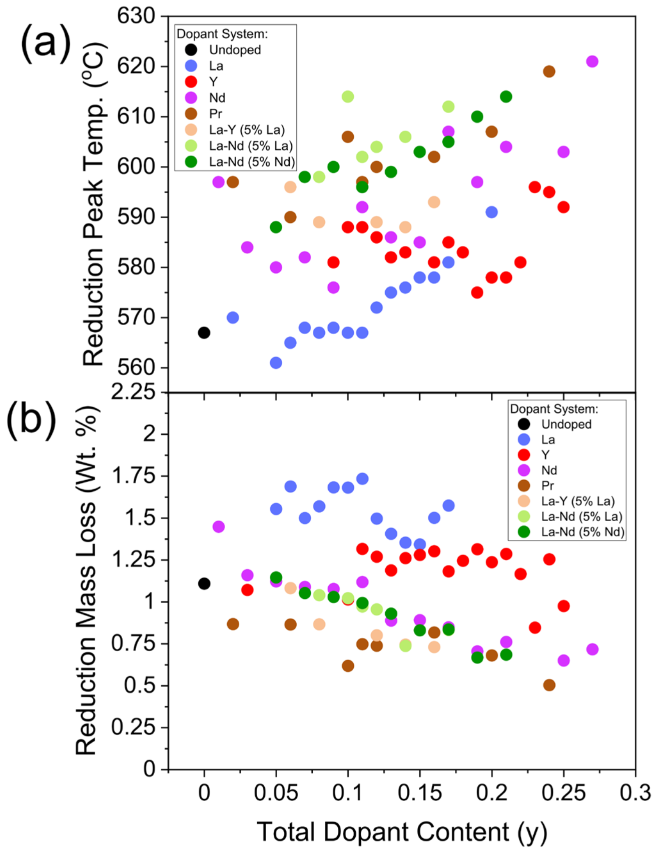

Fig. S25. The evaluation of the (a) reduction peak temperature and (b) mass lost during reduction from TGA measurements against Ce content, for materials with the general formula $Ce_{(1-y)/2}Zr_{(1-y)/2}Ln_yO_{2-\delta}$, keeping Ce/Zr constant at 1 while increasing the stated lanthanide dopant content (y). Undoped, single doped (La, Y, Pr and Nd) and mixed dopant systems (La-Y and La-Nd) are plot.

C.9. Nitrogen Adsorption

C.9.1. Varying Ce/Zr Ratio with Constant Dopant Content

Nitrogen adsorption measurements were taken on fresh materials with the general formula $Ce_xZr_{1-x-y}Ln_yO_{2-\delta}$, where the stated lanthanide dopant content (y) is fixed and the Ce content (x) is varied, for undoped, single dopant (La, Y, Pr and Nd), and mixed dopant (La-Y and La-Nd) systems.

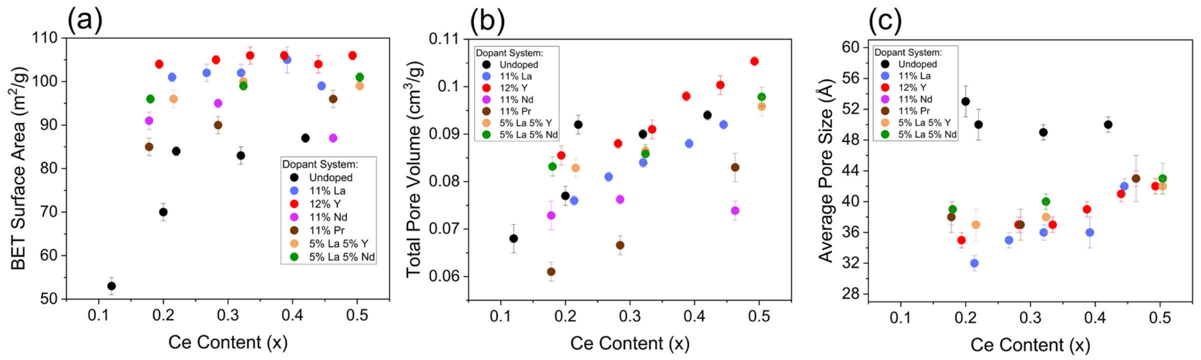

Fig. S26. (a) BET Surface areas, (b) total pore volumes and (c) average pore sizes are given against Ce content for materials with the general formula $Ce_xZr_{1-x-y}Ln_yO_{2-\delta}$, where the stated lanthanide dopant content (y) is fixed and the Ce content (x) is varied between $0.00 < x < 0.60$. Undoped, single doped (La, Y, Pr and Nd) and mixed dopant systems (La-Y and La-Nd) are plot for $0.00 \leq y \leq 0.12$.

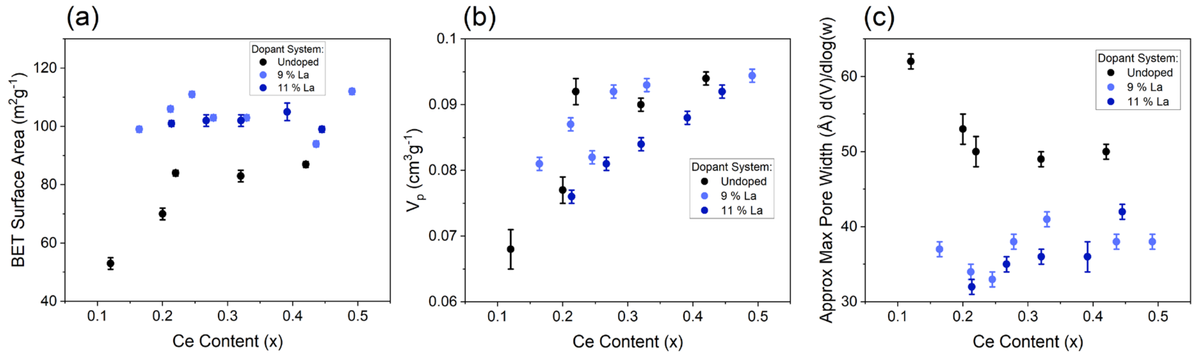

Fig. S27. (a) BET Surface areas, (b) total pore volumes and (c) average pore sizes are given against Ce content for materials with the general formula $Ce_xZr_{1-x-y}La_yO_{2-\delta}$, where the stated lanthanide dopant content (y) is fixed and the Ce content (x) is varied between $0.00 < x < 0.60$. Undoped (y = 0), 9% La (y = 0.09) and 11% La (y = 0.11) systems are plot.

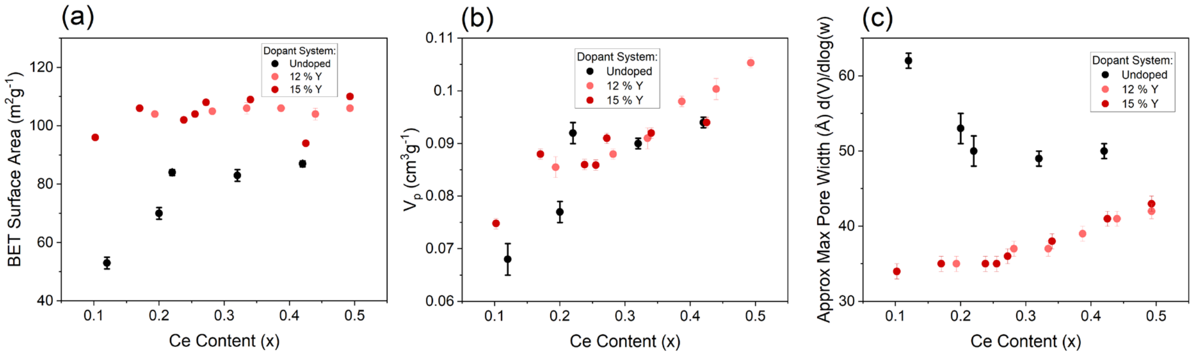

Fig. S28. (a) BET Surface areas, (b) total pore volumes and (c) average pore sizes are given against Ce content for materials with the general formula $Ce_xZr_{1-x-y}Y_yO_{2-\delta}$, where the stated lanthanide dopant content (y) is fixed and the Ce content (x) is varied between $0.00 < x < 0.60$. Undoped (y = 0), 12% Y (y = 0.12) and 15% Y (y = 0.15) systems are plot.

C.9.2. Increasing Dopant Content with a Constant Ce/Zr Ratio of 1

Nitrogen adsorption measurements were taken across the compositions with the general formula $Ce_{(1-y)/2}Zr_{(1-y)/2}Ln_yO_{2-\delta}$, keeping the Ce/Zr ratio constant at 1 while increasing the stated lanthanide dopant content (y) for single dopant (La, Y, Pr and Nd) and mixed dopant (La-Y and La-Nd) systems. The surface areas, total pore volumes and average pore sizes are given below.

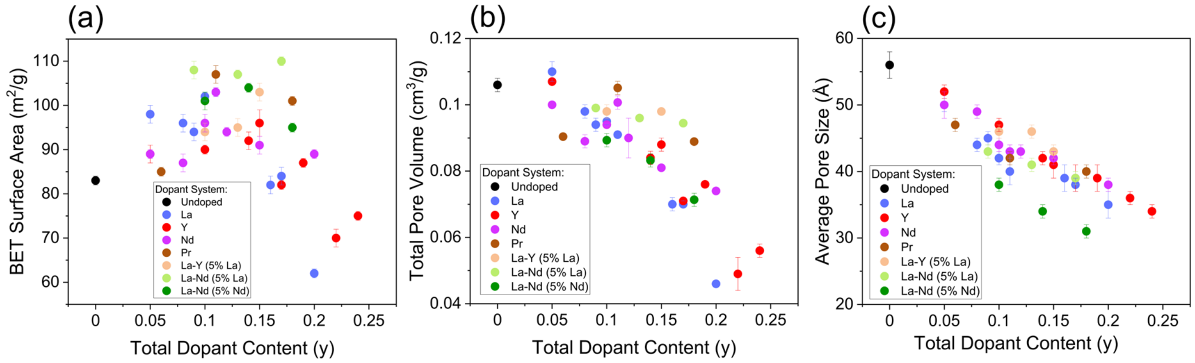

Fig. S29. (a) BET Surface areas, (b) total pore volumes and (c) average pore sizes are given for materials with the general formula $Ce_{(1-y)/2}Zr_{(1-y)/2}Ln_yO_{2-\delta}$, keeping Ce/Zr constant at 1 while increasing the stated lanthanide dopant content (y). Undoped, single doped (La, Y, Pr and Nd) and mixed dopant systems (La-Y and La-Nd) are plot.

D. Prediction Modelling

D.1. 30-sample Training Set

Prediction models for oxygen storage capacity were developed using a set of 30 compositions listed in the table below, for which the OSC was measured.

Table S4. For each of the 30 samples chosen to form the model training set, for which OSC was measured, corresponding values for Ce content, total dopant content, Williamson-Hall crystallite size, mass lost during reduction and OSC are given. The samples are listed in ascending order of Ce content.

Composition	Ce Content (at.%)	Total Dopant Content (at.%)	Williamson-Hall Crystallite Size (nm)	Reduction Mass Loss (Wt.%)	OSC (µmol[O]g^{-1})
$Ce_{0.102}Zr_{0.748}Y_{0.15}O_{2-\delta}$	10.2	15	6.31(15)	0.256(13)	166(8)
$Ce_{0.120}Zr_{0.880}O_{2-\delta}$	12.0	0	42(3)	0.461(23)	274(14)
$Ce_{0.200}Zr_{0.800}O_{2-\delta}$	20.0	0	14(1)	0.755(38)	450(23)
$Ce_{0.255}Zr_{0.595}Y_{0.150}O_{2-\delta}$	25.5	15	5.94(17)	1.034(52)	484(24)
$Ce_{0.264}Zr_{0.616}La_{0.090}Y_{0.030}O_{2-\delta}$	26.4	12	4.50(14)	1.024(51)	482(24)
$Ce_{0.267}Zr_{0.623}Pr_{0.110}O_{2-\delta}$	26.7	11	5.07(12)	1.033(52)	466(23)
$Ce_{0.267}Zr_{0.623}Nd_{0.110}O_{2-\delta}$	26.7	11	5.06(12)	0.900(45)	488(24)
$Ce_{0.278}Zr_{0.632}La_{0.090}O_{2-\delta}$	27.8	9	4.41(16)	1.651(82)	554(28)
$Ce_{0.390}Zr_{0.390}Y_{0.220}O_{2-\delta}$	39.0	22	5.65(15)	1.166(58)	503(25)
$Ce_{0.390}Zr_{0.498}La_{0.110}O_{2-\delta}$	39.2	11	4.27(14)	1.278(64)	586(29)
$Ce_{0.425}Zr_{0.425}La_{0.050}Y_{0.100}O_{2-\delta}$	42.5	15	5.25(13)	1.075(54)	560(28)
$Ce_{0.425}Zr_{0.425}Y_{0.150}O_{2-\delta}$	42.5	15	5.97(17)	1.280(64)	561(28)
$Ce_{0.435}Zr_{0.435}La_{0.050}Nd_{0.080}O_{2-\delta}$	43.5	13	5.50(7)	0.886(44)	478(24)
$Ce_{0.436}Zr_{0.474}La_{0.090}O_{2-\delta}$	43.6	9	4.66(9)	1.765(88)	643(32)
$Ce_{0.440}Zr_{0.440}La_{0.090}Y_{0.030}O_{2-\delta}$	44.0	12	5.23(8)	1.052(53)	589(30)
$Ce_{0.445}Zr_{0.445}Nd_{0.110}O_{2-\delta}$	44.5	11	6.98(13)	1.055(53)	621(31)
$Ce_{0.445}Zr_{0.445}La_{0.110}O_{2-\delta}$	44.5	11	4.36(16)	1.734(87)	626(31)
$Ce_{0.445}Zr_{0.445}Pr_{0.110}O_{2-\delta}$	44.5	11	6.01(11)	1.049(52)	568(28)
$Ce_{0.455}Zr_{0.455}La_{0.090}O_{2-\delta}$	55.5	9	4.97(7)	1.143(57)	646(32)
$Ce_{0.475}Zr_{0.475}Nd_{0.05}O_{2-\delta}$	47.5	5	6.84(23)	1.203(60)	655(33)
$Ce_{0.475}Zr_{0.475}Pr_{0.050}O_{2-\delta}$	47.5	5	6.81(14)	1.200(60)	647(32)
$Ce_{0.491}Zr_{0.419}La_{0.090}O_{2-\delta}$	49.1	9	5.37(13)	1.100(55)	596(30)
$Ce_{0.493}Zr_{0.357}Y_{0.150}O_{2-\delta}$	49.3	15	7.19(20)	1.249(63)	573(29)
$Ce_{0.500}Zr_{0.500}O_{2-\delta}$	50.0	0	7.48(27)	1.331(67)	752(38)
$Ce_{0.560}Zr_{0.440}O_{2-\delta}$	46.0	0	8.14(32)	1.324(66)	706(35)
$Ce_{0.600}Zr_{0.290}La_{0.110}O_{2-\delta}$	60.0	11	6.83(14)	0.976(49)	561(28)
$Ce_{0.600}Zr_{0.250}Y_{0.150}O_{2-\delta}$	60.0	15	7.62(18)	1.178(59)	501(25)
$Ce_{0.700}Zr_{0.300}O_{2-\delta}$	70.0	0	8.48(16)	1.037(52)	606(30)
$Ce_{0.800}Zr_{0.090}La_{0.110}O_{2-\delta}$	80.0	11	8.52(11)	0.673(34)	478(24)
$Ce_{0.900}Zr_{0.100}O_{2-\delta}$	90.0	0	7.97(18)	0.762(38)	454(23)

D.2. Best OSC Prediction Model Statistical Output and Cross-validation

Below is an output from JMP Pro 16 software, giving the statistics output from the multiple linear regression OSC prediction model given in Figure 10 in the main text. The summary of fit, analysis of variance, and parameter estimates are given below. The cross-validation analysis is also given below. A full description of each term in the tables is given in section A4.

Table S5. Summary of fit table for the OSC prediction model given in Figure 10 in the main text. A full description of each term in this table is given in section A.4.2.

**RSquare**	0.951047
**RSquare Adj**	0.938277
**Root Mean Square Error**	29.16121
**Mean of Response**	542.4667
**Observations (or Sum Wgts)**	30

Table S6. Analysis of variance table for the OSC prediction model given in Figure 10 in the main text. A full description of each term in this table is given in section A.4.2.

Source	DF	Sum of Squares	Mean Square	F Ratio
Model	6	379980.81	63330.1	74.4731
Error	23	19558.66	850.4	**Prob > F**
C. Total	29	399539.47		<.0001

Table S7. Parameter estimates table for the coefficients from each term within the expression of the OSC prediction model in Figure 10 in the main text are given. A full description of each term in this table is given in section A.4.2.

Term	Estimate	Std Error	t Ratio	Prob>|t|
**Intercept**	448.93754	41.69753	10.77	<.0001
**Total Dopant Content (at.%)**	-8.236231	1.187422	-6.94	<.0001
**Ce (at.% / 100)**	259.88361	46.53292	5.58	<.0001
**Williamson-Hall Crystallite Size (nm)**	-2.410549	1.103531	-2.18	0.0394
**(Ce (at.% / 100)-0.436)*(Ce (at.% / 100)-0.436)**	-1312.395	218.8799	-6.00	<.0001
**TGA Reduction Mass Loss (Wt.%)**	114.28421	29.03446	3.94	0.0007
**(TGA Reduction Mass Loss (Wt.%)-1.0875)*(TGA Reduction Mass Loss (Wt.%)-1.0875)**	-79.7892	43.55028	-1.83	0.0799

Cross-validation evaluated how well the model. The original 30 sample training set for the full multiple linear regression OSC prediction model in Figure 10 in the main text (compositions listed in Table S4) was split into two parts: a training set and a test set in an 80/20 split (24 training samples and 6 test samples). The samples were randomly split into the training and test sets and a model was then trained on the 24-sample training set, using the same parameters as in the full OSC prediction model. The model built from the randomly selected 24-sample training set was then tested on the 6-sample test set, and this process was repeated for 10 iterations, each time randomly selecting a new training set and test set in an 80/20 split. The resulting plots of predicted and experimental OSC are given below for each iteration of the cross-validation process.

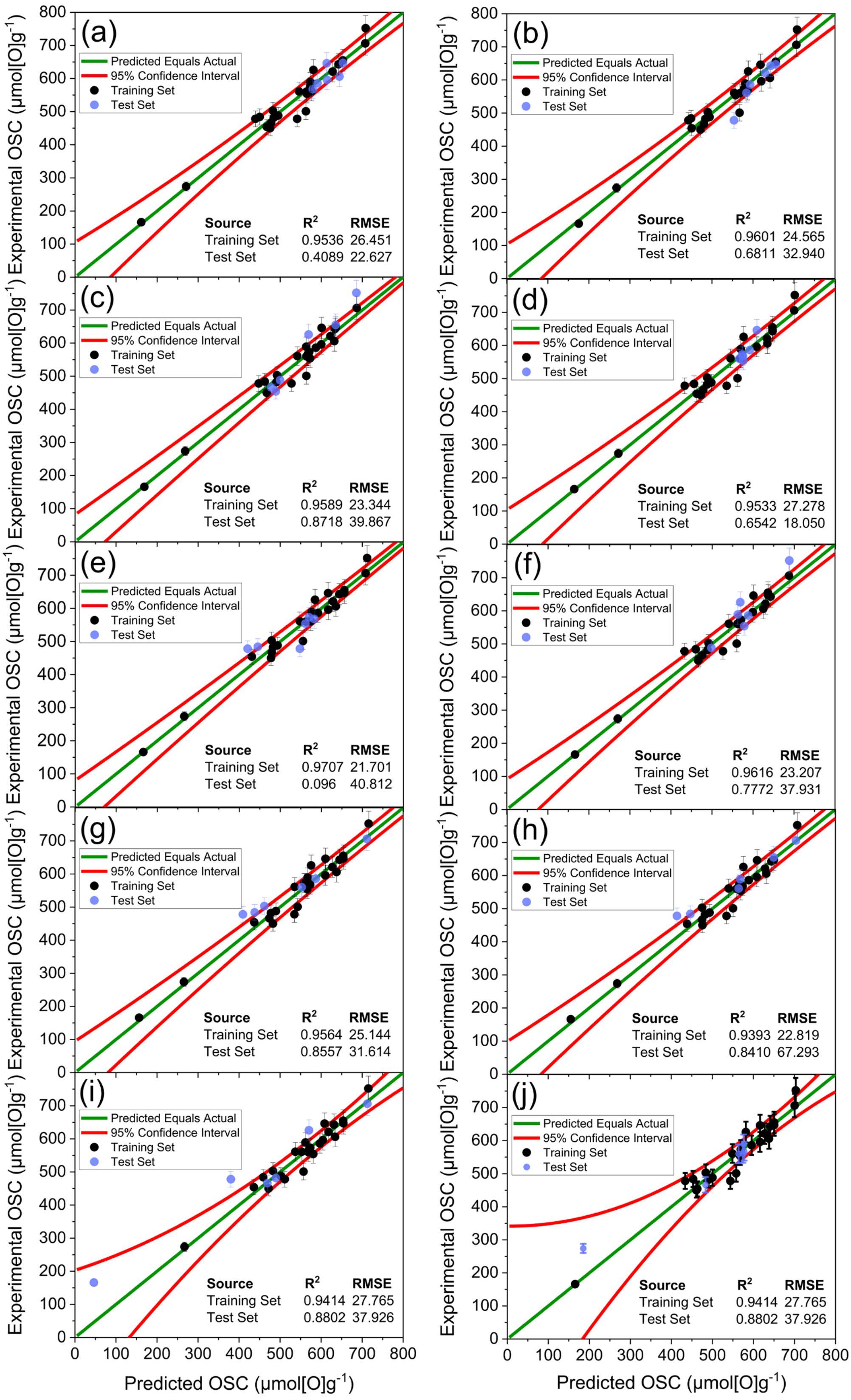

Fig. S30. Cross-validation for the OSC prediction model given in Figure 10 in the main text, using 80% of OSC samples as the training set and 20% as a test set, for (a-j) 10 randomly initialised iterations. A line is given to show predicted equal to actual (green line) along with the 95% confidence interval of the model (red lines). The experimental OSC for the training and test sets are given as black and blue points, respectively, against the predicted values from the model, along with the statistics for each source.

D.3. OSC Prediction Models Containing PXRD Peak Position and Lattice Parameter Variables Over Ce Content

Below is an output from JMP Pro 16 software, giving the multiple linear regression OSC prediction model along with the statistical output for the models containing the PXRD peak position variable or the Fm$\bar{3}$m equivalent lattice parameter variable in place of the Ce content variable incorporated in the OSC prediction model in Figure 10 in the main text. The PXRD peak position variable takes the (111) PXRD peak for cubic and pseudo-cubic materials, and the (011) PXRD peak for tetragonal materials. The summary of fit, analysis of variance, and parameter estimates tables are given below. Below is an output when the peak position variable was incorporated:

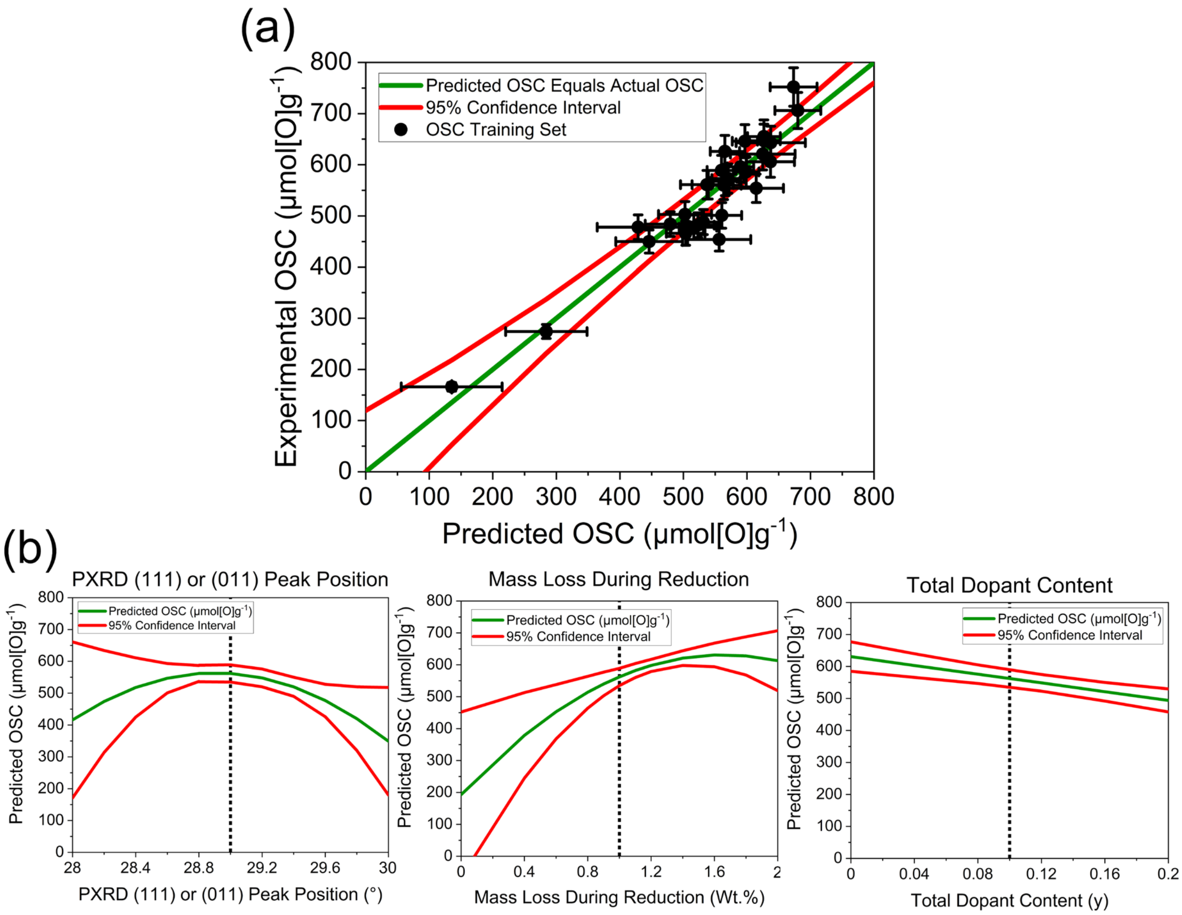

Fig. S31. (a) Predicted OSC against experimentally measured OSC using a multiple linear regression OSC prediction model, containing the PXRD peak position variable over the Ce content variable incorporated in the OSC prediction model in Figure 10 in the main text, for a total of 30 ceria-zirconia materials in the training set (compositions given in Table S4). The general formula of the materials within the training set compositions is $Ce_xZr_{1-x-y}Ln_yO_{2-\delta}$ (where Ln = La, Y, Pr, Nd for 1 dopant systems and Ln = La-Y and La-Nd for 2 dopant systems). The red lines give the 95% confidence interval of the predictions. (b) Profile traces of the parameters within the model (green line), giving the predicted OSC value as the parameter is changed over the full range of the x-axis, while the other parameters are held constant at values indicated by a dotted line in the respective plots. The 95% confidence intervals are also given as red lines.

Table S8. Summary of fit for the OSC prediction model containing the PXRD (111) or (011) peak position variable over the Ce content variable incorporated in the OSC prediction model in Figure 10 in the main text. A full description of each term in this table is given in section A.4.2.

**RSquare**	0.887524
**RSquare Adj**	0.864092
**Root Mean Square Error**	43.27168
**Mean of Response**	542.4667
**Observations (or Sum Wgts)**	30

Table S9. Analysis of variance for the OSC prediction model containing the PXRD (111) or (011) peak position variable over the Ce content variable incorporated in the OSC prediction model in Figure 10 in the main text. A full description of each term in this table is given in section A.4.2.

Source	DF	Sum of Squares	Mean Square	F Ratio
**Model**	5	354600.95	70920.2	37.8758
**Error**	24	44938.52	1872.4	**Prob > F**
**C. Total**	29	399539.47		<.0001

Table S10. Parameter estimates for the coefficients of each term within the expression of the multiple linear regression OSC prediction model containing the PXRD (111) or (011) peak position variable over Ce content variable incorporated in the OSC prediction model in Figure 10 in the main text. A full description of each term in this table is given in section A.4.2.

Term	Estimate	Std Error	t Ratio	Prob>|t|
**Intercept**	2491.0067	812.1566	3.07	0.0053
**TGA Reduction Mass Loss (Wt.%)**	181.69242	48.54493	3.74	0.0010
**Total Dopant Content (at.%)**	-6.829237	1.529928	-4.46	0.0002
**PXRD Peak Position**	-70.31211	27.42765	-2.56	0.0170
**(TGA Reduction Mass Loss (Wt.%)-1.0875)*(TGA Reduction Mass Loss (Wt.%)-1.0875)**	-158.8958	70.9887	-2.24	0.0347
**(PXRD Peak Position-29.102)*(PXRD Peak Position-29.102)**	-180.2202	104.6868	-1.72	0.0980

Below is the statistical output when the $Fm\bar{3}m$ equivalent lattice parameter variable is incorporated over the Ce content variable in the OSC prediction model in Figure 10 in the main text. The summary of fit, analysis of variance, and parameter estimates are given below.

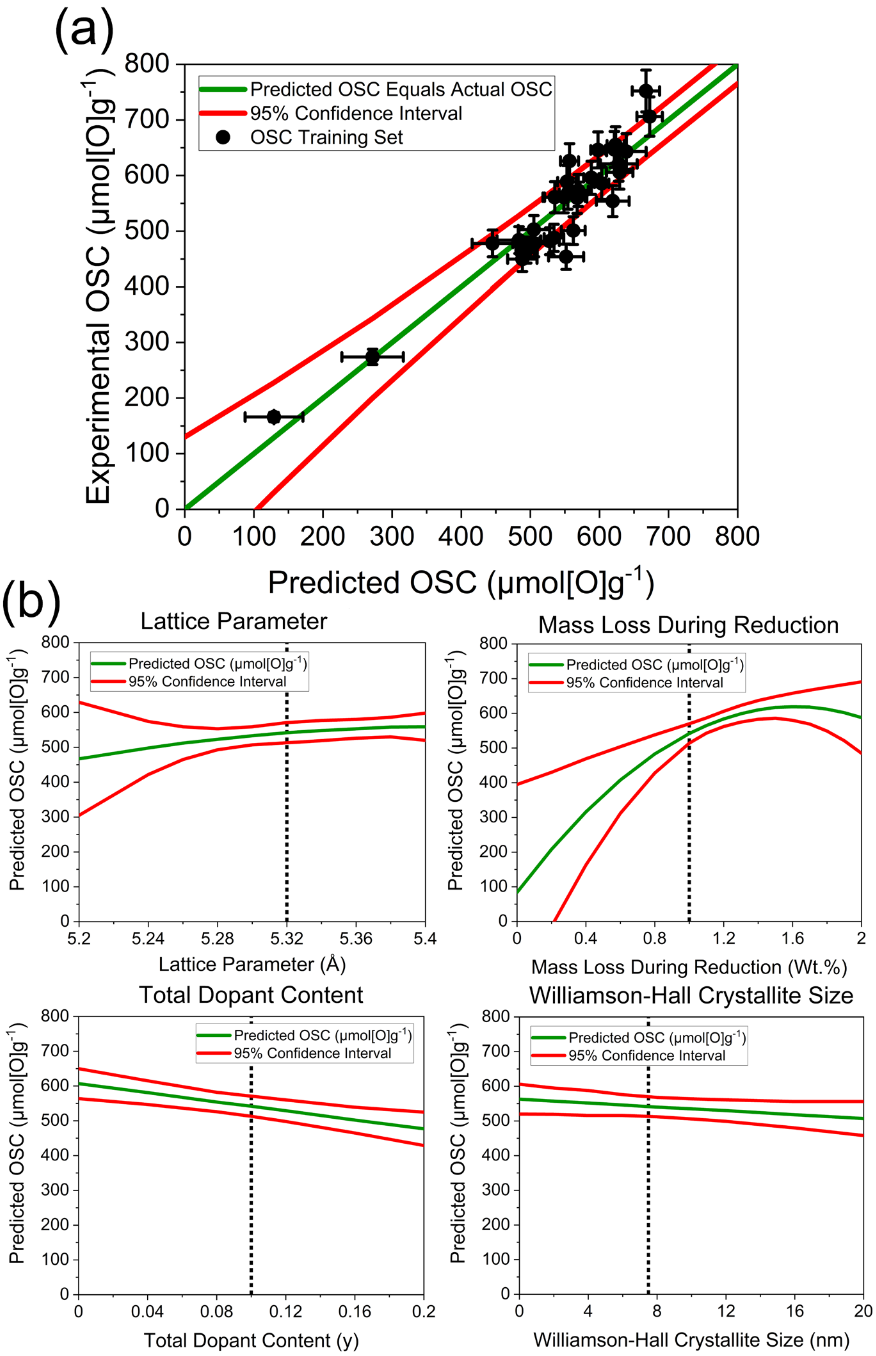

Fig. S32. (a) Predicted OSC against experimentally measured OSC using a multiple linear regression OSC prediction model containing the $Fm\bar{3}m$ equivalent lattice parameter variable over the Ce content variable incorporated in the OSC prediction model in Figure 10 in the main text, for a total of 30 ceria-zirconia materials in the training set (compositions given in Table S4). The general formula of the materials within the training set compositions is $Ce_xZr_{1-x-y}Ln_yO_{2-\delta}$ (where Ln = La, Y, Pr, Nd for 1 dopant systems and Ln = La-Y and La-Nd for 2 dopant systems). The red lines give the 95% confidence interval of the predictions. (b) Profile traces of the parameters within the model (green line), giving the predicted OSC value as the parameter is changed over the full range of the x-axis, while the other parameters are held constant at values indicated by a dotted line in the respective plots. The 95% confidence intervals are also given as red lines.

Table S11. Summary of fit for the OSC prediction model containing the Fm$\bar{3}$m equivalent lattice parameter variable over the Ce content variable incorporated in the OSC prediction model in Figure 10 in the main text. A full description of each term in this table is given in section A.4.2.

**RSquare**	0.880583
**RSquare Adj**	0.849431
**Root Mean Square Error**	45.54583
**Mean of Response**	542.4667
**Observations (or Sum Wgts)**	30

Table S12. Analysis of variance for the OSC prediction model containing the Fm$\bar{3}$m equivalent lattice parameter variable over the Ce content variable incorporated in the OSC prediction model in Figure 10 in the main text. A full description of each term in this table is given in section A.4.2.

<table>
<tr><th>Source</th><th>DF</th><th>Sum of Squares</th><th>Mean Square</th><th>F Ratio</th></tr>
<tr><td>Model</td><td>6</td><td>351827.75</td><td>58638.0</td><td>28.2671</td></tr>
<tr><td>Error</td><td>23</td><td>47711.72</td><td>2074.4</td><td>Prob > F</td></tr>
<tr><td>C. Total</td><td>29</td><td>399539.47</td><td></td><td><.0001</td></tr>
</table>

Table S13. Parameter estimates for the coefficients of each term within the expression of the OSC prediction model containing the Fm$\bar{3}$m equivalent lattice parameter variable over the Ce content variable incorporated in the OSC prediction model in Figure 10 in the main text. A full description of each term in this table is given in section A.4.2.

Term	Estimate	Std Error	t Ratio	Prob>|t|
**Intercept**	-1466.604	1038.781	-1.41	0.1714
**Williamson-Hall Crystallite Size (nm)**	-2.756071	1.71662	-1.61	0.1220
**Lattice Parameter (Å)**	353.37682	197.8489	1.79	0.0873
**TGA Reduction Mass Loss (Wt.%)**	216.26755	54.75891	3.95	0.0006
**Total Dopant Content (at.%)**	-6.536352	1.688582	-3.87	0.0008
**(Lattice Parameter (Å)-5.32717)*(Lattice Parameter (Å)-5.32717)**	-2046.86	4644.502	-0.44	0.6635
**(TGA Reduction Mass Loss (Wt.%)-1.0875)*(TGA Reduction Mass Loss (Wt.%)-1.0875)**	-205.9467	84.70692	-2.43	0.0232

Table S14. Parameter estimates for the coefficients of each term within the expression of the OSC prediction model containing the Fm$\bar{3}$m equivalent lattice parameter variable over the Ce content variable incorporated in the OSC prediction model in Figure 10 in the main text, after removing the quadratic TGA mass loss term. A full description of each term in this table is given in section A.4.2.

Term	Estimate	Std Error	t Ratio	Prob>|t|
**Intercept**	-2514.167	1037.42	-2.42	0.0233
**Williamson-Hall Crystallite Size (nm)**	-3.987316	1.800236	-2.21	0.0365
**Lattice Parameter (Å)**	570.09139	193.8641	2.94	0.0071
**TGA Reduction Mass Loss (Wt.%)**	126.10689	44.22145	2.85	0.0088
**Total Dopant Content (at.%)**	-6.286906	1.84989	-3.40	0.0024
**(Lattice Parameter (Å)-5.32717)*(Lattice Parameter (Å)-5.32717)**	-10780.7	3231.177	-3.34	0.0028

When the PXRD peak position variable was incorporated in the OSC prediction model in place of the Ce content variable, the predicted against actual OSC shows small residuals (Fig. S31). The Williamson-Hall crystallite size variable also demonstrated insignificance and was thus omitted from the model. However, when looking at the parameter estimates output in Table S10, the quadratic parameter of the peak position variable shows a weak significance with a p-value of 0.098.

The $Fm\bar{3}m$ equivalent lattice parameter variable was also trialled in place of the Ce content in the OSC models. Although the predicted against actual OSC of the model containing the lattice parameter variable in Figure S32 shows small residuals, from the statistical output of the parameter estimates in Table S13, the quadratic Lattice parameter shows insignificant with a p-value of 0.6635 and thus should be removed from the model to improve predictions.

Table S14 shows the parameter estimates after the removal of the quadratic mass loss reduction parameter for the model with the lattice parameter variable. Here, the p-value associated with the quadratic parameter of the lattice parameter variable now demonstrates significance, at 0.0028, compared to the p-value of 0.6635 when incorporated alongside a quadratic reduction mass loss parameter (Table S13). This highlights how the lattice parameter and mass loss during reduction variables are covering the same variance in the OSC of the materials, with the mass loss showing slightly a larger significance (Table S13), thus the lattice parameter variable would be removed during model development.

When the incorporation of the Ce content, peak position and lattice parameter variable are compared to each other, the Ce content is the clear choice, with a p-value giving the probability that the parameter estimate is zero for the quadratic parameter of <.0001 (Table S7), compared to the 0.098 and 0.6635 for the peak position and lattice parameter variables, respectively.

D.4. Models Containing Nitrogen Adsorption Variables

Table S15. Summary of fit for the incorporation of the nitrogen adsorption variables into the OSC prediction model given in Figure 10 in the main text. A full description of each term in this table is given in section A.4.2.

**RSquare**	0.953821
**RSquare Adj**	0.933041
**Root Mean Square Error**	30.3729
**Mean of Response**	542.4667
**Observations (or Sum Wgts)**	30

Table S16. Analysis of variance for the incorporation of the nitrogen adsorption variables into the OSC prediction model given in Figure 10 in the main text. A full description of each term in this table is given in section A.4.2.

Source	DF	Sum of Squares	Mean Square	F Ratio
**Model**	9	381089.20	42343.2	45.8999
**Error**	20	18450.27	922.5	**Prob > F**
**C. Total**	29	399539.47		<.0001

Table S17. Parameter estimates for the coefficients of each term within the expression of the incorporation of nitrogen adsorption variables in the OSC prediction model in Figure 10 in the main text. A full description of each term in this table is given in section A.4.2.

Term	Estimate	Std Error	t Ratio	Prob>|t|
**Intercept**	652.84573	239.64	2.72	0.0131
**Ce (at.% / 100)**	260.305	75.22398	3.46	0.0025
**Total Dopant Content (at.%)**	-8.114559	1.58388	-5.12	<.0001
**TGA Reduction Mass Loss (wt.%)**	102.86071	32.69112	3.15	0.0051
**Williamson-Hall Crystallite Size (nm)**	-2.315249	1.7389	-1.33	0.1980
**(Ce (at.% / 100)-0.436)*(Ce (at.% / 100)-0.436)**	-1459.435	274.9226	-5.31	<.0001
**(TGA Reduction Mass Loss (wt.%)-1.0875)*(TGA Reduction Mass Loss (wt.%)-1.0875)**	-78.98561	46.18186	-1.71	0.1027
**BET Surface Area (m^2/g)**	-2.126035	2.64239	-0.80	0.4305
**Total Pore Volume, V_p (cm^3/g)**	1403.1037	2380.661	0.59	0.5622
**Average Pore Size (Å)**	-2.979733	4.616284	-0.65	0.5260

Since the p-values for each of the nitrogen adsorption parameters show insignificance, giving the probability that the parameter has no effect on the response (measured OSC), at 0.43, 0.56 and 0.526, the nitrogen adsorption parameters are removed during the stepwise model development. They are removed one by one since they remain at high p-values after the removal of each one, in any order of removal.

D.5. Ce Content, PXRD Peak Position and Lattice Parameter Correlations

Table S18. Correlation coefficients summarising the strength of the linear relationships between the pairs of variables are given, calculated by the Pairwise method, between Ce content (x), Fm$\bar{3}$m equivalent lattice parameter and PXRD (111) or (011) Peak position (from cubic/pseudo-cubic and tetragonal structures, respectively) variables, when taking the quantities from the 30 samples measured for OSC (listed in Table S4).

	**Ce Content (x)**	**Lattice Parameter (Å)**	**PXRD Peak Position (°)**
**Ce Content (x)**	1	0.92	-0.92
**Lattice Parameter (Å)**	0.92	1	-0.97
**PXRD Peak Position (°)**	-0.92	-0.97	1

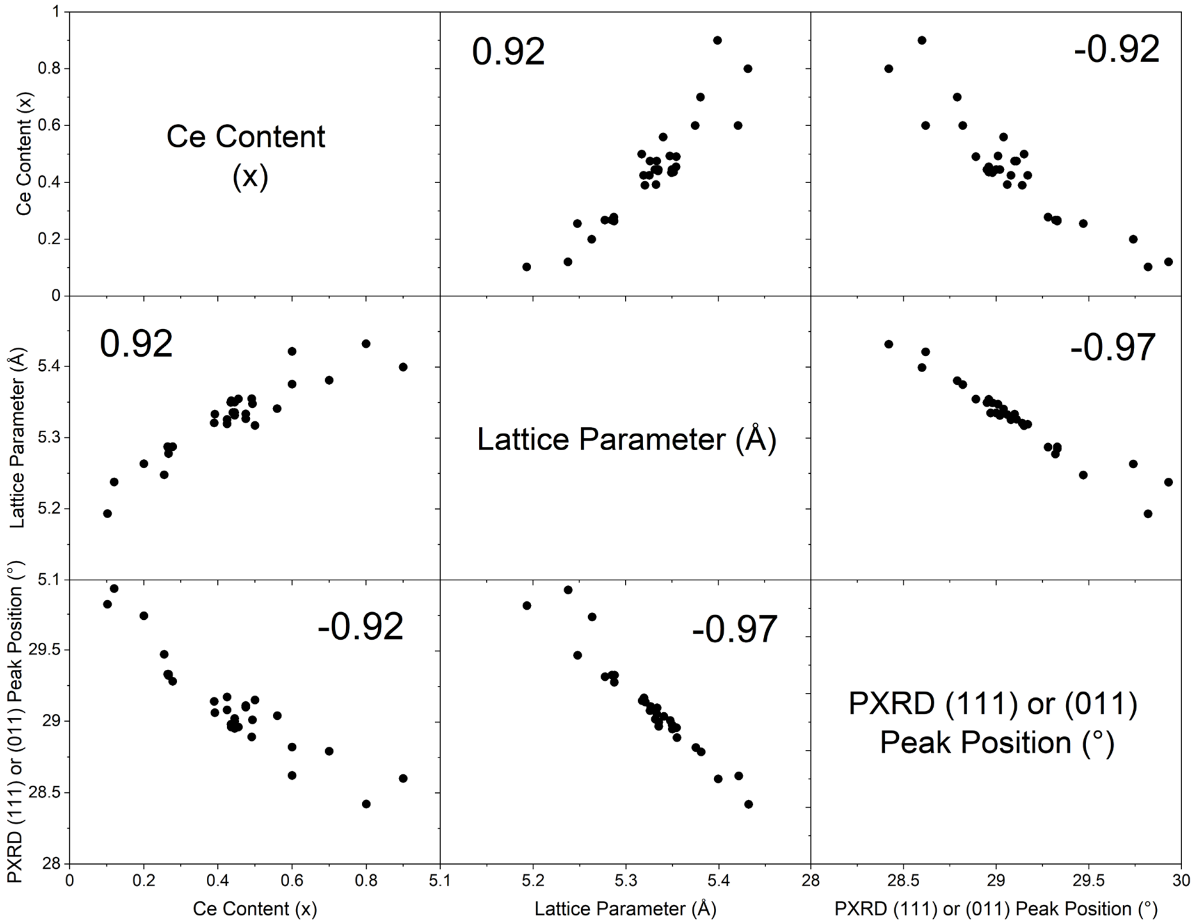

Fig. S33. Scatterplot matrix giving the plots between Ce content, Fm$\bar{3}$m equivalent lattice parameter and PXRD (111) or (011) Peak position (from cubic/pseudo-cubic and tetragonal structures, respectively) variables, when taking the quantities from the 30 samples measured for OSC (listed in Table S4). The compositions have the general formula $Ce_xZr_{1-x-y}Ln_yO_{2-\delta}$ (where Ln = La, Y, Pr, Nd for single dopant systems or Ln = La-Y and La-Nd for 2 dopant systems). The correlation coefficients summarising the strength of the linear relationships between the pairs of variables calculated by the Pairwise method is given on each plot.

D.6. Williamson-Hall Crystallite Size Correlation to Surface Area

Below is a correlation between Williamson-Hall crystallite size, from PXRD measurements, and BET surface area, from N_2 adsorption measurements.

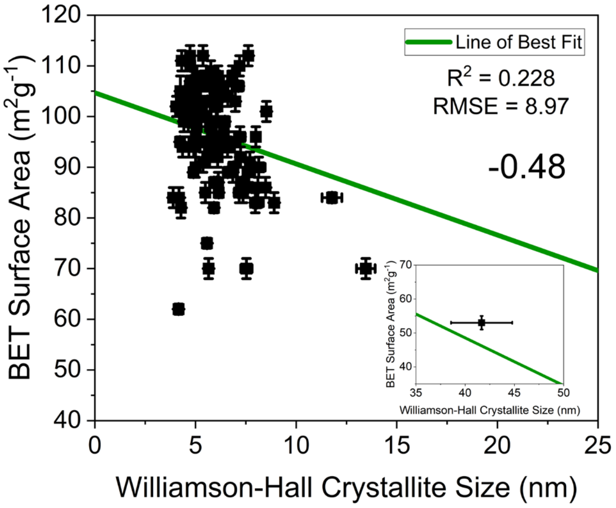

Fig. S34. BET surface area against Williamson-Hall crystallite size for samples screened in this study. Best fit line is given (green line) along with R^2 and RMSE values. These variables have a correlation coefficient of -0.48, estimated by the Pairwise method, which is a moderately strong correlation.

D.7. Scherrer Crystallite Size Inclusion Within OSC Prediction Model

As part of the development of the OSC prediction model discussed in Figure 10 in the main text, the incorporation of the Scherrer crystallite size was compared to the Williamson-Hall crystallite size, alongside Ce content, total dopant content and mass loss during reduction variables, to determine which of the two similar variables shows the most significance towards OSC predictions.

Below is the OSC prediction model produced when the Scherrer crystallite size variable was used alongside the dopant content, mass loss during reduction and Ce content variables in a multiple linear regression OSC prediction model.

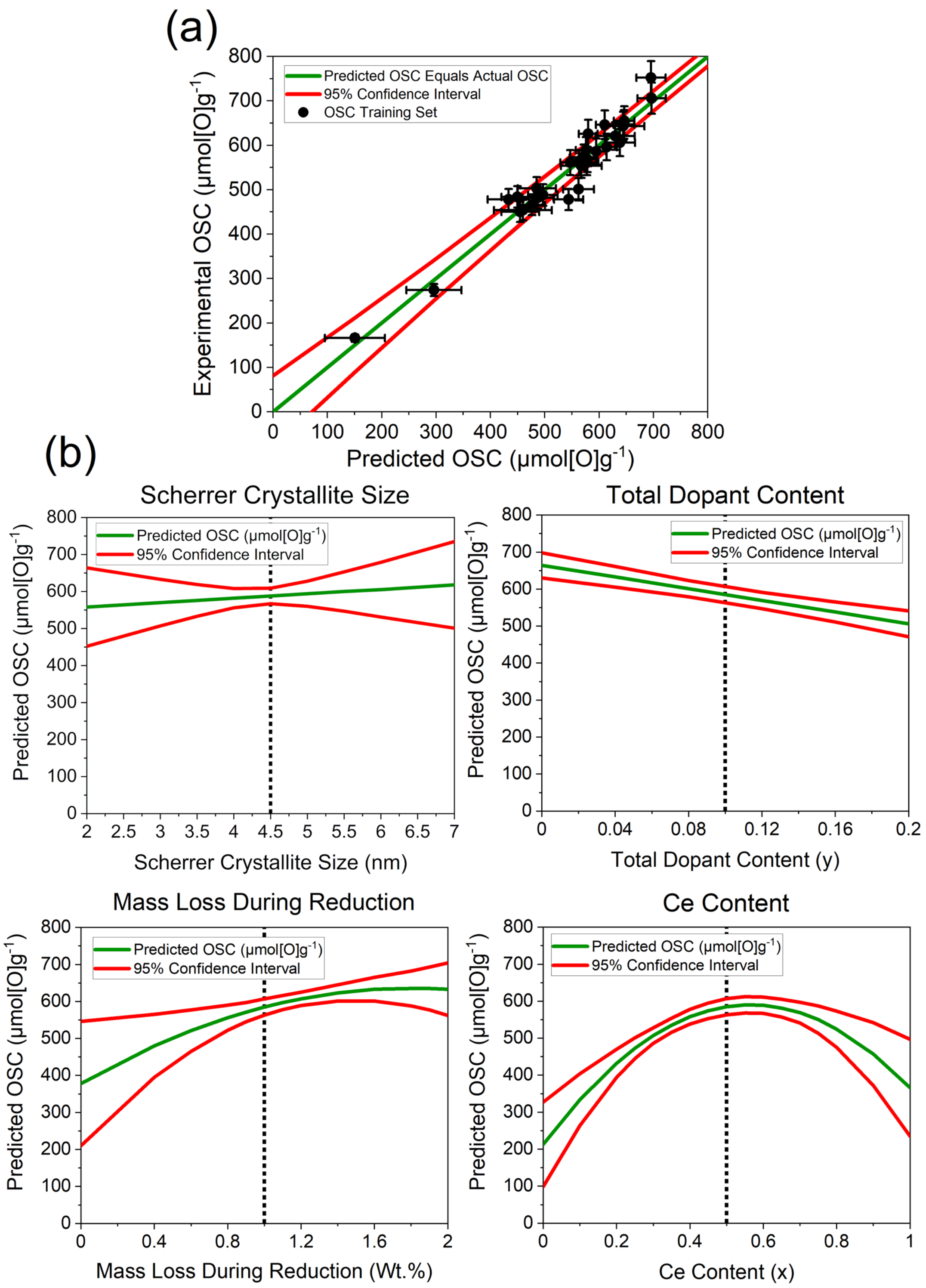

Fig. S35. (a) Predicted OSC against experimentally measured OSC using a multiple linear regression model incorporating dopant content, Ce content, TGA reduction mass loss and Scherrer crystallite size variables for a total of 30 ceria-zirconia materials in the training set (compositions given in Table S4). The general formula of the training set compositions is $Ce_xZr_{1-x-y}Ln_yO_{2-\delta}$ (where Ln = La, Y, Pr, Nd for 1 dopant systems or Ln = La-Y and La-Nd for 2 dopant systems). The red lines give the 95% confidence interval of the predictions. (b) Profile traces of the parameters within the model (green line), giving the predicted OSC value as the parameter is changed over the full range of the x-axis, while the other parameters are held constant at values indicated by a dotted line in the respective plots. The 95% confidence intervals are also given as red lines.

Table S19. Effect summary for the incorporation dopant content, Ce content, TGA reduction mass loss and Scherrer crystallite size variables into an OSC prediction model given in Figure S35. A full description of each term in this table is given in section A.4.2.

Source	PValue
**Total Dopant Content (at.%)**	0.00000
**Ce (at.% / 100)**	0.00000
**Ce (at.% / 100)*Ce (at.% / 100)**	0.00008
**TGA Reduction Mass Loss (wt.%)**	0.00198
**TGA Reduction Mass Loss (wt.%)*TGA Reduction Mass Loss (wt.%)**	0.09792
**Scherrer Crystallite Size (nm)**	0.17794

Table S20. Summary of fit for the incorporation dopant content, Ce content, TGA reduction mass loss and Scherrer crystallite size variables into an OSC prediction model given in Figure S35. Each term is defined in section A.4.2.

**RSquare**	0.94547
**RSquare Adj**	0.931244
**Root Mean Square Error**	30.7776
**Mean of Response**	542.4667
**Observations (or Sum Wgts)**	30

Table S21. Analysis of variance for the incorporation dopant content, Ce content, TGA reduction mass loss and Scherrer crystallite size variables into an OSC prediction model given in Figure S35. A full description of each term in this table is given in section A.4.2.

Source	DF	Sum of Squares	Mean Square	F Ratio
**Model**	6	377752.48	62958.7	66.4640
**Error**	23	21786.99	947.3	**Prob > F**
**C. Total**	29	399539.47		<.0001

Table S22. Parameter estimates for each term in the model expression, incorporating dopant content, Ce content, TGA reduction mass loss and Scherrer crystallite size variables (Figure S35). A full description of each term in this table is given in section A.4.2.

Term	Estimate	Std Error	t Ratio	Prob>|t|
**Intercept**	481.92776	74.39831	6.48	<.0001
**Ce (at.% / 100)**	304.45216	50.53781	6.02	<.0001
**Total Dopant Content (at.%)**	-7.923313	1.293718	-6.12	<.0001
**TGA Reduction mass loss (wt.%)**	113.42175	32.51187	3.49	0.0020
**(Ce (at.% / 100)-0.436)*(Ce (at.% / 100)-0.436)**	-1181.331	248.1578	-4.76	<.0001
**(TGA Reduction mass loss (wt.%)-1.0875)*(TGA Reduction mass loss (wt.%)-1.0875)**	-80.01546	46.38215	-1.73	0.0979
**Scherrer Crystallite Size (nm)**	-17.28715	12.43982	-1.39	0.1779

D.8. Williamson-Hall Crystallite Size Correlation to Scherrer Crystallite Size

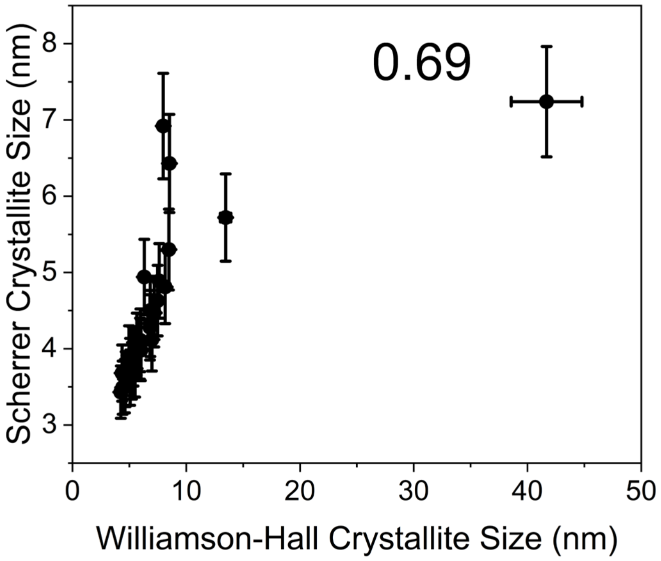

Fig. S36. Plot of the Scherrer Crystallite size against Williamson-Hall crystallite size for the 30 samples in the OSC test set of the OSC prediction model in Figure 10 in the main text (compositions given in Table S4). The correlation coefficient is given as estimated by the Pairwise method.

D.9. Total Dopant Content Correlation to OSC

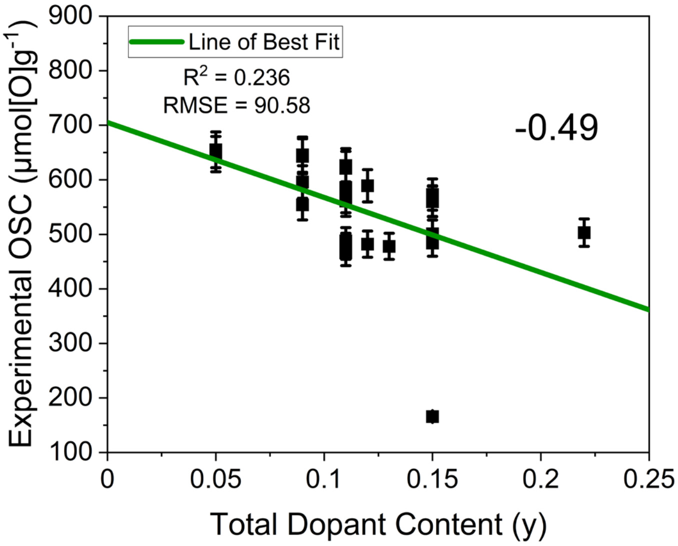

Fig. S37. Experimental OSC against total lanthanide dopant content (y) for the doped samples in the OSC training set of the OSC prediction model in Figure 10 in the main text (compositions given in Table S4), with the general formula $Ce_xZr_{1-x-y}Ln_yO_{2-\delta}$. The line of best fit is given (green line) as well as the R^2 and RMSE. The correlation coefficient is also given, as estimated by the Pairwise method.

D.10. PXRD-only and TGA-only OSC Prediction Models

Before finalising on what would be the statistically optimal OSC prediction model, discussed in the main text, PXRD-only and TGA-only models were investigated using the same 30-sample training set listed in Table S4. Figure S38 gives the PXRD-only model which contains both the Williamson-Hall crystallite size and PXRD peak position variables, and Figure S39 gives the TGA-only model containing only the mass lost during reduction variable.

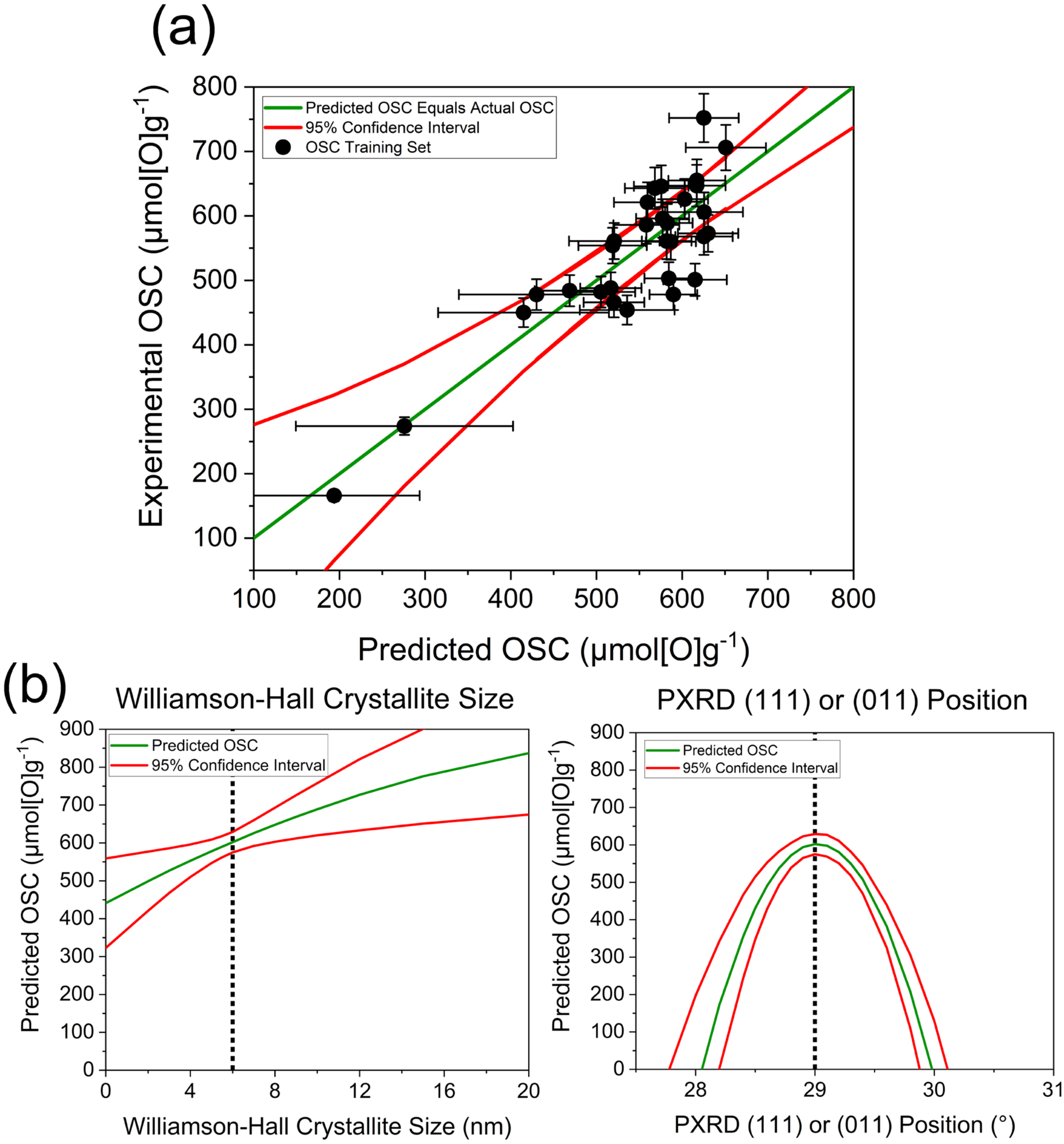

Fig. S38. (a) Predicted OSC against experimentally measured OSC for the PXRD-only OSC prediction model, for a total of 30 ceria-zirconia materials in the training set (compositions given in Table S4). The general formula of the training set compositions is $Ce_xZr_{1-x-y}Ln_yO_{2-\delta}$ (where Ln = La, Y, Pr, Nd for 1 dopant systems or Ln = La-Y and La-Nd for 2 dopant systems). The red lines give the 95% confidence interval of the predictions. (b) Profile traces of the parameters within the model (green line), giving the predicted OSC value as the parameter is changed over the full range of the x-axis, while the other parameters are held constant at values indicated by a dotted line in the respective plots. The 95% confidence intervals are also given as red lines.

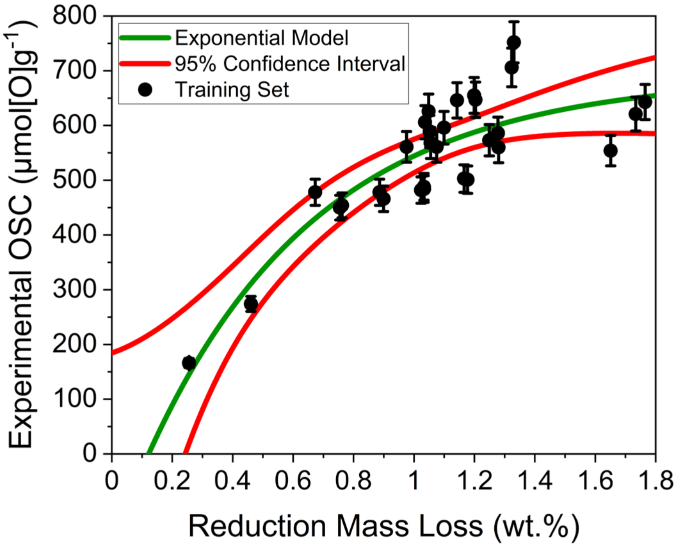

Fig. S39. Experimentally measured OSC against Mass loss during reduction using TGA for the 30 ceria-zirconia materials in the 30-sample OSC training set (compositions given in Table S4), with an exponential prediction model (green line) and a 95% confidence interval (red lines). The general formula of the training set compositions is $Ce_xZr_{1-x-y}Ln_yO_{2-\delta}$ (where Ln = La, Y, Pr, Nd for 1 dopant systems or Ln = La-Y and La-Nd for 2 dopant systems).

D.11. PXRD-only Model Statistical Output

Below is the statistical output for the simple PXRD-only multiple linear regression OSC prediction model shown in Figure S38, containing the Williamson-Hall crystallite size and PXRD peak position ((111) for cubic and pseudo-cubic materials, and (011) for tetragonal structured materials) variables. The summary of fit, analysis of variance, and parameter estimates are given below.

Table S23. Summary of fit for the PXRD-only OSC prediction model given in Figure S38. A full description of each term in this table is given in section A.4.2.

**RSquare**	0.759328
**RSquare Adj**	0.720821
**Root Mean Square Error**	62.01868
**Mean of Response**	542.4667
**Observations (or Sum Wgts)**	30

Table S24. Analysis of variance for the PXRD-only OSC prediction model given in Figure S38. A full description of each term in this table is given in section A.4.2.

Source	DF	Sum of Squares	Mean Square	F Ratio
**Model**	4	303381.55	75845.4	19.7190
**Error**	25	96157.92	3846.3	**Prob > F**
**C. Total**	29	399539.47		<.0001

Table S25. Parameter estimates for the coefficients of each term within the expression of the PXRD-only OSC prediction model given in Figure S38. A full description of each term in this table is given in section A.4.2.

Term	Estimate	Std Error	t Ratio	Prob>|t|
**Intercept**	3674.7207	1160.629	3.17	0.0040
**Williamson-Hall Crystallite Size (nm)**	22.24173	7.611793	2.92	0.0073
**PXRD (111) or (011) Peak Position**	-110.2685	39.78688	-2.77	0.0104
**(Williamson-Hall Crystallite Size (nm)-7.55467)*(Williamson-Hall Crystallite Size (nm)-7.55467)**	-0.501265	0.220513	-2.27	0.0319
**(PXRD (111) or (011) Peak Position-29.102)*(PXRD (111) or (011) Peak Position-29.102)**	-644.5988	96.93374	-6.65	<.0001

D.12. TGA-only Model Statistical Output

Below is the statistical output for linear, quadratic, and exponential function fits of experimental OSC against mass loss during reduction for the 30 training set samples listed in table S4. The general formula of the training set compositions is $Ce_xZr_{1-x-y}Ln_yO_{2-\delta}$ (where Ln = La, Y, Pr, Nd for 1 dopant systems or Ln = La-Y and La-Nd for 2 dopant systems). For each model function trialled, the AICc and BIC (Akaike information criterion and the Bayesian information criterion), SSE (sum of squares error), MSE (mean square error), root mean square error (RMSE) and the R^2 is provided. The SSE is the sum of squared residuals for the fit and the MSE is the SSE divided by the degrees of freedom for error[10]. The root mean square error (RMSE) is the standard deviation of the residuals errors in model predictions and R^2 indicates how much variance around the mean is accounted for by the model fit[10]. The better model fit will minimise the AICc, BIC, SSE, MSE and RMSE while maximising R^2.

(a)

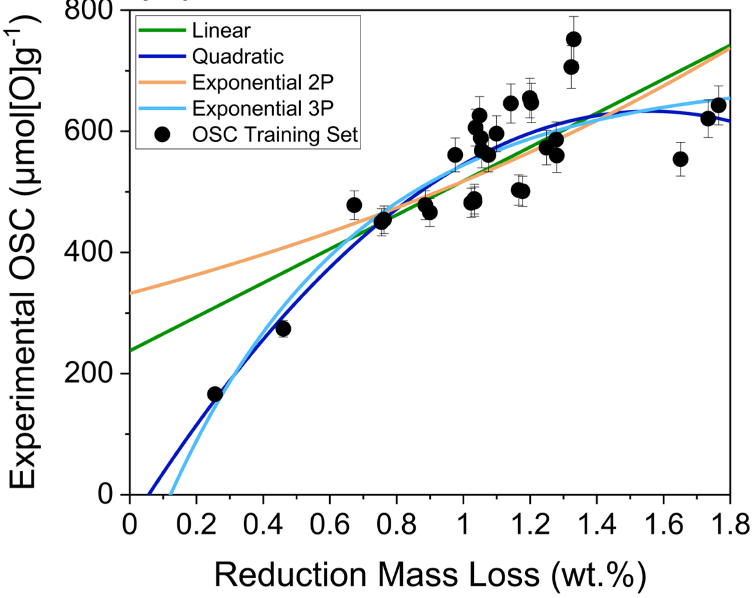

(b)

Model	AICc	AICc Wt	BIC	SSE	MSE	RMSE	R^2
**Quadratic**	336	0.73	340	92788	3437	59	0.77
**Exponential 3P**	338	0.27	342	99127	3671	61	0.75
**Linear**	350	6.91e-4	353	161351	5763	76	0.60
**Exponential 2P**	355	4.11e-5	359	194748	6955	83	0.51

Fig. S40. (a) Experimentally measured OSC against mass loss during reduction from TGA measurements for the 30 ceria-zirconia OSC training set materials (listed in Table S4). Quadratic (dark blue), exponential 2P (general formula $y = b \cdot e^{\lambda x}$, orange), linear (green) and exponential 3P (general formula $y = a + b \cdot e^{\lambda x}$, cyan) are all given here. (b) Statistical analysis from the fits in (a).

D.13. Validation of OSC Prediction Models

Prediction models for oxygen storage were validated using the set of materials listed in the table below.

Table S26. For each of the 10 samples in the validation set, corresponding values for Ce content, total dopant content, Williamson-Hall crystallite, mass loss during reduction and OSC are given. The predicted OSC values are also given using the OSC prediction model in Figure 10 in the main text.

Composition	Ce Content (at.%)	Total Dopant Content (at.%)	Williamson-Hall Crystallite Size (nm)	Reduction Mass Loss (Wt.%)	Experimental OSC (µmol[O]g^{-1})	Predicted OSC (µmol[O]g^{-1})
$Ce_{0.072}Zr_{0.828}La_{0.050}Nd_{0.050}O_{2-\delta}$	7.2	10	8.68(10)	0.803(40)	212(11)	276(64)
$Ce_{0.108}Zr_{0.792}La_{0.050}Y_{0.050}O_{2-\delta}$	10.8	10	8.65(20)	0.706(35)	309(16)	302(45)
$Ce_{0.222}Zr_{0.518}La_{0.260}O_{2-\delta}$	22.2	26	4.97(5)	0.678(34)	411(21)	330(48)
$Ce_{0.249}Zr_{0.581}La_{0.050}Nd_{0.120}O_{2-\delta}$	24.9	17	3.96(3)	1.114(56)	496(25)	445(34)
$Ce_{0.267}Zr_{0.623}La_{0.090}Y_{0.020}O_{2-\delta}$	26.7	11	4.63(8)	1.253(63)	567(28)	520(30)
$Ce_{0.392}Zr_{0.498}Pr_{0.110}O_{2-\delta}$	39.2	11	5.38(11)	1.310(66)	653(33)	591(15)
$Ce_{0.470}Zr_{0.470}La_{0.050}Y_{0.010}O_{2-\delta}$	47.0	6	5.63(13)	1.586(79)	725(36)	594(28)
$Ce_{0.485}Zr_{0.485}Nd_{0.030}O_{2-\delta}$	48.5	3	7.62(20)	1.485(74)	739(37)	686(23)
$Ce_{0.700}Zr_{0.190}Nd_{0.110}O_{2-\delta}$	70.0	11	7.59(11)	1.163(58)	549(28)	563(26)
$Ce_{0.750}Zr_{0.150}La_{0.050}Nd_{0.050}O_{2-\delta}$	75.0	10	7.38(8)	1.107(55)	566(28)	541(31)

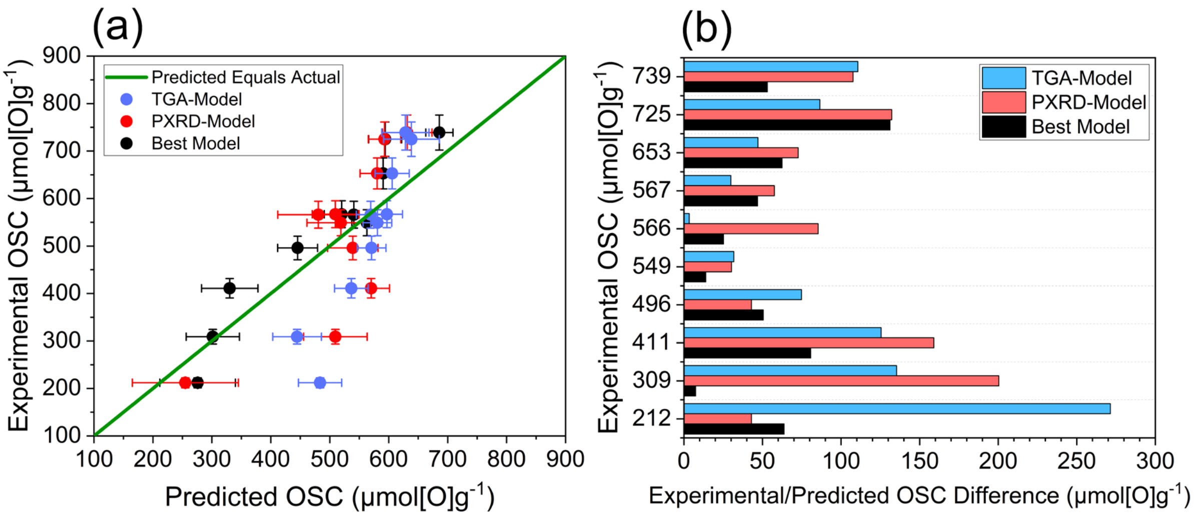

Fig. S41. (a) Predicted OSC against experimentally measured OSC and (b) the absolute difference in the OSC predicted by each model and the OSC measured experimentally for each of the materials in the 10-sample validation set (listed in Table S26), using predictions from the OSC model discussed in the main text and the simpler PXRD-only and TGA-only models discussed in the supplementary sections D10 to D12.

D.14. Model after Removing the Two Highest OSC Materials from the Training Set

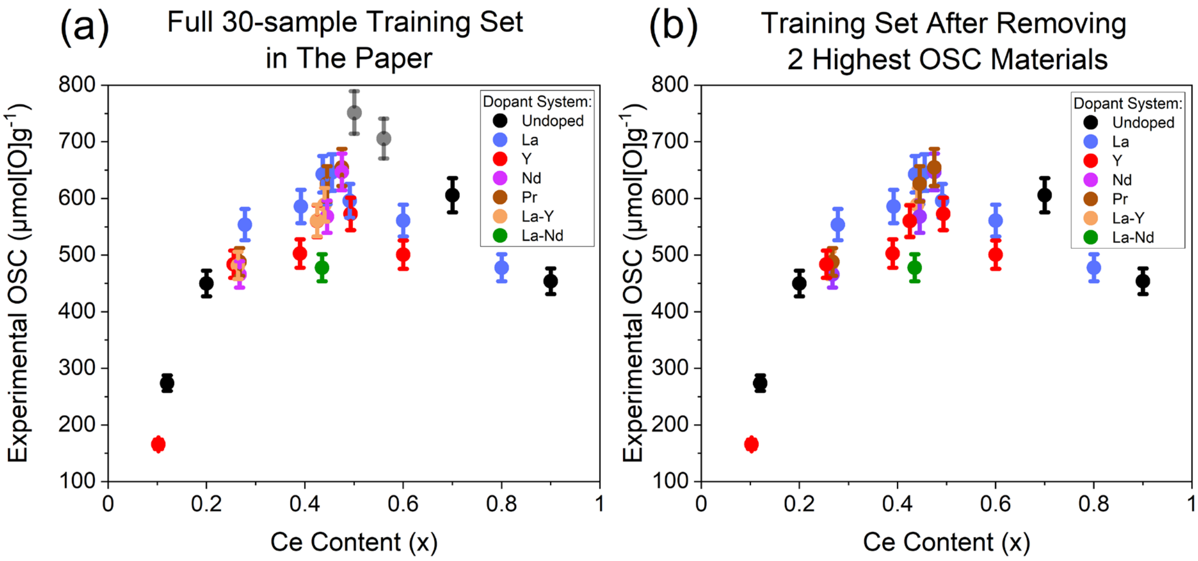

Fig. S42. The measured OSC for each of the compositions in the (a) 30-sample training set in Table S4 and in (b) the training set after removing the 2 largest OSC materials from the full training set (transferring to the validation set).

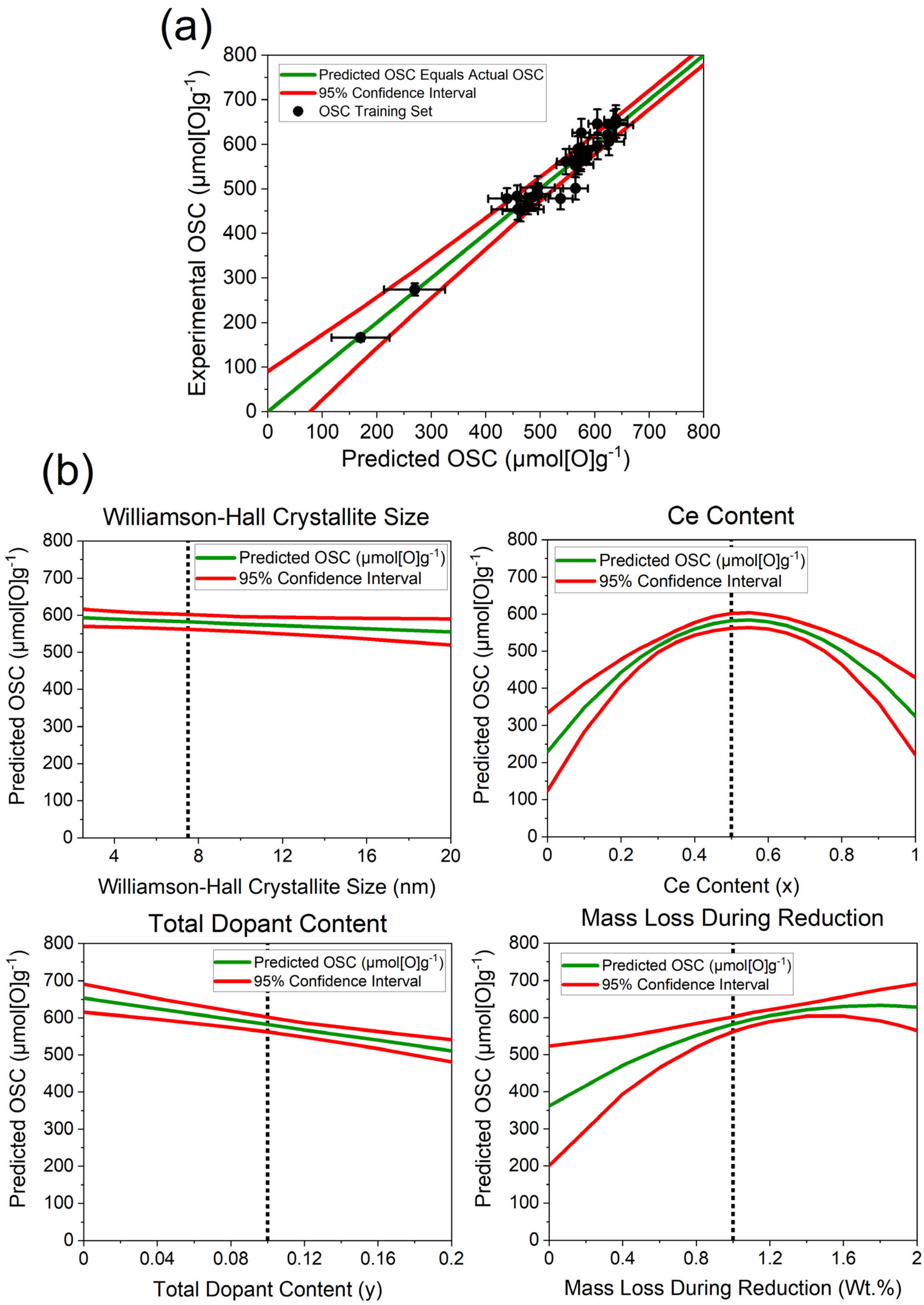

Fig. S43. (a) Predicted OSC against experimentally measured OSC using a multiple linear regression model incorporating dopant content, Ce content, TGA reduction mass loss and Williamson-Hall crystallite size variables after removing the 2 highest OSC materials from the full 30-sample training set, given in Figure S42. The general formula of the training set compositions is $Ce_xZr_{1-x-y}Ln_yO_{2-\delta}$ (where Ln = La, Y, Pr, Nd for 1 dopant systems or Ln = La-Y and La-Nd for 2 dopant systems). The red lines give the 95% confidence interval of the predictions. (b) Profile traces of the parameters within the model (green line), giving the predicted OSC value as the parameter is changed over the full range of the x-axis, while the other parameters are held constant at values indicated by a dotted line in the respective plots. The 95% confidence intervals are also given as red lines.

Table S27. Summary of fit table for the OSC prediction model given in Figure S43. A full description of each term in this table is given in section A.4.2.

**RSquare**	0.949716
**RSquare Adj**	0.93535
**Root Mean Square Error**	27.84989
**Mean of Response**	529.1429
**Observations (or Sum Wgts)**	28

Table S28. Analysis of variance table for the OSC prediction model given in Figure S43. A full description of each term in this table is given in section A.4.2.

Source	DF	Sum of Squares	Mean Square	F Ratio
**Model**	6	307633.49	51272.2	66.1052
**Error**	21	16287.94	775.6	**Prob > F**
**C. Total**	27	323921.43		<.0001

Table S29. Parameter estimates table for the coefficients from each term within the expression of the OSC prediction model in Figure S43 are given. A full description of each term in this table is given in section A.4.2.

Term	Estimate	Std Error	t Ratio	Prob>|t|
**Intercept**	420.23398	40.70295	10.32	<.0001
**Ce Content (at.% / 100)**	268.81293	46.72112	5.75	<.0001
**Total Dopant Content (at.%)**	-7.070958	1.311683	-5.39	<.0001
**TGA Reduction mass loss (wt.%)**	120.74865	28.52368	4.23	0.0004
**Williamson-Hall Crystallite Size (nm)**	-2.148571	1.062514	-2.02	0.0561
**(Ce Content (at.% / 100)-0.42929)*(Ce Content (at.% / 100)-0.42929)**	-1219.171	215.5387	-5.66	<.0001
**(TGA Reduction mass loss (wt.%)-1.07038)*(TGA Reduction mass loss (wt.%)-1.07038)**	-87.32539	41.81918	-2.09	0.0491

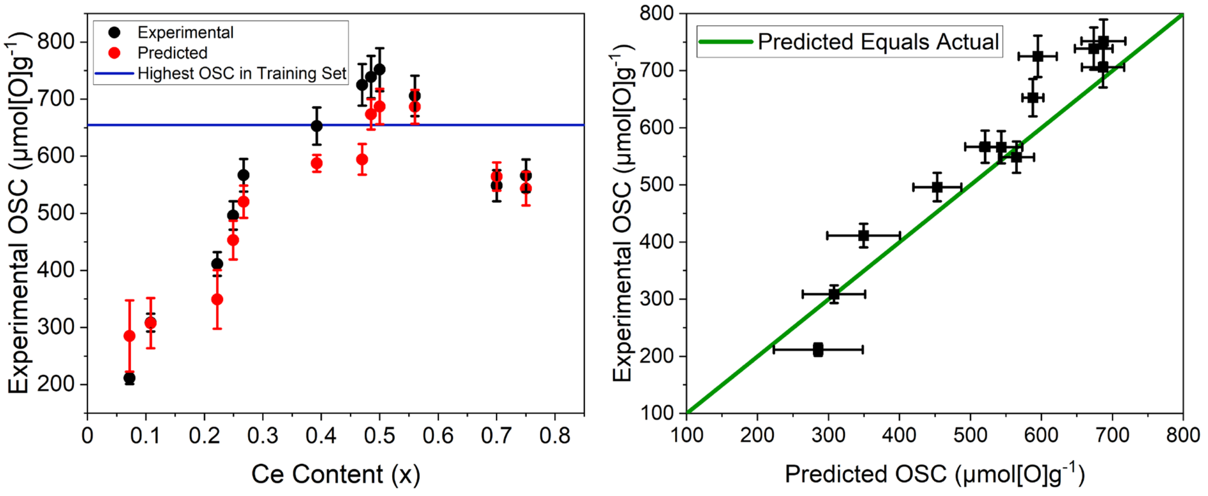

Fig. S44. (a) Measured (black) and predicted OSC (red) against Ce content for OSC predictions from the OSC prediction model trained on the samples after removing the two highest OSC materials from the full training set in Figure S42b ($Ce_xZr_{1-x-y}Ln_yO_{2-\delta}$ samples for validation are taken from the full validation set in Table S4 and the 2 samples removed from the full training set). The horizontal blue line indicates the largest OSC observed for materials in the training set. (b) Experimental OSC values are plot against the predicted OSC values from the OSC prediction model trained on the samples after removing the two highest OSC materials from the full training set in Figure S42b.

D.15. Model after Removing La containing OSC Materials from the Training Set

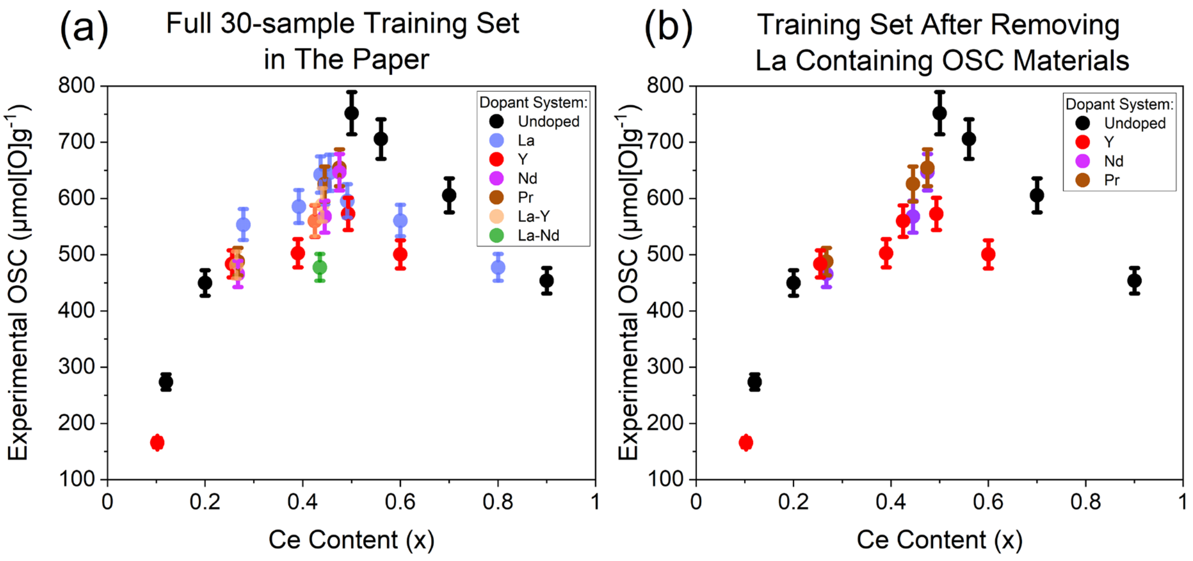

Fig. S45. The measured OSC for each of the compositions in the (a) 30-sample training set in Table S4 and in (b) the training set after removing all La containing compositions.

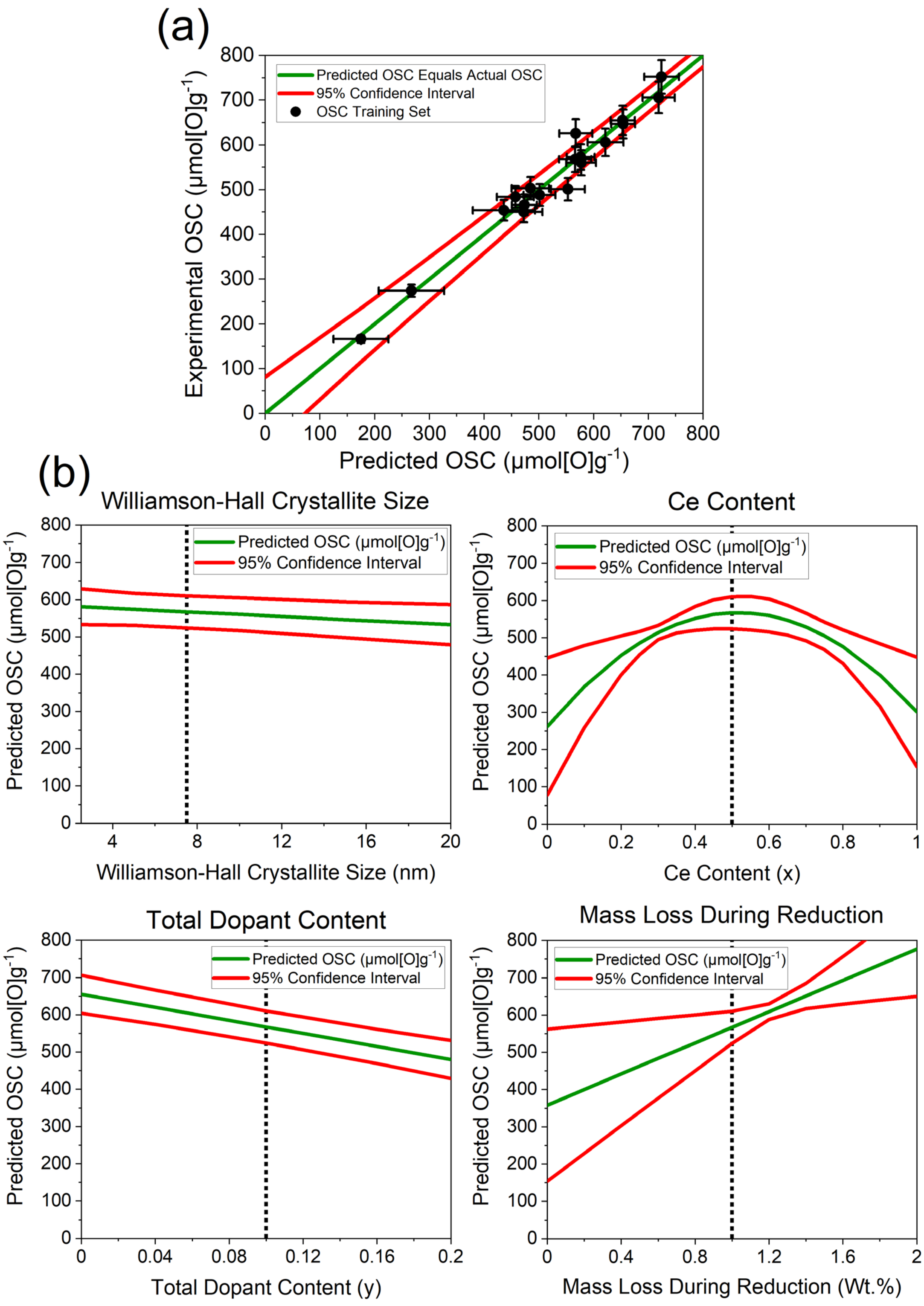

Fig. S46. (a) Predicted OSC against experimentally measured OSC using a multiple linear regression model incorporating dopant content, Ce content, TGA reduction mass loss and Williamson-Hall crystallite size variables after removing the materials containing La from the full 30-sample training set, given in Figure S45. The general formula of the training set compositions is $Ce_xZr_{1-x-y}Ln_yO_{2-\delta}$ (where Ln = La, Y, Pr, Nd for 1 dopant systems or Ln = La-Y and La-Nd for 2 dopant systems). The red lines give the 95% confidence interval of the predictions. (b) Profile traces of the parameters within the model (green line), giving the predicted OSC value as the parameter is changed over the full range of the x-axis, while the other parameters are held constant at values indicated by a dotted line in the respective plots. The 95% confidence intervals are also given as red lines.

Table S30. Summary of fit table for the OSC prediction model given in Figure S46. A full description of each term in this table is given in section A.4.2.

**RSquare**	0.971455
**RSquare Adj**	0.959561
**Root Mean Square Error**	28.78417
**Mean of Response**	526.6111
**Observations (or Sum Wgts)**	18

Table S31. Analysis of variance table for the OSC prediction model given in Figure S46. A full description of each term in this table is given in section A.4.2.

Source	DF	Sum of Squares	Mean Square	F Ratio
**Model**	5	338363.93	67672.8	81.6783
**Error**	12	9942.34	828.5	**Prob > F**
**C. Total**	17	348306.28		<.0001

Table S32. Parameter estimates table for the coefficients from each term within the expression of the OSC prediction model in Figure S46 are given. A full description of each term in this table is given in section A.4.2.

Term	Estimate	Std Error	t Ratio	Prob>|t|
**Intercept**	366.02396	66.58298	5.50	0.0001
**Ce Content (at.% / 100)**	213.98503	88.75601	2.41	0.0329
**Total Dopant Content (at.%)**	-8.732865	1.241698	-7.03	<.0001
**Williamson-Hall Crystallite Size (nm)**	-2.77546	1.123226	-2.47	0.0294
**TGA Reduction mass loss (wt.%)**	209.3023	75.30324	2.78	0.0167
**(Ce Content (at.% / 100)-0.42328)*(Ce Content (at.% / 100)-0.42328)**	-1143.068	356.7664	-3.20	0.0076

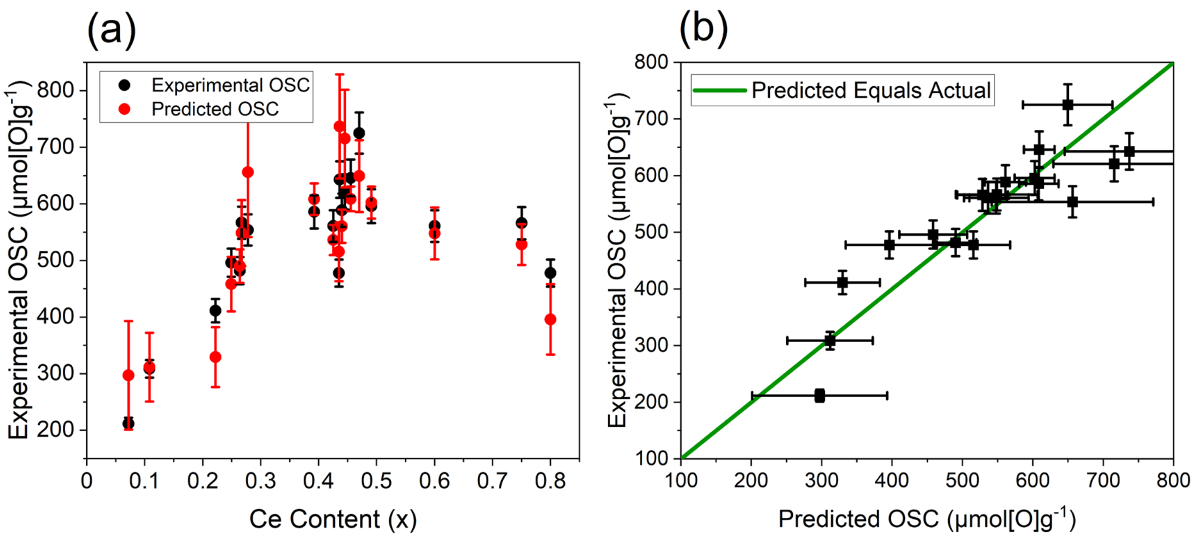

Fig. S47. (a) Measured (black) and predicted OSC (red) against Ce content for OSC predictions on only the La containing compositions (La, La-Y and La-Nd) in the original training and validation sets from an OSC prediction model trained on the samples in Figure S45b, containing only undoped, Y-doped, Pr-doped and Nd-doped systems, allowing the ability of the model to predict an unseen lanthanide (La) to be investigated. (b) Experimental OSC values plotted against the predicted OSC values from the OSC prediction model trained on the samples in Figure S45b, for a validation set containing only the La containing compositions (La, La-Y and La-Nd) in the original training and validation sets.

D.16. Predicted OSC of Screened Compositions

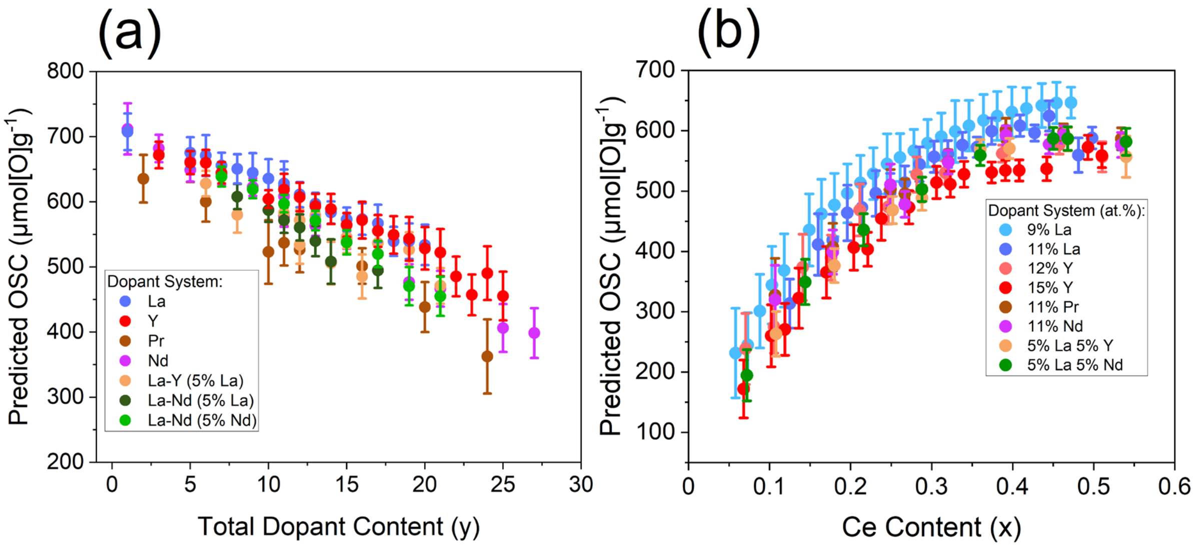

Fig. S48. OSC predictions using the OSC prediction model in Figure 10 in the main text, on the libraries of materials where (a) the stated lanthanide dopant content (y) is increasing while the Ce/Zr ratio is held constant at 1, with the general formula $Ce_{(1-y)/2}Zr_{(1-y)/2}Ln_yO_{2-\delta}$, and where (b) the Ce content (x) is varied while the stated lanthanide dopant content (y) is kept constant, with the general formula $Ce_xZr_{1-x-y}Ln_yO_{2-\delta}$.

E. References

1. B. Zhao, Q. Y. Wang, G. F. Li and R. X. Zhou, Effect of synthesis condition on properties of $Ce_{0.67}Zr_{0.33}O_2$ mixed oxides and its application in Pd-only three-way catalysts, *J. Alloys Compd.*, 2010, **508**, 500-506.
2. L. Lan, S. Chen, Y. Cao, M. Zhao, M. Gong and Y. Chen, Preparation of ceria–zirconia by modified coprecipitation method and its supported Pd-only three-way catalyst, *J. Colloid Interface Sci.*, 2015, **450**, 404-416.
3. Q. Zhong, M. Cui, M. Yue, Q. Wang, L. Wang, R. Guo, Z. Long and X. Huang, Effects of precipitate aging time on the cerium-zirconium composite oxides, *J. Rare Earths*, 2014, **32**, 1010-1015.
4. S. Jabir and K. Harbbi, *A comparative study of Williamson-Hall method and size-strain method through X-ray diffraction pattern of cadmium oxide nanoparticle*, 2020.
5. M. Ghasemi Hajiabadi, M. Zamanian and D. Souri, Williamson-Hall analysis in evaluation of lattice strain and the density of lattice dislocation for nanometer scaled ZnSe and ZnSe:Cu particles, *Ceram. Int*, 2019, **45**, 14084-14089.
6. K. A. Aly, N. M. Khalil, Y. Algamal and Q. M. A. Saleem, Estimation of lattice strain for zirconia nano-particles based on Williamson- Hall analysis, *Mater. Chem. Phys.*, 2017, **193**, 182-188.
7. B. Walczak and D. L. Massart, in *Data Handling in Science and Technology*, ed. B. Walczak, Elsevier, 2000, vol. 22, pp. 323-349.
8. K. P. Burnham and D. R. Anderson, Multimodel inference - understanding AIC and BIC in model selection, *Sociol. Methods. Res.*, 2004, **33**, 261-304.
9. J. Sall, *JMP start statistics : a guide to statistics and data analysis using JMP Sixth edition*, SAS Institute, 2017.

10. J. Sall, M. Stephens, A. Lehman and S. Loring, *JMP Start Statistics, 6th Edition Sall, John*, SAS Institute, 6th edition edn., 2017.
